# New Insights into How *Yersinia pestis* Adapts to Its Mammalian Host during Bubonic Plague

**DOI:** 10.1371/journal.ppat.1004029

**Published:** 2014-03-27

**Authors:** Elizabeth Pradel, Nadine Lemaître, Maud Merchez, Isabelle Ricard, Angéline Reboul, Amélie Dewitte, Florent Sebbane

**Affiliations:** 1 Equipe Peste et *Yersinia pestis*; INSERM U1019, Lille, France; 2 Centre National de la Recherche Scientifique UMR8204, Lille, France; 3 Institut Pasteur de Lille, Centre d'Infection et d'Immunité de Lille, Lille, France; 4 Univ Lille Nord de France, Lille, France; 5 UDSL, Centre d'Infection et d'Immunité de Lille, Lille, France; 6 CHU Lille, Lille, France; National Institute of Allergy and Infectious Diseases, United States of America

## Abstract

Bubonic plague (a fatal, flea-transmitted disease) remains an international public health concern. Although our understanding of the pathogenesis of bubonic plague has improved significantly over the last few decades, researchers have still not been able to define the complete set of *Y. pestis* genes needed for disease or to characterize the mechanisms that enable infection. Here, we generated a library of *Y. pestis* mutants, each lacking one or more of the genes previously identified as being up-regulated *in vivo*. We then screened the library for attenuated virulence in rodent models of bubonic plague. Importantly, we tested mutants both individually and using a novel, “per-pool” screening method that we have developed. Our data showed that in addition to genes involved in physiological adaption and resistance to the stress generated by the host, several previously uncharacterized genes are required for virulence. One of these genes (*ympt1.66c*, which encodes a putative helicase) has been acquired by horizontal gene transfer. Deletion of *ympt1.66c* reduced *Y. pestis*' ability to spread to the lymph nodes draining the dermal inoculation site – probably because loss of this gene decreased the bacteria's ability to survive inside macrophages. Our results suggest that (i) intracellular survival during the early stage of infection is important for plague and (ii) horizontal gene transfer was crucial in the acquisition of this ability.

## Author Summary

In order to understand and combat infectious diseases, it is essential to characterize the full set of genes required by pathogenic bacteria to overcome the many immunological and physiological challenges encountered during infection. Here, we used a genome-scale approach to identify genes required by the bacterium *Yersinia pestis* in the production of bubonic plague (a fatal, flea-borne zoonosis). Our results suggest that when colonizing the mammalian host, the bacterium (i) relies on carbohydrates as its carbon source, (ii) shifts to anaerobic respiration or fermentation and (iii) experiences and resists several (but not all) types of stress generated by the host's innate immune system. Strikingly, only a small set of genes (including horizontally acquired and uncharacterized sequences) are required for these infectious processes. Further investigations of the *ypmt1,66c* gene provided evidence to suggest that accretion of genetic material via horizontal transfer has played a key role in *Yersinia pestis*' ability to successfully initiate infection after the dermal fleabite. Lastly, we believe that (i) application of our approach to other pathogens and (ii) additional studies of selected *Yersinia pestis* genes important for plague pathogenesis (some of which are shared with other pathogens) will provide a better understanding of bacterial pathogenesis in general.

## Introduction

Plague (caused by the bacterium *Yersinia pestis*) is one of the diseases that has most greatly affected the course of history [1], [2] and is still an international public health concern. The WHO has even categorized this disease as re-emergent in some parts of the world. This problem is heightened by the emergence of multidrug-resistant strains and the potential use of plague as a bioweapon [3], [4]. Plague is commonly acquired through the bite of an infected flea, when *Y. pestis* is regurgitated into the dermis during the flea blood meal [5]. It is thought that *Y. pestis* is then taken up by macrophages, within which it replicates and produces antiphagocytic factors [6]. Next, bacteria released from dying macrophages spread into the draining lymph node, where they actively replicate, circumvent phagocytosis by polymorphonuclear neutrophils (PMNs) and confront a number of toxic effectors released by degranulating or lysing PMNs [7], [8]. These events lead to a hemorrhagic, edematous, swollen and painful lymph node (the bubo), which is the defining clinical feature in the bubonic form of plague [9], [10]. Overwhelmed, the lymph node allows the bacteria to disseminate into the blood stream; this produces a fulminant, systemic infection and fatal septicemia. In a very low proportion of infections, septicemia causes pneumonia, which in turn enables *Y. pestis* to be transmitted from person to person *via* contaminated aerosols. However, we lack important data on the above-mentioned mechanisms. For instance, genes dedicated to the intracellular step have not been identified. Furthermore, recent work suggests that transit through the flea vector enhances *Y. pestis*' ability to resist phagocytosis by the macrophage [11]. Very few pathways for resisting innate immune effectors have been elucidated [12]. Lastly, the metabolic pathways that enable *Y. pestis* to replicate during infection are incompletely understood.

Regurgitation of *Y. pestis* into the skin does not always cause bubonic plague and bubonic plague does not always progress consistently to septicemic plague. This indicates that the skin and the lymph node are the two decisive arenas in which *Y. pestis* must deploy its arsenal to produce a successful infection. Better knowledge of this arsenal will undoubtedly improve our understanding of the pathogenesis of plague and should help us to develop better countermeasures against this disease. To this end, we previously determined the transcriptome of *Y. pestis* in the most relevant context of infection: the rat bubo [7]. Although the study results were valuable, they were not predictive of the requirements for virulence and merely provided clues about putative biological roles of upregulated genes *in vivo* that will have to be confirmed using traditional experimental approaches. We therefore pursued our efforts by devising a means of screening a mutant *Y. pestis* library in which genes previously identified as being up-regulated *in vivo* had been deleted. This method enabled us to draw up a hierarchical table ranking genes in order of their importance in disease production. The results of further investigations suggest that horizontal gene acquisition had a key role in establishing *Y. pestis*' ability to successfully initiate infection after the dermal fleabite.

## Methods

### Ethics statement

Animals were housed at the Institut Pasteur de Lille animal facility (accredited by the French authorities for the performance of experiments on live rodents (#B59-350009) and which complies with French and European regulations on the care and protection of laboratory animals (EC Directive 86/609 and the French Act #2001-486, issued on June 6, 2001)). At the time some experiments were performed, regulations did not require ethical approval. However, all animal experiments had been authorized by the French veterinary authorities (#59-350218) and were performed in compliance with the terms of NIH Animal Welfare Assurance #A5476-01 (issued on July 2, 2007). Since 2013, animal experiments were approved by the Nord-Pas-de-Calais Ethics board committee (#CEEA 23012). Human serum samples were obtained after the provision of written, informed consent by the donors. The use of human samples has been declared and approved by the MESR and CPP boards (#DC-2013-1765).

### The strains, the mutant library and the plasmids

The bacterial strains and plasmids used in this study are presented in Table S1 and Text S1. Mutant *Y. pestis* strains were generated using allelic exchange methods (based on lambda Red recombinase or the pCVD442 suicide vector [13], [14]) and with the primer sets and plasmids described in Tables S1 and S2. The coding sequence of interest (apart from the first and last 50 base pairs) was replaced by a kanamycin, zeocin or trimethoprim resistance gene that had been amplified from the suicide vectors listed in Table S1. Each mutant's genotype was confirmed by PCR assays using primer sets that were complementary to the target flanking sequence not involved in the allelic exchange (Table S2). Mutant strains were complemented with the recombinant pCRII plasmid (Life technologies, Saint Aubin, France) containing a wild-type (WT) copy of the gene of interest (including the latter's putative promoter sequence).

### Determination of bacterial virulence in rodent models of bubonic plague

Virulence attenuation was evaluated in either pools of mutants or individual mutants. Wild-type and mutant strains were cultured in Luria broth (LB) at 21°C, quantified (via the optical density at 600 nm), diluted to the desired cell density in sterile phosphate-buffered saline (PBS) and mixed with equal colony-forming unit (CFU) counts of other mutants if required. Fifty microliters of the bacterial suspension were intradermally inoculated (as previously described [9]) into 8-week-old female Brown-Norway rats (Janvier, France) or OF-1 female mice (Charles River, France). For individual screening, rats and mice were inoculated with 20 and 10 CFUs respectively. For per-pool screening, rats were injected with a total of 100 CFUs (20 CFUs of each of five mutants). Groups of eight and ten animals were respectively used for individual and per-pool screening experiments. For per-pool screening, animals were euthanized when signs of terminal plague appeared. The inguinal lymph nodes proximal to the inoculation site and the spleens were immediately collected after euthanasia and then triturated. Serial dilutions of the triturated organs were plated on blood agar containing the antibiotics of interest. The CFUs were counted after a 48-hour incubation at 28°C. The virulence attenuation of *Y. pestis* was measured by calculating the average relative competitive index (ARCI), as follows:
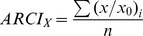
 where 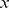 is the CFU count of the tested mutant, 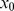 is the count for the most abundant mutant (i.e. the strain that acts like a WT strain in the animal of interest), 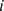 is the animal of interest, 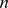 is the number of animals used in the experiment, and 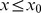. Groups of five 8-week-old female Brown-Norway rats (inoculated as above) were used to study the time course of lymph node, blood and spleen colonization by bacteria. Lastly, in order to study the role of PMNs cells during infection, groups of 8-week-old OF-1 mice were inoculated intraperitoneally with 100 µg of anti-GR1+ (RB6-8C5) antibody (as described above) on the day before the intradermal challenge [8].

### Growth in human serum

*Y. pestis* grown overnight in LB at 21°C were centrifuged, washed and adjusted to 2×10^7^ bacteria/mL in PBS. Twenty microliters of the bacterial suspension were added to 180 µl of fresh human serum in the wells of a 96-well plate. After different incubation times at 37°C, serial dilutions of the serum were plated on blood agar and the CFUs were subsequently quantified.

### Bacterial survival in macrophages

Macrophages were cultured in high-glucose (4.5 g/L) Dulbecco's Modified Eagle Medium (DMEM) supplemented with 5% heat-inactivated fetal bovine serum. All incubations were performed at 37°C in a 5% CO_2_ atmosphere. In the intracellular survival assay, cells were infected as previously described [15]. The day before interaction with *Y. pestis*, 1.4×10^5^ RAW macrophages in the incubation medium were allowed to seed in the wells of a 24-well plate. The macrophages were washed three times in incubation medium prior to addition of *Y. pestis* that had been grown overnight at 21°C in LB, washed and suspended in DMEM. The multiplicity of infection was 10. Immediately after mixture of the bacterial suspension and the macrophages, the plates were centrifuged (at 18 g for 5 minutes) and then incubated. After 30 minutes, the cells were washed three times. Fresh medium supplemented with 8 µg/mL gentamicin was then added. One hour later, the wells were washed twice and fresh medium containing 2 µg/mL of gentamicin was added. At various time points after contact, macrophages were lysed by incubation in 500 µl of cold water for 10 minutes on ice. The lysate was plated on blood agar and the number of CFUs was counted.

### Statistical analysis

A mutant was considered to be virulence-affected when the survival curves for animals infected with the mutant and the wild-type strain were found to be significantly different (p<0.05) in a Gehan-Breslow-Wilcoxon test. Two-way analysis of variance was also used to compare data obtained from *in vitro* experiments; a p<0.05 was considered significant.

## Results

### More than 15% of the *Y. pestis* loci up-regulated *in vivo* are required for virulence

**Figure 1:**
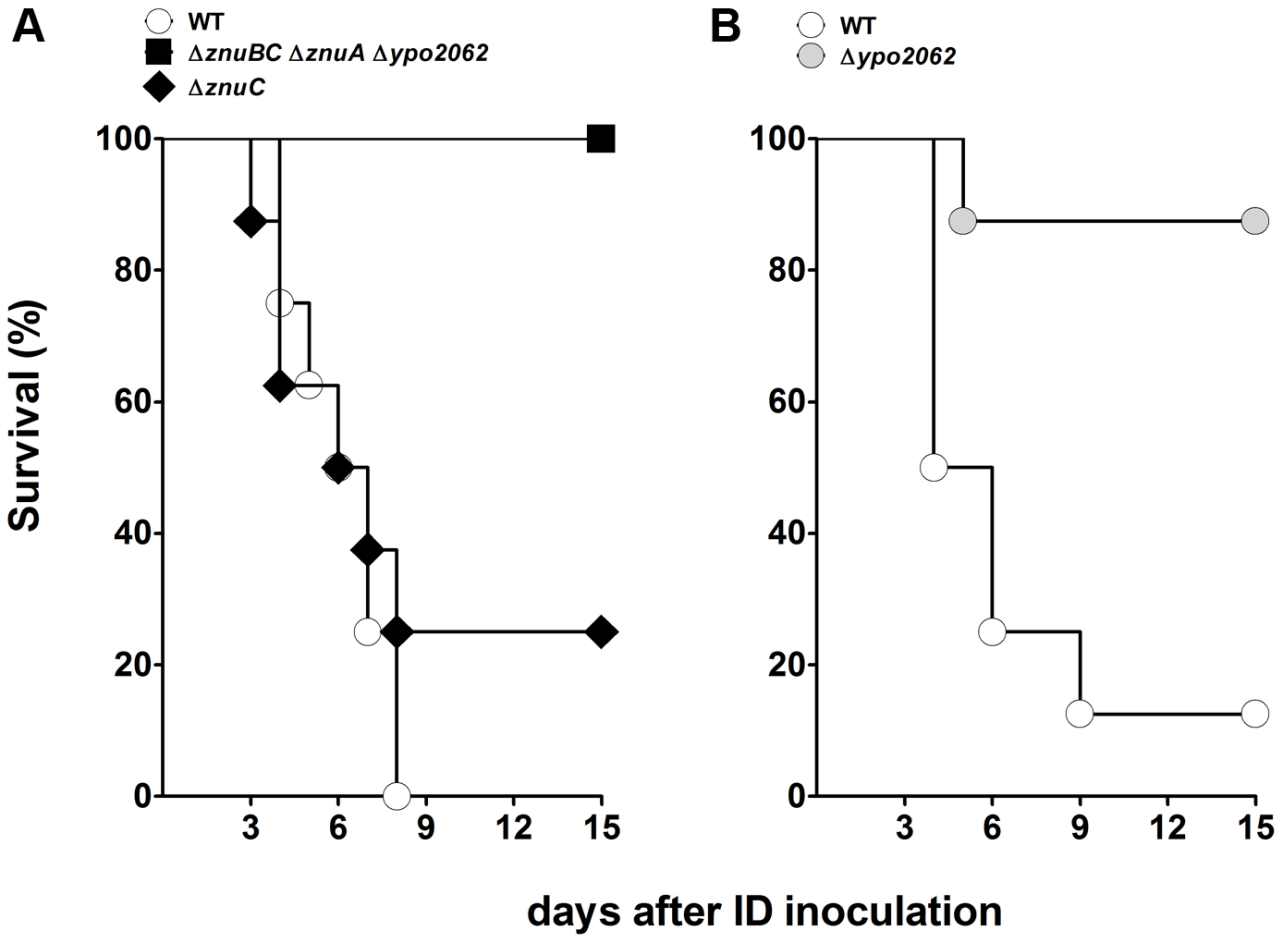
Incidence of plague in mice infected with *Y. pestis* lacking the zinc transporter Znu and/or the putative zinc murein DD-endopeptidase YebA (YPO2062). (A), mice were injected intradermally with ∼10 *Y. pestis* WT (white circles) and Δ*znuABC ypo2062* (black squares) or Δ*znuC* (black diamonds). (B), mice were inoculated with as in (A) with WT (white circles) or Δ*ypo2062* (grey circles) mutants. Only the loss of YPO2062 significantly decreased the virulence of *Y. pestis* (*p*<0.05, relative to WT). Groups of 8 mice were used. Data obtained with the Δ*znuABC ypo2062* mutant are representative of two experiments. Data obtained with the Δ*znuC* and Δ*ypo2062* mutants come from one experiment each.

We previously reported that 526 *Y. pestis* genes (other than previously characterized virulence factors) were up-regulated more than two-fold in the rat bubo, relative to *Y. pestis* cultures grown at 21 or 37°C [7]. Seventy-nine of these are highly likely to encode factors important for plague pathogenesis because they are (i) up-regulated in both the rat bubo and the mouse lung or (ii) thought to be involved in the bacterium's defensive response against nitric oxide, oxidative stress and iron stress generated by the immune system during bubonic plague in rat [7], [16]. We therefore generated a library of *Y. pestis* mutants in which one or more of these 79 genes had been deleted and then tested the individual mutant's virulence in mouse or rat models of bubonic plague. In order to reduce the number of mutants and the number of animals to be sacrificed, we deleted blocks of neighboring genes (whenever possible and regardless of their genetic organization and relationship). Hence, a total of 33 mutants (rather than 79) were produced. The mutant lacking the functional pyruvate dehydrogenase AceEF [17] was the only one to show a serious growth defect on LB agar. As expected, this mutant did not kill any mice 15 days after inoculation with a dose (∼20 CFUs) that usually kills all the animals (Table S3) - indicating that the mutant was virulence-attenuated. Screening of the library for virulence revealed five attenuated mutants (Table S3). The strains lacking the homoserine O-succinyltransferase gene *metA*, the ribonucleoside reductase operon *nrdHIEF* or the uncharacterized gene *ypo0426* required significantly more time to cause fatal plague (median survival ≥5 days *vs* 4 days for the mutants and the wild-type strain respectively). The Δ*ypo0988*, Δ*ptsG* and Δ*znuABC ypo2062* mutants (respectively lacking the genes for an uncharacterized protein, the glucose transport PtsG and both the Zinc (Zn) transporter ZnuABC and the putative zinc murein DD-endopeptidase YebA [18]) caused plague but at a lower incidence (survival rate >60% *vs* ≤12.5 for the mutants and the wild-type respectively). Further virulence assays showed that deletion of *yebA* (but not *znu*) was responsible for the loss of virulence in the Δ*znuABC ypo2062* mutant (Figure 1).

Next, we sought to establish which of the remaining 447 up-regulated genes were required for plague progression in rats. To this end, we used the approach described above to build a library of 137 mutants and designed a per-pool screening method for application in the most relevant model of infection. Hence, rats were intradermally inoculated with about the dose of *Y. pestis* (the input pool) that is regurgitated by the rat flea *Xenopsylla cheopis* (i.e 100 CFUs) [19]. In order to reduce the selection of false positives and false negatives, each mutant in the pool was given at the lowest dose that consistently killed all the rats in experiments with the WT (20 CFUs). In other words, each pool comprised five mutants. A wild-type strain was not included in each pool because (i) we postulated that at least one mutant in the pool would have a wild-type like phenotype (i.e. colonizing and killing the animals as efficiently as the wild type) and (ii) we expected a bottleneck restricting bacterial dissemination from the skin to the other tissues [20]. To overcome bias due to bottlenecking, we were therefore obliged to evaluate each pool in several animals (see below). To identify mutants within a pool, we provided each one with a specific antibiotic resistance marker (kanamycin, trimethoprim or zeocin, denoted respectively as K, T and Z). The mutants were generated using a virulent strain bearing (or not) a zeocin resistance gene at the only neutral *att* Tn*7* chromosomal site (Figure S1). Hence, five groups of tagged mutants were produced (K, T, Z, ZK, and ZT). Each mutant was counted in the lymph nodes and the spleen (the output pool), in order to (i) identify genes that might be necessary during early or late stages of infection and (ii) reduce the likelihood of missing false negatives (i.e. attenuated mutants that might artificially appear to be virulent as a result of transcomplementation of the mutant's defects by other strains in the pool or the production of an abnormal environment that facilitates the growth of an otherwise virulence-attenuated mutant. We expected rescued mutants to be more outcompeted in the spleen than in the lymph node. Hence, we did not expect a virulence-attenuated mutant to be fully rescued in the final “bacterial read” in the spleen - even when it may have been transcomplemented in the pool. Lastly, the bacterial loads for each mutant were determined in animals displaying terminal clinical signs of plague; at this stage of the disease, (i) the bacterial load recovered from an organ (>10^7^ CFU) is high enough to ensure reproducible recovery of each mutant, and (ii) all the mutants would have experienced similar *in vivo* growth conditions because the organs from terminally ill animals described in the literature (and certainly from our experience) were all histologically similar [9], [21]. Using this strategy, a total of 24 pools comprising five mutants and one pool comprising four mutants (i.e. 124 mutants in total) was tested. Due to technical issues, the remaining 13 mutants had to be individually evaluated for virulence. One of the 13 had a longer incubation period in animals and was found to lack the prophage *ypo1089*-*ypo*1091 locus (Table S3).

Terminal signs of plague occurred 2 to 5 days after inoculation of the pools. The mean time to sacrifice was 2.6±0.4 days (Figure S2). At the time of sacrifice, the lymph nodes and the spleens from all rats were heavily colonized, with a median bacterial count of >10^8^ CFU (Figure S3). These data suggested that each pool behaved collectively like a WT strain and that at least one mutant within each pool was fully virulent because i) the time course of infection of animals infected with either ∼20 or ∼100 wild-type CFU did not differ significantly (data not shown) and ii) organs from animals showing terminal signs of plague reportedly contain more than 10^7^ CFU [9], [22], [23]. Analysis of the CFU counts in the output pools revealed that there was a bottleneck that affected bacterial dissemination from the skin to the other tissues. The proportions of clones recovered from the lymph nodes and the spleens did not appear to follow a particular trend; a clone that completely dominated colonization in one animal could be totally outcompeted in another (Figure S4). This variable “dominant effect” hampers the identification of genes needed for microbial pathogenesis [24], [25]. However, we considered that the “dominant effect” observed in individual animals would be balanced by an analysis of the group of animals (notably by averaging the data obtained from the different replicates). Hence, we determined the relative virulence of each mutant for a specific organ by calculating an average relative competitive index (ARCI) for each mutant by using the equation 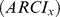 described in the Methods section. Following inspection of the data, we divided the mutants into three virulence classes with either a high to moderate likelihood of being virulence-attenuated (ARCI≤10), a moderate to low likelihood (10<ARCI<20) or no likelihood (ARCI≥20) (Table S4). The first two categories comprised about 40% of the mutants (48 out of 124) (Table 1). Fifteen of the 48 mutants lacked metabolic genes, 5 lacked stress response genes, 2 lacked fimbriae/adhesin genes and 26 lacked uncharacterized genes. Interestingly, some mutants were more outcompeted in the lymph node than in the spleen (or vice-versa), suggesting that specific factors are required to colonize each organ. To confirm the validity of our hierarchical ranking, the individual virulence of a number of mutants from each of the three different ARCI classes (11, 5 and 4 mutants, respectively) was evaluated in the rat. Eight mutants (variously lacking Pap fimbriae, Ail or uncharacterized proteins) were found to be significantly less virulent (Table S4) and all had an ARCI<20. Furthermore, 65% and 20% of the attenuated mutants were from the “high ARCI” and “low ARCI” categories, respectively; this suggests the existence of a correlation between the ARCI category and loss of virulence.

**Table 1:** List of mutants selected using the per-pool screening method.

	MUTANT LACKING			ARCI† ± SE IN			
	ORF(s)	GENE(s)	CATEGORY OF THE GENE	LYMPH NODE		SPLEEN	
**Metabolism**	YPO2303-YPO2302	*pntAB*	Nicotinate and nicotinamide metabolism	0.0	±0	0.0	±0
	YPO2288	*amn*	Purine metabolism	0.0	±0	0.0	±0
	YPO1133	*gpmA*	Glycolysis/Gluconeogenesis	0.0	±0	0.0	±0
	YPO2269	*bioD2*	Biotin metabolism	2.6	±2	12.6	±10
	YPO1139-1136	*galETKM*	Galactose metabolism	3.8	±2	10.2	±10
	YPO3826-YPO3824	*glpABC*	Glycerophospholipid metabolism	5.4	±5	0.8	±0
	YPO1514-YPO1512	*cdd yohJK*	Pyrimidine metabolism	7.1	±5	31.9	±14
	YPO3953-YPO3955	*gntKT y3874 gntR*	Pentose phosphate pathway	11.7	±9	7.9	±5
	YPO2734-YPO2743	*ccmABCDEFGH1H2 vacJ*	Cytochrome c-type biogenesis	12.0	±10	7.7	±3
	YPO0579-YPO0581	*uxaC uxuBA*	Pentose and glucuronate interconversions	14.4	±10	17.4	±10
	YPO3808-YPO3804	*livKHMGF*	Amino-acids Tansport	14.7	±10	16.2	±10
	YPO3719	*lysC*	Amino-acids metabolism	18.2	±11	15.1	±10
	YPO2325	*dalD*	Pentose and glucuronate interconversions	19.1	±11	13.1	±10
	YPO0989-YPO0994	*iucABCD iutA*	Iron transport	20.2	±13	10.1	±10
	YPO2965-YPO2969	*dmsABC*	Respiration	30.5	±14	18.3	±11
**Attachment**	YPO0698-YPO0700	*papCD gafB*	Attachment/colonization	0.3	±0	0.0	±0
	YPO2905	*ail*	Attachment/colonization	8.3	±5	14.2	±8
**Stress response**	YPO2714	*rseC*	Regulation	0.5	±0	0.9	±1
	YPO4085	*ibpA*	Molecular chaperone	0.9	±0	1.6	±1
	YPO1359	*hcr*	NADH-oxydoreductase	14.9	±11	21.2	±11
	YPO2958-YPO2960	*yfuABC*	Stress response	23.0	±13	17.9	±10
	YPO0049	*radC*	Stress response	29.2	±14	18.1	±11
**Uncharacterized**	YPO2586-YPO2587	*-*	Inositol phosphate metabolism	0.0	±0	0.0	±0
	YPO0618-YPO0617	*-*	-	0.1	±0	0.1	±0
	YPMT1,66c	*-*	-	0.5	±0	1.2	±1
	YPO3798-YPO3801	*-*	Attachment/colonization	1.1	±1	1.1	±1
	YPO2561-YPO2560	*-*	-	4.6	±5	1.4	±1
	YPO1992	*-*	-	8.6	±6	15.0	±10
	YPO0337	*-*	-	9.5	±9	5.4	±5
	YPO0656	*yqiC*	-	12.5	±13	0.0	±0
	YPO3369	*-*	-	22.0	±13	4.4	±2
	YpcD1.23	*-*	-	26.2	±12	10.9	±5
	YPO0841-YPO0843	*ydeMN -*	-	11.5	±9	12.5	±10
	YPO3008-YPO3009	*-*	Regulation	13.6	±10	12.6	±10
	YPO3991	*yhjJ*	-	14.5	±10	18.8	±11
	YPO1307	*-*	-	15.2	±9	14.0	±7
	YPO1380	*ycaD*	Transport	15.4	±12	16.0	±12
	YPO3342-YPO3343	*yhjA -*	-	15.5	±10	15.9	±10
	YPO1289-YPO1287	*-*	Amino-acids metabolism	15.6	±9	32.7	±15
	YPO2337	*-*	Regulation	16.7	±10	25.4	±11
	YPO3025	*-*	-	17.8	±10	39.1	±14
	YPO2745	*-*	-	18.9	±11	37.1	±15
	YPO0585	*-*	-	19.3	±11	27.6	±14
	y4094	*-*	-	20.5	±11	17.3	±10
	YPO2006-YPO2008	*-*	-	20.7	±13	18.5	±12
	YPO1862	*-*	-	25.4	±13	16.7	±12
	YPO2282	*-*	-	31.4	±13	15.4	±11
	YPO0856-YPO0852	*-*	Transport & Galactose metabolism	32.1	±15	14.3	±10

† , data were obtained from groups of 10 rats inoculated intradermally with a pool of 5 mutants (with 20 CFU of each mutant).

The attenuation of the mutants described above might conceivably result from a secondary mutation generated during the construction process. To test this hypothesis, we compared the virulence of the wild-type strain and several mutants (Δ*ypo0988*, Δ*ypo2062*, Δ*ypo0656*, Δ*ypo2560-61*, Δ*ypo3369* and Δ*ypo3991*) harboring (or not) a wild-type copy of the gene of interest on a plasmid (Figure S5). In accordance with the results of the above-described infection experiments, all mutants were significantly affected in virulence (p<0.05 in a Gehan-Breslow-Wilcoxon test). The virulence of the complemented mutants Δ*ypo0988*, Δ*ypo2062* and Δ*ypo0656* was fully restored. In contrast, the virulence of the complemented mutants Δ*ypo2560-61*, Δ*ypo3369* and Δ*ypo3391* was not restored - indicating that these mutants carry a secondary mutation or that the methodology used for complementation was inadequate.

### *Y. pestis* requires a limited number of metal acquisition systems to counter metal starvation *in vivo*

Iron, manganese and zinc play a crucial role in many biological processes - including bacterial virulence. Hence, it is not surprising that a marked number of metal acquisition systems are up-regulated by *Y. pestis in vivo* [7], [16]. Notably, *Y. pestis* overexpresses 12 of the 15 proven or potential iron transport systems (Table S5 and Text S1). It has previously been shown that the absence of the Yersiniabactin (Ybt) siderophore-dependent system or the Yfe ATP-binding cassette (ABC) iron transporter (but not that of the heme transporter Hmu, the hemin storage system Hms or the ABC iron transporters Yiu and Yfu) attenuates the virulence of *Y. pestis* in a mouse model of bubonic plague [26], [27], [28], [29], [30]. Our present data (obtained in a different *Y. pestis* strain and a different rodent species and strain) confirmed that Hmu, Hms, Yiu and Yfu are not required for bubonic plague (Table S5). Furthermore, we found that three siderophore-based systems, two ABC ion transporters and two iron permeases were not necessary for disease production, despite upregulation of the corresponding genes *in vivo*; this is presumably because some of these transport systems are not functional in *Y. pestis* due to frameshift mutations or gene disruption by an insertion sequence [31].

*In vivo*, *Y. pestis* also overexpresses the ZnuABC zinc transporter and the MntH manganese transporter (which are also the predominant zinc and manganese importers *in vitro*). However, mutants lacking Znu and MntH were found to be fully virulent (Figure 1 and Table S5), which agrees with recent findings in a different *Y. pestis* strain and a different animal model [32], [33].

### *Yersinia pestis* requires carbohydrate metabolism when colonizing the mammalian host

**Figure 2:**
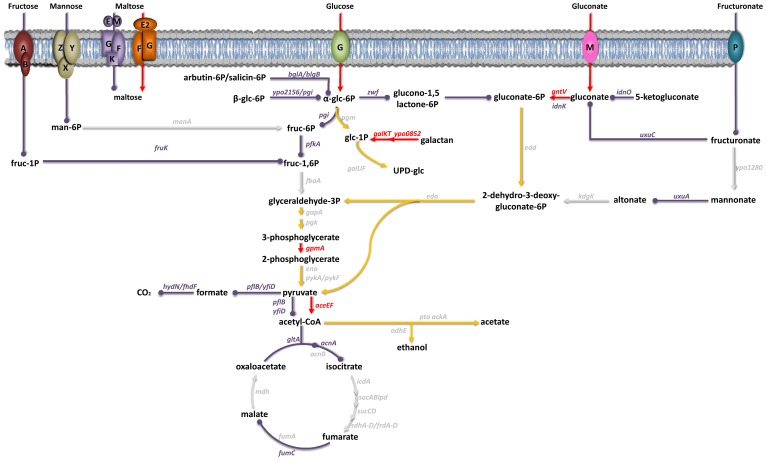
Carbohydrate uptake and metabolism by *Y. pestis* in its mammalian host. Genes encoding transporters for fructose (FruAB), mannose (ManZYX), maltose (MalEMGKF and MalE_2_FG), glucose (PtsG), gluconate (GntM) and fructuronate (GntP) and the carbohydrate metabolism genes shown in red or purple were all overexpressed *in vivo* [7]. In virulence tests, mutants lacking a gene highlighted in red were virulence-attenuated, whereas those lacking a gene highlighted in purple were not. Genes in grey were not upregulated *in vivo* and their role in virulence has yet to be elucidated; hence, the importance of the metabolic fluxes involving the corresponding enzymes in virulence is unknown (grey arrows). Red and purple arrows respectively indicate the metabolic fluxes considered to be important and non-essential for virulence. Yellow arrowheads represent the metabolic pathways that glucose and gluconate might follow.

Inspection of the expression patterns for genes involved in metabolic pathways has suggested that carbohydrates constitute *Y. pestis*' main carbon and energy source during successful colonization of the mammalian host [7]. The results of our virulence tests indicated that the uptake of glucose, gluconate and, to a lesser extent, maltose (but not that of fructose, mannose or fructuronate) was important for plague production (Figure 2 and Table S3 and S4). Glucose is known to be metabolized *via* glycolysis; however, the latter did not appear to be essential for plague production because deletion of the first two enzymes in this pathway (Pgi and PfkA) did not affect *Y. pestis*' virulence. Although the presence of a complete glycolysis pathway was not essential for host colonization, its terminal part appeared to be essential because a *gpmA* mutant was completely outcompeted *in vivo*. The end product of glycolysis (pyruvate) is an important source of acetyl CoA, some of which enters the tricarboxylic acid (TCA) cycle via reaction with citrate synthase. However, this step (and perhaps the TCA cycle as a whole) was not essential for virulence. Thus, *Y. pestis* relies on carbohydrate metabolism to produce disease but carbohydrates such as glucose are not necessarily directed towards the expected metabolic pathways.

### A limited number of genes enable *Y. pestis* to resist nitrosative stress in the bubo

During infection, *Y. pestis* is in close contact with PMNs that produce inducible nitric oxide synthase. The bacterium uses the flavoglobin Hmp to detoxify reactive nitrogen species (RNS) released by the PMNs [7]. Of the *Y. pestis* genes that are up-regulated in the rat bubo and for which homologs in other bacterial species are involved in the response to RNS, *aceE* that we found to be necessary for virulence was also found to be required for optimal bacterial growth *in vitro* (Table S6 and Text S1). Therefore, one can hypothesize that *aceE* is essential for virulence but is not necessarily required to counter the host PMNs' production of nitric oxide. Of the 8 other loci thought to protect *Y. pestis* against RNS *in vivo*, only *nrdHIEF* (the ribonucleotide reductase operon) appeared to contribute moderately to virulence; deletion of this operon was associated with a slightly longer time to disease onset (Table S6).

### Further evidence to suggest that *Y. pestis* may not encounter oxidative stress *in vivo* or may require a limited number of genes to resist oxidative stress

The absence of induction in the rat bubo of (i) the OxyR oxidative stress regulator regulon and (ii) the genes known to protect bacteria against peroxynitrite (i.e. the product of the reaction between hydrogen peroxide and nitric oxide) suggested that *Y. pestis* is not exposed to reactive oxygen species (ROS) in the mammalian host [7]. However, a limited number of genes involved in bacterial resistance against ROS are known to be activated in the rat bubo, and they may encode the major detoxifying proteins. Furthermore, several genes involved in the detoxification of ROS (which are downregulated or at least not upregulated in the rat bubo) were shown to be upregulated in *Y. pestis* replicating inside a macrophage-cell line *in vitro* [34]; macrophage infection is thought to be important in the initial stages of infection [6]. Of the genes known to protect bacteria against oxidative stress, *Y. pestis* overexpresses *mntH*, *fumC*, *yfiD* (*grcA*) and *ibpA in vivo*. As mentioned above, MntH is required for manganese import (which in turn is involved in protection against ROS), whereas FumC and GrcA rescue enzyme reactions when FumA and PflB are damaged by ROS [35], [36], [37]. IbpA protects several metabolic enzymes against oxidation [38]. Deletion of the *ibpA* gene was associated with attenuation of virulence because the mutant was outcompeted in per-pool infection. However, IbpA is also involved in resistance to other forms of stress, such as high temperatures [38], [39]. In contrast, deletion of *fumC*, *yfiD* or *mntH* did not affect the virulence of *Y. pestis* (Table S6). Thus, our data provide further evidence for the hypothesis whereby ROS production may not be a significant host defense factor in plague.

### Horizontal genetic transfer had an important role in acquisition of *Y. pestis* capacity for intracellular survival

**Figure 3:**
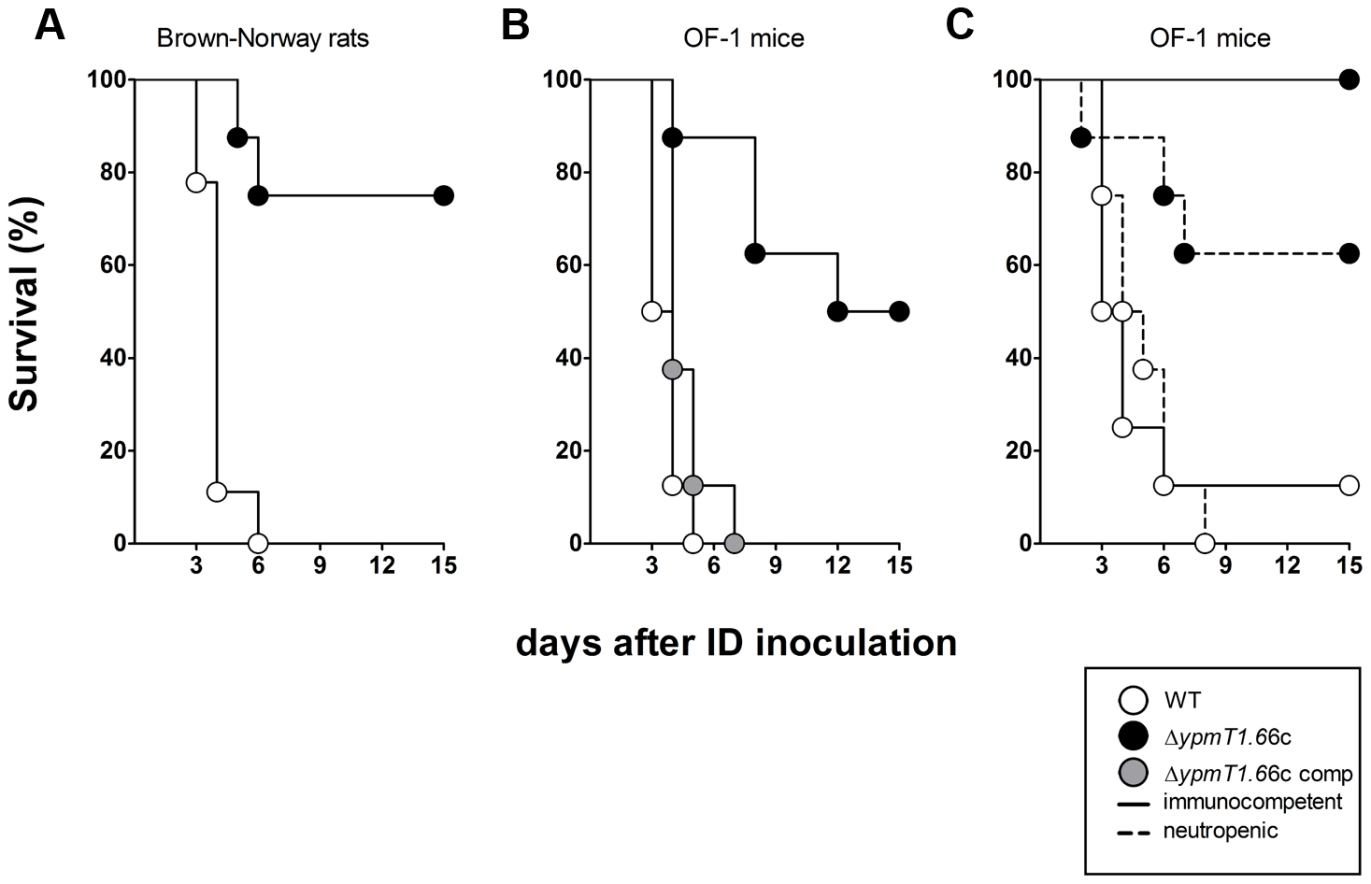
Incidence of plague in immunocompetent rats (A), immunocompetent mice (B), and neutropenic mice (C) injected intradermally with ∼20 WT *Y. pestis* (white circles), Δ*ypmt1.66c* (black circles) or complemented mutant (grey circles). The mutant was significantly (p<0.05) less virulent than the WT strain in immunocompetent rats (A), immunocompetent mice (B and C) and neutropenic mice (C). The survival curves of mice infected with the complemented mutant harboring the *ypmt1.66c* gene on a high copy number plasmid (pCR2) and the wild-type (B) were significantly different (p<0.05). The virulence of *Y. pestis* lacking *ympt1.66c* was no greater in neutropenic mice than it was in immunocompetent mice (dashed lines) (p>0.05). Data were obtained from groups of 8 or 9 animals. P values were determined using the Gehan-Breslow-Wilcoxon test.

**Figure 4:**
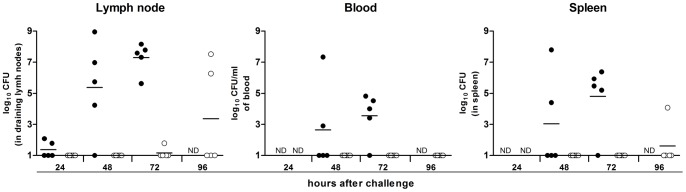
The change over time in colonization and bacterial load in rats inoculated intradermally with ∼20 WT *Y. pestis* (white circles) or Δ*ypmt*1.66c (black circles). N.D: not determined. Each circle represents one animal. The times to colonization were significantly longer in rats infected with the mutant than in rats infected with the WT strain (p<0.05). P values were determined using 2-way analysis of variance.

**Figure 5:**
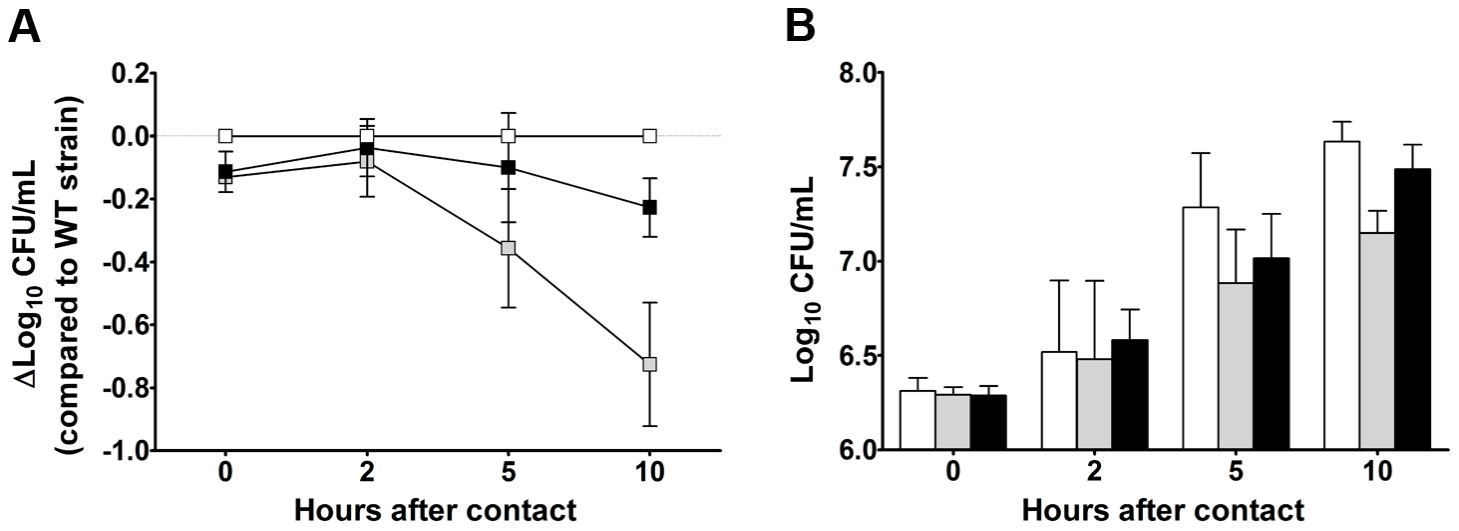
YPMT1.66c is required for intracellular survival in macrophages (A) and for optimal growth in serum (B). Shown are the data obtained with the wild-type (white squares and bars), the Δ*ypmt1.66c* (grey squares and bars) and the complemented mutant (black squares and bars) strains. Data are quoted as the mean (SD) from three independent experiments using macrophages (A) and 5 independent experiments using serum from 5 different healthy donors (B). The mutant's survival (A) and growth curves (B) differed significantly from those of the WT and the complemented strain (p<0.05 in a two-way analysis of variance). The complemented mutant's and the WT's survival (A) and growth curves (B) did not differ significantly (p>0.05 in a two-way analysis of variance).

Some of the *Y. pestis* genes overexpressed in the rat bubo and required for virulence in the present study have been acquired horizontally. These genes include the P4-like prophage genes (*ypo1089* to *ypo1091*) and the *ypmt1.66c* gene (which encodes a putative DNA binding protein and is located on the pMT1 plasmid). A Δ*ypmt1.66c* mutant was outcompeted in a per-pool assay (Table S4). Furthermore, the absence of the *ypmt1.66c* gene decreased the incidence of plague in both rats and mice (Figure 3). The virulence of the mutant expressing a WT copy of *ypmt1.66c* under the control of its own promoter from a high copy number plasmid (pCR2) was almost identical to that of the wild-type strain. The complemented mutant killed 100% of animals but caused fatal plague with a slight lag (a median survival time of 4 days, vs. 3.5 for the WT). Even though this slight difference in virulence was statistically significant (P = 0.038), this result indicated that loss of virulence was certainly due to deletion of *ypmt1.66c*. Interestingly, animals which survived inoculation with the Δ*ypmt1.66c* mutant had enlarged, purulent lymph nodes but not severe splenomegaly - suggesting that the mutant was impaired in its ability to initiate skin colonization and/or to escape from the draining lymph node. Consistently, the times to colonization of the lymph node, blood and spleen were significantly longer in rats infected with the mutant than in rats infected with the WT strain (Figure 4). Taken as a whole, our data indicate that a mutant's virulence is attenuated because the host manages to contain the infection during the initial skin colonization step, for which intracellular replication within macrophages is thought to be important [6]. In accordance with this model, the Δ*ypmt*1.66c mutant was more susceptible to macrophages than the WT and the complemented mutant (Figure 5A). The mutant's failure to initiate infection at the fleabite site could also result from its inability to use available nutrients or to survive contact with PMNs once released from lysing macrophages. The mutant showed a slight but statistically significant growth defect in serum (used here as model of nutrient acquisition in the skin), when compared with the WT and the complemented strain (Figure 5B). Furthermore, the mutant's respective levels of virulence in neutropenic and immunocompetent mice did not differ significantly (Figure 3C); this indicates that YPMT1.66c is involved in a biological process situated upstream of interaction with PMNs. Taken as a whole, our data suggest that YPMT1.66c's role in survival within macrophages is important in the production of plague.

### Stress, metabolic and uncharacterized genes are required for resistance and/or growth in serum

**Figure 6:**
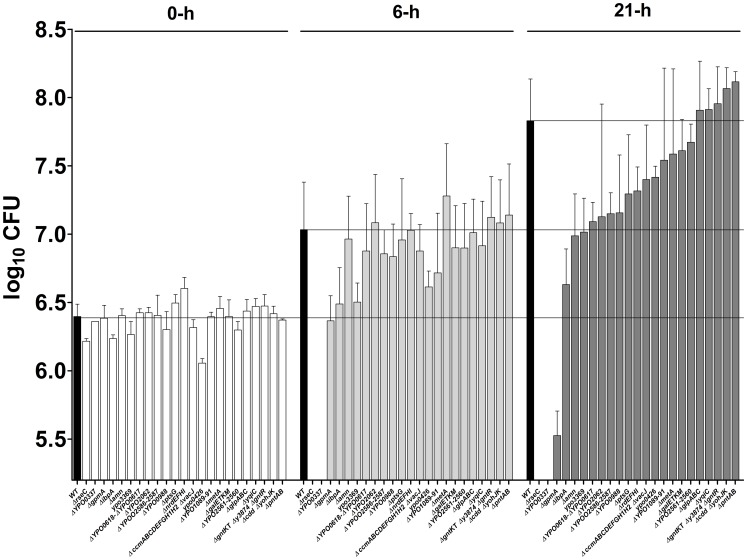
Stress, metabolic and uncharacterized genes are needed for resistance and/or growth in serum. The ability of selected mutants to grow in fresh serum was evaluated after 6 and 21Δ*rseC*, Δ*ypo0337*, Δ*gpmA*, Δ*ibpA*, Δ*ypo3369*, Δ*ypo0988*, Δ*amn*, Δ*ypo0617-0618*, Δ*ypo2586-2587* and Δ*ypo0426* mutants differed significantly from that of the wild-type strain (p<0.05 in a two-way analysis of variance).

Approximately 40% of the mutants found to be virulence-attenuated in individual tests and classified in the high-to-low likelihood class for virulence attenuation (i.e. ARCI≤10 and 10<ARCI<20) are known to encode proteins related to metabolic pathways. The remaining 60% of mutants lack factors considered to be involved in stress resistance (e.g. IbpA or RseC), bacterial attachment or serum resistance (e.g. Ail). Indeed, most of the remaining mutants are involved in hypothetical or uncharacterized processes. To determine whether the selected mutants were impaired for bacterial growth *in vivo* rather than for resistance to the host immune system, we measured their ability to grow in fresh serum (Figure 6). However, the mutants with a 10<ARCI<20 were not evaluated because we expected few strains in this ARCI category to be truly virulence-affected (see discussion) and therefore expected even fewer mutants to be serum-sensitive. Furthermore, the Δ*aceEF* mutant (identified in an individual virulence test) was not evaluated because its serious growth defect in LB led us to expect a growth defect in serum. Lastly, the ARCI≤10 mutants lacking genes that encode fimbriae or genes found to be non-essential for plague in individual tests were not evaluated because (i) we did not expect fimbriae to be involved in serum resistance and (ii) mutants that were found to be attenuated in per-pool screening but not in individual tests were considered to be false positives. Hence, a total of 23 mutants were evaluated for serum resistance. Ten of them differed significantly from the wild-type strain. In particular, the Δ*rseC*, Δ*ypo0337* and Δ*gpmA* mutants were sensitive to serum (presumably because of the bactericidal effect of the complement), and the Δ*ibpA*, Δ*ypo3369*, Δ*ypo0988*, Δ*amn*, Δ*ypo0617-0618*, Δ*ypo2586-2587* and Δ*ypo0426* mutants had significant a growth defect (either because they confer a metabolic advantage or effective complement tolerance).

## Discussion

Here, we presented a novel experimental approach for establishing a hierarchical table of *Y. pestis* genes previously identified (in a microarray analysis) as being up-regulated *in vivo*. The genes' ranking in terms of their importance in virulence should provide new insights into the pathogenesis of plague. Our approach consisted of (i) testing the virulence of individual mutants each lacking a locus likely to encode factors important for plague pathogenesis and (ii) per-pool screening of a library of mutants composed of mutants strains each lacking a locus with no obvious role in pathogenesis and of which ∼60% of the strains lacked genes that were not directly involved in any known metabolic pathway. Our per-pool screening method is based on signature-tagged mutagenesis (STM) [40] and is closely related to “array-based analysis of cistron under selection” (ABACUS) technology [41]. We first generated a library of non-polar, tagged deletion mutants by using lambda-Red technology [13] (as in ABACUS) rather than a transposon mutant library (as in STM). Each mutant in the pool was tagged with a unique antibiotic resistance cassette, which ultimately allowed the numbers of viable, tagged mutants in infected tissues to be quantified by CFU counting on selective media. This aspect contrasts with the ABACUS method, in which (i) a T7 RNA polymerase promoter is inserted into the target gene and (ii) mutants are monitored with a microarray using labeled RNAs synthesized *in vitro* from the T7 promoter and mutant DNA that has been fragmented, poly(A)-tailed and amplified prior to transcription. Hence, in contrast to ABACUS and other methodologies based on microarrays or deep sequencing (such as STM-like or transposon-directed insertion-site sequencing), our method does not require nucleic acid purification, RNA synthesis, PCRs, microarray hybridization or sequencing [41], [42], [43]. It therefore minimizes the likelihood of introducing bias related to sample preparation, hybridization and/or sequencing. One could argue that our “low complexity”, per-pool approach is limited by the number of antibiotic tags available. However, various fluorescent tags (such as green or red fluorescent protein), bioluminescent tags (blue or red) and enzymatic tags (such as LacZ) can be used to increase the number of mutants in the pool. Hence, it would be easy to match the pool to the desired complexity and, ultimately, to the most relevant infection conditions. In fact, the tag approach enabled us to generate a low-complexity pool that corresponded to inoculation of a physiological dose of bacteria, i.e. that delivered by a flea during a blood meal.

By injecting pools of mutants under the most relevant infection conditions, we confirmed previous suggestions whereby *Y. pestis* encounters a bottleneck during dissemination from the skin (Figure S4) [20], [44], [45]. This dissemination bottleneck has been observed for several pathogens (including *Bacillus anthracis* and *Francisella tularensis*) and was previously considered to prevent the use of per-pool screening methodologies other than when very high doses were inoculated [25], [41]. Our results do not support the latter hypothesis. Our calculation of the ARCI enabled us to address the bottleneck problem and thus draw up a hierarchical table of *Y. pestis* genes in terms of their importance in plague. We then validated this hierarchical table by testing the virulence of several mutants individually. With the exclusion of *ail* (which was identified as needed for plague pathogenesis by other researchers while we were performing our experiments [46]), 16 new loci were found here to be important for virulence in mice and/or rats (Tables S3 and S4). Among them, the uncharacterized *ypmt1.66c* and *ypo0988* genes were required for optimal growth in serum (Figures 5B and 6). Furthermore, the uncharacterized *ypo0337* gene (previously identified by signature-tagged mutagenesis in *Yersinia pseudotuberculosis* [45], the recent ancestor of *Y. pestis* [47]) was essential for serum resistance (Figure 6); this finding suggested that YPO0337 is a new, uncharacterized virulence factor. Thirty-two mutants identified as being virulence-attenuated in per-pool screening were not reassessed individually in the present study (Table S4). Eleven and 20 of these mutants belonged to the ARCI≤10 and ARCI<20 classes respectively. Based on the virulence of individual mutants in the different ARCI classes, we estimate that 65% of the mutants with an ARCI<10 and 20% of those with an ARCI<20 might truly be virulence-attenuated. Hence, at least 12 other loci can conceivably be added to the current list of 16 new loci found to be important in plague pathogenesis. Accordingly, several genes were required for survival or optimal growth in serum (Figure 6). However, we cannot rule out the possibility that some of the mutants identified here are defective in virulence because they harbor a secondary mutation (Figure S5). The apparent absence of complementation might also result from use of an inadequate method or other, uncharacterized features.

Our virulence testing results suggest that in the mammalian host, *Y. pestis* (i) relies on carbohydrates (i.e. glucose, galactans, gluconate and perhaps maltose) as its carbon source and (ii) shifts to anaerobic respiration (maybe using dimethyl sulfoxide (DMSO) or glycerol) and/or fermentation (Figure 2 and Tables S4, S5, S6). The data indicated that glucose (imported via the PtsG transporter) and galactans were channeled towards the synthesis of UDP-glucose rather than to glycolysis. Firstly, *Y. pestis* does not encode a functional glucose-6-P dehydrogenase (Zwf, the enzyme that catalyzes the first step in the metabolism of hexose *via* the pentose phosphate pathway [48]. Secondly, deletion of the first two enzymes in the glycolysis pathway (Pgi and PfkA) did not impact virulence (Figure 2 and Table S3). Whereas carbohydrates do not feed into the upper part of glycolysis, gluconate (taken by the GntM transporter) can potentially supply glyceraldehyde-3-phosphate (G3P) to the lower part of the pathway. The latter metabolite appeared to be essential for plague production, since a *gpmA* mutant was completely outcompeted *in vivo* (Figure 2 and Table S4). Glyceraldehyde-3-phosphate is an important carbon source in hexose and amino-acid anabolism and pyruvate production [49]. However, we do not have any evidence to suggest that G3P is involved in hexose and amino-acids anabolism in *Y. pestis*. In particular, SerA (YPO0914, which catalyzes the first step in the biosynthesis of serine from glycerate-3P that is produced from G3P) was not necessary for plague. Whereas G3P's role in hexose and amino-acid anabolism in *Y. pestis* has yet to be confirmed, this compound is likely to be crucial for the production of pyruvate *in vivo* because a mutant lacking the pyruvate dehydrogenase AceEF was unable to produce fatal plague when injected at a dose known to kill most animals. Lastly, the facts that (i) the tricarboxylic acid cycle (TCA) is down-regulated *in vivo* [7], (ii) the mutant lacking the acotinase A (part of the TCA cycle) is fully virulent (unpublished data) and (iii) the mutant lacking the heat-stable iron-independent fumarate enzyme (FumC, part of the TCA cycle) was not outcompeted in per-pool virulence screening in contrast to the mutants lacking DMSO reductase (DmsABC, YPO2965-2969) and glycerol-3P dehydrogenase (GlpABC) – both involved in anaerobic respiration - suggested that *Y. pestis* relies on anaerobic respiration to produce plague.

Our virulence testing results also suggest that in the mammalian host, *Y. pestis* experiences iron, RNS and temperature stresses but not ROS stress (Figure 2 and Tables S4, S5, S6). This supports the hypothesizes generated on the basis of *in vivo* gene expression profiling [7]. However, we tested a small number of genes involved in bacterial resistance against ROS (Table S6) because few oxidative stress response genes are activated in the rat bubo [7]. Several of the genes involved in the detoxification of ROS which are not upregulated or even downregulated *in vivo* are upregulated in *Y. pestis* replicating inside macrophage-cell line [34] - a step suggested to be important in the initial stages of infection [6]. The difference in expression levels found in rat bubo and in macrophages cell-line *in vitro* may indicate that *Y. pestis* encounters ROS during the initial stage of infection but not later on. However, the detoxifying hydroperoxidase *katG* gene (the expression of which is upregulated ∼10 fold during replication within the macrophage) was found to be dispensable for plague pathogenesis [50]. Hence, the role of ROS during plague infection remains to be accurately established by systematic deletion of the ROS resistance/responding genes and characterization of the resulting mutants.

Our screening results also suggested that *Y. pestis* only needs a limited number of genes to successfully infect its mammalian host. This might be attributable to redundant genes that constitute a “spare wheel” system. For instance, serine biosynthesis enzyme SerA (YPO0914) was not required for virulence but its loss could be rescued by a second putative SerA (YPO1288) enzyme. Alternatively, the requirement for a limited number of genes may be because only one of several genes with overlapping functions are needed in a given context. For example, *Y. pestis* upregulates both *idnK* and *gntV* encoding gluconate kinases but only *gntV* was required for virulence (Figure 2) [51]. One can hypothesize that IndK is needed to establish an infection in the flea. Lastly, the requirement for just a limited number of genes might indicate ancestral needs for colonizing other environments. Hence, one would expect at least some of the redundant genes to be inactivated and/or deleted from the genome as the bacterium retains only the most appropriate system for its current lifestyle. This is consistent with the suggestion that *Y. pestis* is undergoing genome decay [52]. Data on the role of iron acquisition systems in plague provide support for this decay scenario. Of the four siderophore-based uptake systems overexpressed by *Y. pestis in vivo* (yersiniabactin, pseudochelin, aerobactin and alcaligin) and whose homologs in other bacteria are known to have a role in lifestyle, only the *Yersiniabactin* system is crucial for plague - presumably because the aerobactin and alcaligin systems are not functional in *Y. pestis* (Table S5) [7], [26], [53], [54].

In addition to genome decay, the emergence of *Y. pestis* has been characterized by the horizontal acquisition of the plasmids pPst and pMT1/pFra [52]. The two plasmids have had a crucial role in the emergence of flea-borne plague [55], [56], [57], [58], [59], [60], [61]. pPst encodes a plasminogen activator that is thought to prevent the host from containing the *Y. pestis* infection at the dermal fleabite site. pFra encodes the pseudocapsule F1, whose antiphagocytic activity and/or ability to interfere with interleukin-1 signaling increases the incidence of plague. pFra harbors also several uncharacterized regions (located near the *caf* operon and elsewhere) which may encode virulence factors [62]. None of these regions contained the uncharacterized gene *ypmt1.66c* (encoding a protein that is conserved among *Y. pestis* strains). Here, we found that that *ypmt1.66c* is also important for maximum disease production at the dermal infection site (Figures 3 and 4). Our observation provides a new example of the importance of horizontal gene acquisition in the emergence of flea-borne plague.

The deletion of *ypmt1.66c* reduced *Y. pestis*' ability to resist macrophages, and slightly hampers the bacterium's ability to grow in normal serum (Figure 5) - suggesting that *ympt1.66c* is partially needed for resisting complement and/or to acquiring nutrients from the serum (and perhaps also at the fleabite site). We did not test whether YPMT1.66c is needed to confront the PMNs that are recruited *en masse* in infected tissues. Even if this is the case, YPMT1.66c's role as an intracellular survival factor appears to be a prerequisite for resistance to PMNs because the virulence of a mutant lacking YPMT1.66c was not restored in neutropenic mice (Figure 3C). Taken as a whole, our data provide support for the scenario in which *Y. pestis* regurgitated at the fleabite site is taken up by macrophages and indicate that YPMT166c is required for replication within the phagocyte. This scenario is consistent with the current model of plague, in which the conditions for intracellular replication at the initial stage of infection (i.e. at the dermal flea bite site) are critical for the subsequent production of a bubonic infection [6], [9].

### Conclusion

We leveraged our previously collected *in vivo* gene expression profiling data on *Y. pestis* [7] to generate and screen a non-polar tagged deletion mutant library composed of bacteria lacking genes known to be upregulated *in vivo*. This approach constituted the first integrated attempt to identify the molecular mechanisms of the pathogenesis of plague in the most relevant biological model of infection - the mammalian host. Our approach could be transposed to other pathogens and notably those for which a dissemination bottleneck has been described [24], [25]. Our data provided further insights into the pathogenesis of plague by identifying new genes important for plague pathogenesis (including several previously uncharacterized sequences). The fact that these genes are shared with several other pathogens suggests that they belong to a core set of bacterial pathogenesis genes. Confirmation of these virulence results (by individually testing the virulence of mutants selected from the pools and by performing complementation experiments) and further characterization of these new identified genes will be necessary. Future studies should therefore improve our understanding of pathogenesis and, in particular, yield putative antibiotic targets for several diseases.

## Supporting Information

Figure S1 Equivalent incidences of plague in Brown-Norway rats inoculated intradermally with either ∼10 *Y. pestis* CO92 or ∼10 *Y. pestis* CO92 mini-Tn*7*T*::Zeo*1 (*p*>.045 in a Gehan-Breslow-Wilcoxon test). Data were obtained from one experiment on a group of 8 animals.
(TIF)

Figure S2 Incidence of plague in Brown-Norway rats inoculated intradermally with an input pool of five different *Y. pestis* mutants (20 CFUs per mutant and 100 CFUs in total) (A–F). Groups of 10 animals were infected with each pool and four groups were infected on the same day.
(TIF)

Figure S3 Bacterial loads in (A) the lymph nodes draining the inoculation site and (B) the spleens from the rats used in the per-pool screening. Rats were inoculated intradermally with an inoculum comprising five mutants (20 CFUs per mutant and 100 CFUs in total). The bacterial loads recovered from the lymph nodes and the spleens from the various groups of rats did not differ significantly (p<0.05 in a one-way analysis of variance with Bonferroni correction for multiple comparisons).
(TIF)

Figure S4 A bottleneck affects the dissemination of *Y. pestis* from the intradermal inoculation site. The figure shows the bacterial loads recovered from the lymph nodes and the spleens from a group of 10 rats (R1 to R10) inoculated intradermally with five mutants (20 CFUs per mutant and 100 CFUs in total). Typically, each animal was predominantly colonized by just one of the pool's mutants. Red symbols indicate the predominant mutant strain. Grey symbols indicate mutants with at least 30% of the CFUs of the predominant strain.
(TIF)

Figure S5 Incidence of plague in mice infected with *Y. pestis* lacking selected uncharacterized genes. (A–F) Animals were injected intradermally with ∼10 CFU of *Y. pestis* WT (white circles), the mutant lacking the gene of interest (white squares or white triangles) or the mutant harboring a wild-type copy of the gene of interest (grey squares) under the control of a pAra promoter (A) or its own putative promoter (B–F). All six mutants were significantly affected in virulence compared to the wild-type strain (p<0.05). In contrast to the complemented Δ*ypo3369*, Δ*ypo2560 -2561* and Δ*ypo3991* mutants, the virulence levels of the Δ*ypo0988*, Δ*ypo2062* and Δ*ypo0656* mutants harboring a wild-type copy of the gene of interest (“c”) differed significantly (p<0.05) from that of the original mutant strain but were similar to that of the wild-type strain. . Groups of eight animals were used.
(TIF)

Table S1 Strains and plasmids.
(PDF)

Table S2 Primer sets used in the study.
(PDF)

Table S3 Virulence of *Y. pestis* mutants tested individually (due to prioritization or technical issues).
(PDF)

Table S4 The role of *Y. pestis* genes in plague, on the basis of per-pool screening.
(PDF)

Table S5 Role of metal acquisition systems in plague.
(PDF)

Table S6 The role of some *Y. pestis* genes predicted to protect against RNS and ROS *in vivo*.
(PDF)

Text S1 References for supplementary tables.
(DOCX)
