# Supplementary figures and images for: New Insights into How Yersinia pestis Adapts to Its Mammalian Host during Bubonic Plague
Source: PLoS Pathog. 2014 Mar 27;10(3):e1004029. doi: 10.1371/journal.ppat.1004029 (PMC3968184; doi:10.1371/journal.ppat.1004029)

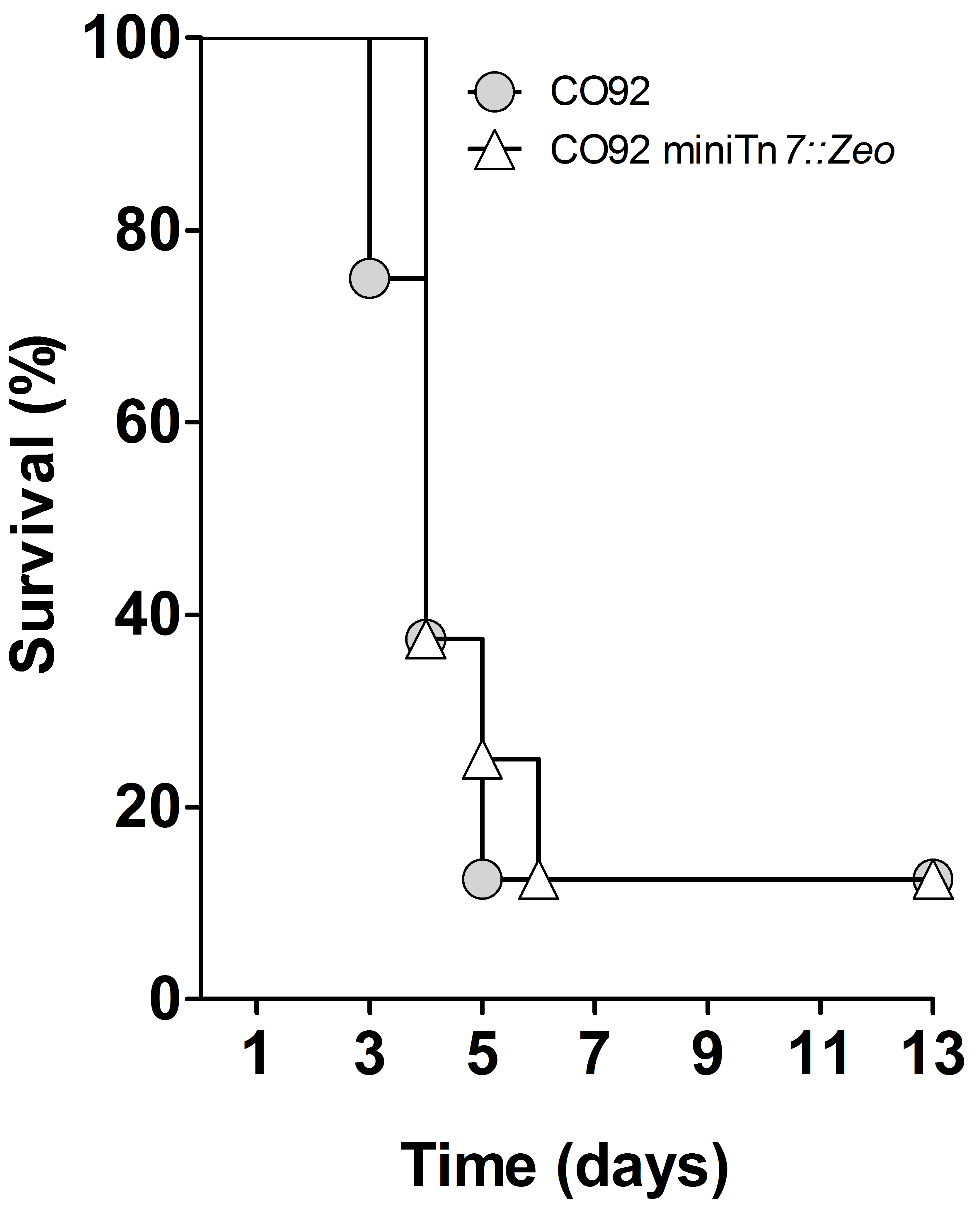

Supplement: Figure S1 — Equivalent incidences of plague in Brown-Norway rats inoculated intradermally with either ∼10 Y. pestis CO92 or ∼10 Y. pestis CO92 mini-Tn7T::Zeo1 (p>.045 in a Gehan-Breslow-Wilcoxon test). Data were obtained from one experiment on a group of 8 animals. (TIF) [file ppat.1004029.s001.tif]

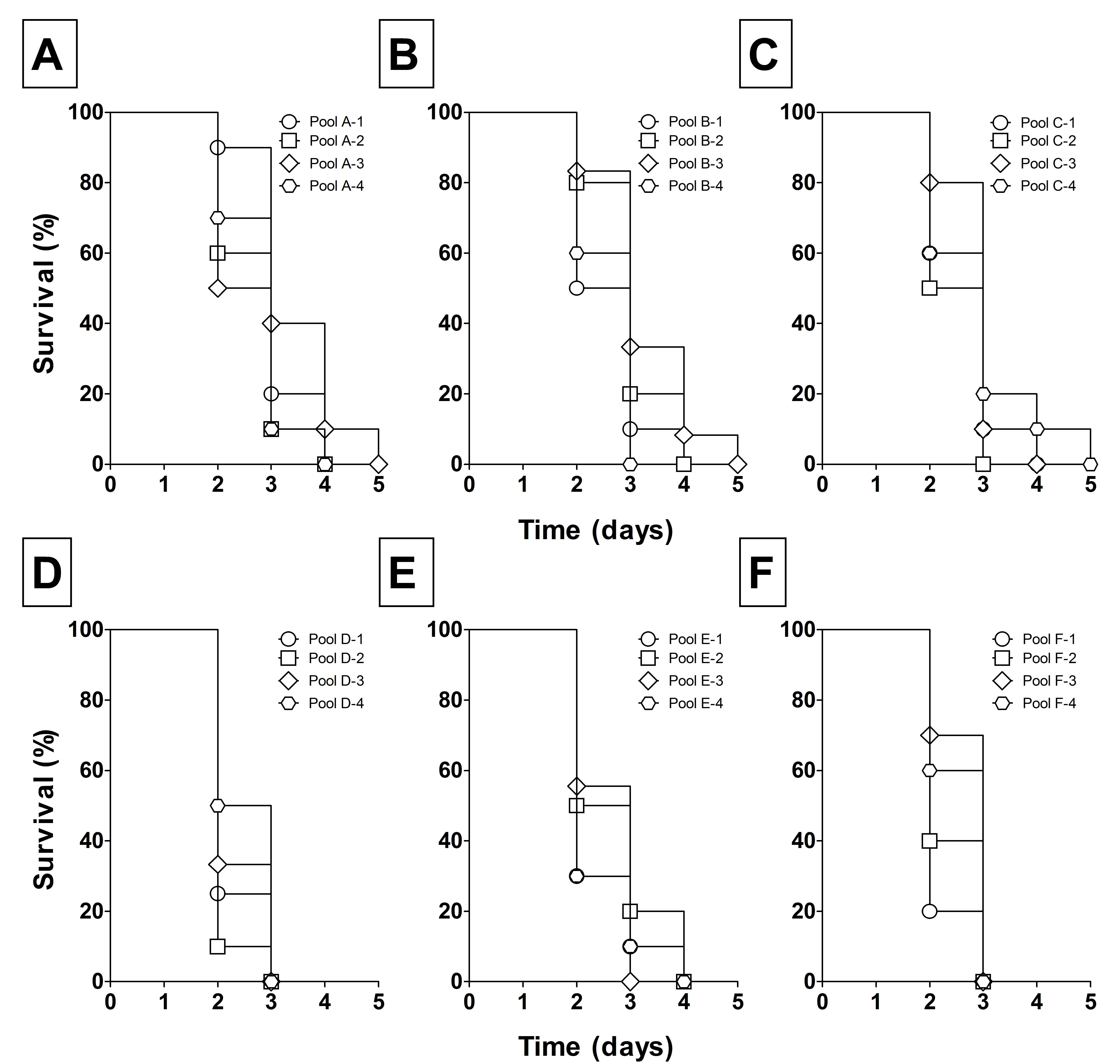

Supplement: Figure S2 — Incidence of plague in Brown-Norway rats inoculated intradermally with an input pool of five different Y. pestis mutants (20 CFUs per mutant and 100 CFUs in total) (A–F). Groups of 10 animals were infected with each pool and four groups were infected on the same day. (TIF) [file ppat.1004029.s002.tif]

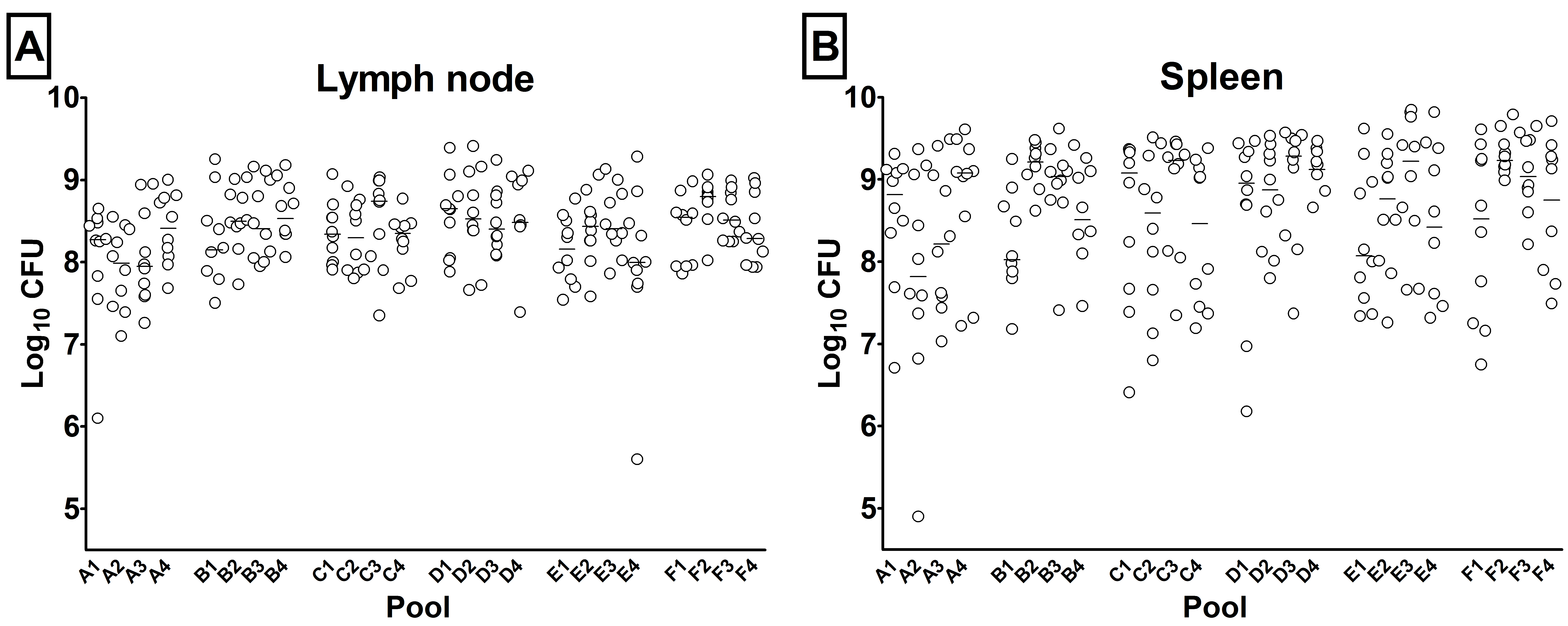

Supplement: Figure S3 — Bacterial loads in (A) the lymph nodes draining the inoculation site and (B) the spleens from the rats used in the per-pool screening. Rats were inoculated intradermally with an inoculum comprising five mutants (20 CFUs per mutant and 100 CFUs in total). The bacterial loads recovered from the lymph nodes and the spleens from the various groups of rats did not differ significantly (p<0.05 in a one-way analysis of variance with Bonferroni correction for multiple comparisons). (TIF) [file ppat.1004029.s003.tif]

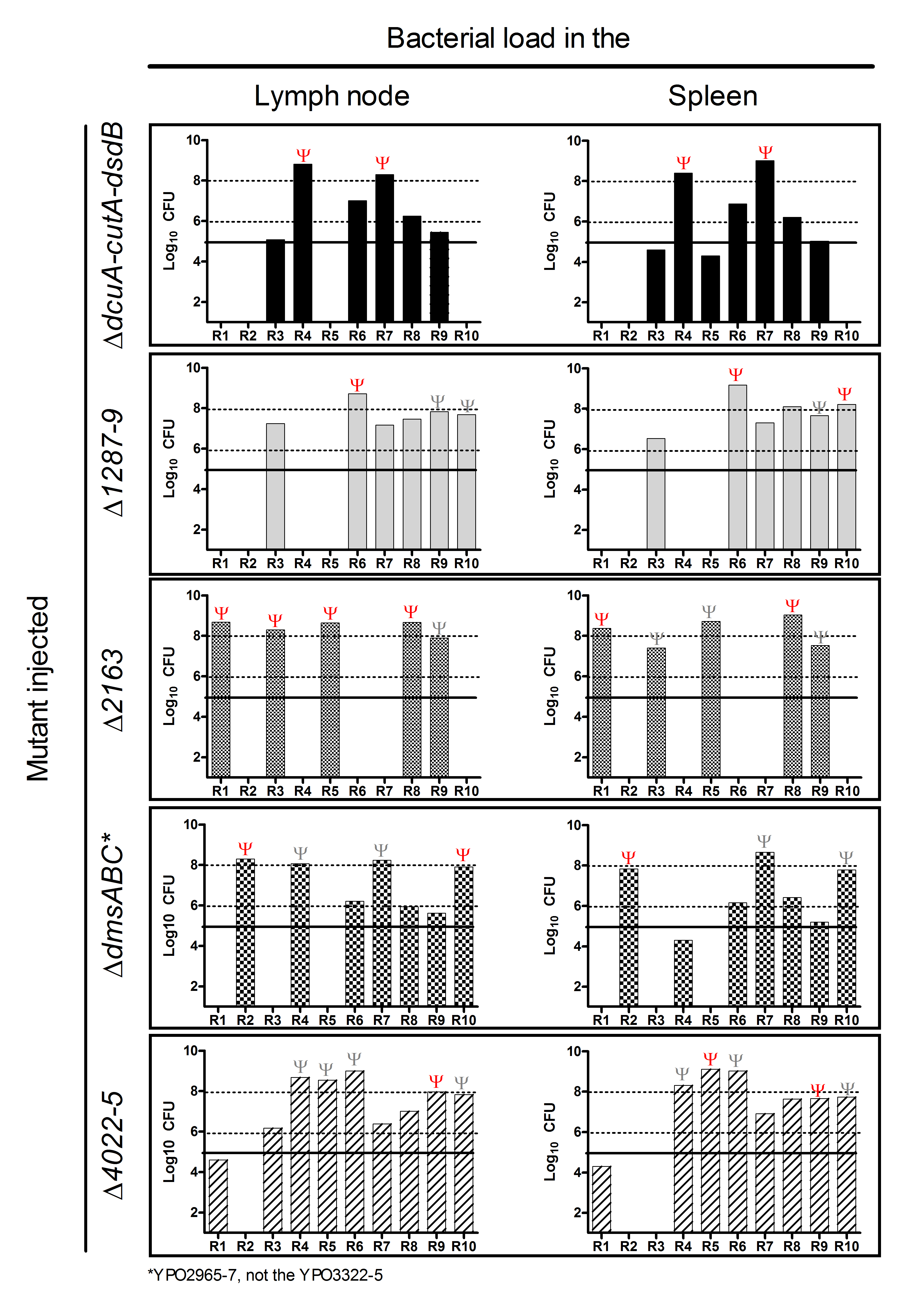

Supplement: Figure S4 — A bottleneck affects the dissemination of Y. pestis from the intradermal inoculation site. The figure shows the bacterial loads recovered from the lymph nodes and the spleens from a group of 10 rats (R1 to R10) inoculated intradermally with five mutants (20 CFUs per mutant and 100 CFUs in total). Typically, each animal was predominantly colonized by just one of the pool's mutants. Red symbols indicate the predominant mutant strain. Grey symbols indicate mutants with at least 30% of the CFUs of the predominant strain. (TIF) [file ppat.1004029.s004.tif]
