# Supplementary material for: New Insights into How Yersinia pestis Adapts to Its Mammalian Host during Bubonic Plague
Source: PLoS Pathog. 2014 Mar 27;10(3):e1004029. doi: 10.1371/journal.ppat.1004029 (PMC3968184; doi:10.1371/journal.ppat.1004029)
Supplement: Table S1 — Strains and plasmids. (PDF) [file ppat.1004029.s006.pdf]

Table S1. Strains and plasmids

Strains or plasmids	Relevant characteristics[a]	Reference or origin[b]
Strains		
Yersinia pestis [c]		
CO92	Wild-type strain	[1]
CO92[ZR]	Derived from strain CO92, mini Tn*7* ::*Sh ble;* Zeo	This work
195/P	Wild-type strain	[2]
Escherichia coli		
DH5α	*supE* , Δ*lac* U169 (ϕ 80 *lac* ZΔM15) , *hsdR* , *recA* , *endA* , *gyrA* , *thi* , *relA*	Invitrogen
DH5α (*λpir*)	*λpir* enables oriR6K vectors replication	
S17-1 (λpir)	RP4-2-Tc::Mu-Km::Tn*7/pro hsd* R; host for pCVD442 and derivates	[3]
Plasmids		
pCRII	Cloning vector; Ap and Km	Invitrogen
pUC18	Cloning vector; Ap	[4]
pBAD30	Cloning vector; Ap	Invitrogen
pUC4K	Source of Km cassette for production of mutant using pCDV442 technology, Km	[5]
pCVD442	Suicide vector containing the counter selectable marker *sacB* ; Ap	[6]
pKD4	oriR6K vector, source of Km cassette	[7]
pKD46	vector bearing Red recombinase, Ap	[7]
pEP1087	derived from pKD4, source of Tp cassette	This work (GenBank accession number KF771027)
pEP1042	derived from pKD4, source of Zeo cassette	This work (GenBank accession number KF771026)
pEP1013	pKD46 bearing *sacB* , ApR	This work (GenBank accession number KF771025)
pEP1151	pCRII containing *ypmt1.66c* under the control of its putative promoter	This work
pEPypo0656	pCRII containing *ypo0656* under the control of its putative promoter	This work
pEPypo2062	pCRII containing *ypo2062* under the control of its putative promoter	This work
pEPypo3369	pCRII containing *ypo3369* under the control of its putative promoter	This work
pEPypo25601	pCRII containing *ypo2560-2561* under the control of its putative promoter	This work
pEPypo3991	pCRII containing *ypo3991* under the control of its putative promoter	This work
pEPypo0988	pBAD30Ω, *Sac* I/*Xba* I insert encompassing the *ypo0988* coding sequence	This work
pUC18R6K-mini-Tn7T	oriR6K vector bearing empty mini-Tn7T, ApR	[8]
pTNS2	oriR6K vector, helper for mini-Tn7 transposition, ApR	[8]
pEP1045	source of mini-Tn7T::Zeo1, derived from pUC18R6K-mini-Tn7T	This work
pNL1	pUC18W, ~0,5-kb *Sma* I/*Sac* I insert with primer set N1-N2 encompassing the upstream region of *metF* gene from *Y. pestis*	
pNL2	pNL1Ω, ~1,1-kb *Sma* I/*Xba* I insert with primer set N5-N6 encompassing *aphA* from pUC4K	This work
pNL3	pNL2Ω, ~0,6-kb *Xba* I/*Pst* I insert with primer set N3-N4 encompassing the downstream region of *metF* gene from *Y. pestis*	This work
pNLΔ*metF*	pCVD442Ω, ~2,2-kb *Sac* I insert from pNL3	This work

Strains or plasmids	Relevant characteristics[a]	Reference or origin[b]
pNL4	pUC18Ω ~0,5-kb *Sma* I/*Sac* I insert with primer set N7-N8 encompassing the upstream region of *metE* gene from *Y. pestis*	This work
pNL5	pNL4Ω, ~1,1-kb *Sma* I/*Xba* I insert with primer set N11-N12 encompassing *aphA* from pUC4K	This work
pNL6	pNL5Ω, ~0,5-kb *Xba* I/*Pst* I insert with primer set N9-N10 encompassing the downstream region of *metE* gene from *Y. pestis*	This work
pNLΔ*metE*	pCVD442Ω, ~2,2-kb *Sac* I insert from pNL6	This work
pNL7	pUC18Ω, ~0,5-kb *Hind* III/*Pst* I insert with primer set N13-N14 encompassing the upstream region of *fhuF* gene from *Y. pestis*	This work
pNL8	pNL7Ω, ~1,1-kb *Pst* I/*Sma* I insert with primer set N17-N18 encompassing *aphA* from pUC4K	This work
pNL9	pNL8Ω, ~0,5-kb *Sma* /*Sac* I insert with primer set N15-N16 encompassing the downstream region of *fhuF* gene from *Y. pestis*	This work
pNLΔ*fhuF*	pCVD442Ω, ~2,2-kb *Xba* I/*Sac* I insert from pNL9	This work
pNL10	pUC18Ω, ~0,5-kb *Pst* I/*Xba* I insert with primer set N19-N20 encompassing the upstream region of *nrdHIEF* gene from *Y. pestis*	This work
pNL11	pNL10Ω, ~1,1-kb *Xba* I/*Sma* I insert with primer set N23-N24 encompassing *aphA* from pUC4K	This work
pNL12	pNL11Ω, ~0,5-kb *Sma* /*Eco* RI insert with primer set N21-N22 encompassing the downstream region of *fhuF* gene from *Y. pestis*	This work
pNLΔ*nrdHIEF*	pCVD442Ω, ~2,2-kb *Sac* I insert from pNL12	This work
pNL13	pUC18W, ~0,5-kb *Sac* I/*Sma* I insert with primer set N25-N26 encompassing the upstream region of *yfiD* gene from *Y. pestis*	This work
pNL14	pNL13W, ~1,1-kb *Sma* I/*Pst* I insert with primer set N29-N30 encompassing *aphA* from pUC4K	This work
pNL15	pNL14W, ~0,5-kb *Pst* I/*Hind* III insert with primer set N27-N28 encompassing the downstream region of *fhuF* gene from *Y. pestis*	This work
pNLD*yfiD*	pCVD442W, ~2,2-kb *Sac* I/*Xba* I insert from pNL15	This work
pNL16	pUC18W, ~0,5-kb *EcoR* I/*Xba* I insert with primer set N31-N32 encompassing the upstream region of *mntH* gene from *Y. pestis*	This work
pNL17	pNL16W, ~1,1-kb *Xba* I/*Pst* I insert with primer set N34-N35 encompassing *aphA* from pUC4K	This work
pNL18	pNL17W, ~0,5-kb *PstI* /*Hind* III insert with primer set N33-N34 encompassing the downstream region of *mntH* gene from *Y. pestis*	This work
pNLD*mntH*	pCVD442W, 2,2-kb *Xba* I/*Sac* I insert from pNL18	This work
pNL19	pUC18W, ~0,5-kb *Pst* I/*Hind* III insert with primer set N40-N41 encompassing the downstream region of *asnA* gene from Y. pestis	This work
pNL20	pNL19W, ~1,1-kb *Pst* I/*Sma* I insert with primer set N38-N39 encompassing *aphA* from pUC4K	This work
pNL21	pNL20W, ~0,5-kb *SmaI* /*Sac* I insert with primer set N36-N37 encompassing the upstream region of *asnA* gene from *Y. pestis*	This work
pNLΔ*asnA*	pCVD442Ω, 2,2-kb *Xba* I/*Sac* I insert from pNL21	This work

[a], Ap, Km, Tp, Zeo, resistance to ampicilin, kanamycin, trimethoprim, zeocin respectively; suc, sensitivity to sucrose

[b], references are provided in text S1

[c], CO92 and 195/P are two Orientalis biovar strains. They show extremely low level of genetic diversity [9]
