# Supplementary material for: New Insights into How Yersinia pestis Adapts to Its Mammalian Host during Bubonic Plague
Source: PLoS Pathog. 2014 Mar 27;10(3):e1004029. doi: 10.1371/journal.ppat.1004029 (PMC3968184; doi:10.1371/journal.ppat.1004029)
Supplement: Table S2 — Primer sets used in the study. (PDF) [file ppat.1004029.s007.pdf]

Table S2. Primer sets used in the study

		ORF(s)	GENE(s)[†]	PRIMER SETS (5' -> 3') USED TO		MUTANT GENERATED IN[b]
				GENERATE THE MUTATION[a]	VERIFY THE MUTATION	
Mutants tested individually	Genes upregulated in rat bubo and mouse lung [a]	YPO0116	*metL*	ATGAATGCAACAGCGGTAGCAGCGGCGGCAACGGGCCGTCAACTGCATAAGTGTAGGCTGGAGCTGCTTC<br>TTACAACAGTTGTGATAGGCGGTTTAAATCAGATTGAATCGCCCCAGCGGCATATGAATATCCTCCTTAG	GCCACGAAATAGCCTGCCGT<br>AGATTGAATCGCCCCAGCG	CO92
		YPO0158-YPO0161	*nirBDC-cysG*	ATGAGCAAAATAAAAATTGCCATCATTGGCAACGGCATGGTCGGCCACCGGTGTAGGCTGGAGCTGCTTC<br>TTAACCAACATGAGCCACACATTCAGTCACTTTGGGCTGATCATCGTGATCATATGAATATCCTCCTTAG	ACCGTCGCCTGCCCGATTTT<br>CGCGCACAAGTAACCTTGGCT	CO92
		YPO0205- YPO206	*bfd-bfr*	ATGTACGTTTGTCTATGTAATGCGGTTTCTGACAAAGTCATTCGCAAAGGGTGTAGGCTGGAGCTGCTTC<br>CTAGTCTTTTGCTAGCTGGGCCTGTGAGTAGCTTTGTATACCTAAACGGTCATATGAATATCCTCCTTAG	CGTATCCCGGTAGCCCGTTT<br>GGCCCGCGCATTATACGTAA	CO92
		YPO0279-YPO0286	(-) & *hmuRSTUV*	ATGAATATTGATCTGATGCCGTATTATGCCCAACCAGGGCCACTGCCATTGTGTAGGCTGGAGCTGCTTC<br>TTATTGCCGCAGATAAATCTGTGGCAGGGCGCTTTCTGGGTGACGCGAAACATATGAATATCCTCCTTAG	TCACTCTGATGGGTGGGGCT<br>TGCTTCAGTTTTGCGGGTGA	CO92
		YPO0342-YPO344	(-) & *hydN-fdhD*	ATGAATCCATTTATTGTTGCTGACGCTGAGAGCTGTATTGGTTGTCGGAGGTGTAGGCTGGAGCTGCTTC<br>TCATGGTACAACTGCGGCGTCTGATAACCGTTTTTTCAACTGGCTGTATTCATATGAATATCCTCCTTAG	ACCCCGTTGTCGGTTGACGT<br>GCCAACAATGCTGAACATGCC	CO92
		YPO0426	(-)	TTGCTCAGCCGTGGCATGTCCAGTGGCTGGGGAATATTGCTGCCTTTTACGTGTAGGCTGGAGCTGCTTC<br>TCATGACACACCACCGTGCATTAGCATTAGCGCGACCAAGCCGCCTAACACATATGAATATCCTCCTTAG	CATGATGTCGTTCAACGGCG<br>CGTCTACCAATTCCGCCACC	CO92
		YPO0988	(-)	ATGCGCATATCCTCAGAGTCTGACGCTCGCTCAAGCGGGCTTACATCGTCGTGTAGGCTGGAGCTGCTTC<br>TTACTCTCCCACAGAATGGGCGGTTGTTCGATGCTGGCACCACAGCCAGCCATATGAATATCCTCCTTAG	TGGGTTTCACGAATCAGCGC<br>ACACCTGCTCATCATCGGGC	CO92
		YPO1008-YPO1012	(-) *ynp1 ynp2*	ATGTCAGATGTAAAACCGCAGAACGATCGGCAGACCGGCATACTGAACGCGTGTAGGCTGGAGCTGCTTC<br>CTAATGCAAATCCCCCCGCAGCGGCCTCCAAGTGCCCAACCTGACTTTTACATATGAATATCCTCCTTAG	GGGATGAGTAAGGGCAGCCA<br>GGGGTGACCAAAGGCCTTCT	CO92
		YPO1310-1313	*yiuABCR*	GTGAAATGTGTTTCACTGAAAATGGCTAAAGGGTTAACTATCAGTTTGTTGTGTAGGCTGGAGCTGCTTC<br>TTAAAAATCGTAGCTGGCACCCACCATATACCGACGACCATCCAACACCTCATATGAATATCCTCCTTAG	TGCGGATGAAAGGGCGAT<br>CACCTTGGCATTGCTGGTCA	CO92
		YPO1386	*ansB*	ATGAAATATATAAAGCTAACGGTACTCGCCGGAATATTTGTAGGGATCAGGTGTAGGCTGGAGCTGCTTC<br>TCAATATGTGTGGAATAGTTTTTGAATTTCTTGTGGTTTTTGAGTTTGCGCATATGAATATCCTCCTTAG	CTGATGCCAGAGCCGCTTTT<br>GGGGCGTCCATTCCATTACAA	CO92
		YPO1516-1517	(-)	ATGAAAAAAATCGCTTACCCTATATTGGCTTTGAGCCTACTCACAGCACTGTGTAGGCTGGAGCTGCTTC<br>TTAACCTAAATATTCGATGATAGAGAGGCCGCCGTTATAATCGGTACTGTCATATGAATATCCTCCTTAG	CCCATCCACCCCTTTCAGGA<br>AGGGGGGCGAAAGGAAAAT	CO92
		YPO1608	*ptsG*	ATGTTTAAGAACGCATTTGCAAACCTGCAAAAGGTAGGTAAATCGCTAATGTGTAGGCTGGAGCTGCTTC<br>TTAATGGTTACGAATGTATTCGTCCATATCAGTTTTCAGGTTGTCAGATTCATATGAATATCCTCCTTAG	CCAATTTATTGGGTGATGGCGG<br>GCGAGAGCACAACAGACGATGA	CO92
		YPO1851	*putA*	ACAACGTATTGACCGCACACCACACTGGCTCATTAAACAGGCTATTTTCAGTGTAGGCTGGAGCTGCTTC<br>TTGCACTTCAGTAGGCAATTGAGGCAACAGTGCTTTCTGTACGTCATTCTCATATGAATATCCTCCTTAG	AACCCTGAATTCGCCTTACCAA<br>GGGGGCTGATAAGTGAATCGGT	CO92
		YPO1941-1948	(-)	ATGCGCATTTGGCAAAAATTCTTGCTGCTCTTCTGCTGTTGGGTGTTATCGTGTAGGCTGGAGCTGCTTC<br>TTAGGGAGTTTTAGGTTTCGCGGTTTTCAGATGCTGAATGTGGTCAGCCACATATGAATATCCTCCTTAG	TCTATCTTGAAGGGCGACGGG<br>CAGAGGTGTTTTCCATCGTGA	CO92
		YPO2059-2062	*znuBC znuA* (-)	CTATCCTCGTGCTTTTTGCGACAGGCTCAGCACAAACATGGCGGCAGCACGTGTAGGCTGGAGCTGCTTC<br>CTAATCCAGTTGTAACTGCGGAATAACTTGCTTAACAATGGCCAAATACTCATATGAATATCCTCCTTAG	TAAATTTATGGGCGGGCCAG<br>GGCGGTGGGCTAATCATCAT	CO92
		YPO2201	*ompW*	ATGAAAAAAATCACTTTGGCACTGCTCGCCGTGGCCTCGTTAGTACCTACGTGTAGGCTGGAGCTGCTTC<br>TTAAAAACGATAACCTGCGCCAAACATAAATACCCATGGGGCCAAACGGCCATATGAATATCCTCCTTAG	TTCTAATCGACCGGTTGCCC<br>CGCTCGGTACCGGGTGTAAA	CO92
		YPO2976	*ybiU*	ATGGCTTCATTACACATAGACGATATCCCTGCGGCCATTAAAGCGGTCAAGTGTAGGCTGGAGCTGCTTC<br>CTAGTCTGTTATGCCCAACTGCTGTTTACCTATCTCAGTGAGGTTTTTGACATATGAATATCCTCCTTAG	TCGAGGATACGTTAGCGCCC<br>CGCGCTGCGTTTGAAGATAA	CO92
		YPO3134-3135	*ykgM rpmj2*	ATGAAGCCCAATATCCACCCTCCGTATCGGACTGTGGTATTTCACGATACGTGTAGGCTGGAGCTGCTTC<br>TCAGCGTTTTTTATGGGTTCCTCCCTGAACCGCTTTAAAGCGAGGGTTGCCATATGAATATCCTCCTTAG	TTCTTCGTTGTGGTTCGTCGC<br>TGGCGCCTAAACTCCGTCTAA	CO92
		YPO3340	(-)	ATGTCAAATAAAACAATAGCGTTCGCGTTAGTAGTGGCATCATCAGCCCCGTGTAGGCTGGAGCTGCTTC<br>CTAGAAATTAACATTCACGGATACCCAGTAATTACGGCCAGGTACAACATCATATGAATATCCTCCTTAG	GTCATCAATGGGCACCCTCC<br>CAGCCCGCCTTTGGATAAAA	CO92
		YPO3588-3589	*pyrBI*	ATGGCCAATCCGTTGTATCACAAACATATCATCTCAATTAACGATTTAAGGTGTAGGCTGGAGCTGCTTC<br>TCAATCCGCGTGTAGTACGGTCAGGTGATCGAACTCTTTCTCACAATATTCATATGAATATCCTCCTTAG	ATTCGCCACAATTTGCCGG<br>GGTTTTTGGATCGATCGGGA	CO92
		YPO3710-3716	*malM lamB malK malEFG*	TTAACCCTTGCCTTTTACACTACCAATAAAGGTTTTTCGGGCTGAAGTTGGTGTAGGCTGGAGCTGCTTC<br>TTAACCTTTCACACCACCTGCCGTTAATCCACCTACCAGCCAGCGTTGTGCATATGAATATCCTCCTTAG	TTCCAATCGAACGCAAACCG<br>GCGATGGCGGGTGATAAACA	CO92
		YPO3727	*metA*	ATGCCAATTCGGGTTCCTGATGAATTACCTGCAGTGAGTTTCTTGCGCAAGTGTAGGCTGGAGCTGCTTC<br>TCAATCAAGGGTAGGATTCATATGTCGCAAATCAAATGGCGTAATTTGGTCATATGAATATCCTCCTTAG	TTGTTGCCACACAATTGCCG<br>TGCTGCCACCTTAACGCTGA	CO92
		YPO3789-YPO3790	*metR-*	ATGATCGAACTGAAACACTTACGCACCCTGCAAGCTTTGCGTAATACTGGGTGTAGGCTGGAGCTGCTTC<br>GTGTTGCTACTGGTCATTACCACTATCTTGTGGGCTTTCTCTTTTAGTCTCATATGAATATCCTCCTTAG	CTGGCACTAAATCAACACCCGC<br>CTGCTTCCGCTACAATTGCATT	CO92
		YPO0117	*metF*	N1: CGAGCTCATTCAGCGTTTAGAAGCCGC & N2: TCCCCCGGGGCTTCTCGCTGGTTTGCGTG (upstream region)<br>N3: GCTCTAGAGATTTGTCATACCCTTGGGG & N4: TGCACTGCAGGAGCTCCATATGTCAGAAAGAGCAGC (downstream region)<br>N:5 TCCCCCGGGTACGCGCAGACCAAAACG & N6: GCTCTAGATCGCCCTTCCCAACAGTTGC (kanamycin resistance cassette)	AGAGAGTGGGTCGGTTGATC<br>TGTCGGTGATACAAGACCTG	195/P
		YPO3788	*metE*	N7: CGAGCTCGTTGCGGGTCAAAAGTGACG & N8: TCCCCCGGGAACACGCGGAAAACCCAGTG (upstream region)<br>N9: GCTCTAGAGCGAATATGGTGCTGGCAGC & N10: TGCACTGCAGGAGCTCGGGACACCAAAAAATTGGCC (downstream region)<br>N11: TCCCCCGGGTACGCGCAGACCAAAACG & N12: GCTCTAGATCGCCCTTCCCAACAGTTGC (kanamycin resistance cassette)	GATGTCAGCACCAGATCCAG<br>GATGTCAGCACCAGATCCAG	195/P
	Genes upregulated in the rat bubo and involved in the bacterial response to nitric oxide, oxidative and iron stresses [b]	YPO1949	*tehB*	ATGGAAAACACCTCTGCACAGCTAGCACCCACACTGCTATGTTACAAGAAGTGTAGGCTGGAGCTGCTTC<br>TTATGAGATTTTTTTTGCCAATAAAGTCGCAAAACGTAGTTTTATCCGGTCATATGAATATCCTCCTTAG	GAGGCAATAAGGCCAAAGCT<br>GTCACAAACAGTCACCACCA	CO92
		YPO3418-YPO3419	*aceEF*	ATGTCAGAACGTTTAAATAATGACGTGGATCCGATCGAAACCCGCGACTGGTGTAGGCTGGAGCTGCTTC<br>TTACATCACCAGACGGCGAATATCCGCCATAATGGTAGCGATATACGCGGCATATGAATATCCTCCTTAG	AACGAATCATTCGGGTTGCG<br>TTCATAACGCCCTCATCCAC	CO92
		YPO3531	*ytfE*	ATGGATTACCGCAATCAGTCTCTGGGCGCACTGGCTATCGCTATTCCTCGGTGTAGGCTGGAGCTGCTTC<br>TTATTCGCCCCTTAGCGCACGGGGAAACAGTAAATTATTTTCCAGATGAACATATGAATATCCTCCTTAG	GTAATCTCTGGCGGCAACGC<br>ATGCCAAAGGGCGACAGGGT	CO92
		YPO1854-56	(-)			CO92
		YPO1528	*fhuF*	N13: CCCCAAGCTTTCTAGACTGCCCAAATAGTTGGATCC & N14: TGCACTGCAGGCCCTATAGGGGAAGGTG (upstream region)<br>N15: TCCCCCGGGGCCATGATTGTCCGTTAGAA & N16: TAGCGAGCTCGGGCAATTAAGATGCGCAAC (downstream region)<br>N:17 TGCACTGCAGTACGCGCAGACCAAAACG & N18: TCCCCCGGGTCGCCCTTCCCAACAGTTGC (kanamycin resistance cassette)	CCCAATACAATTTCGAGCAC<br>ATGCTTTCTGCCAGAACTGC	195/P
		YPO2648-YPO2651	*nrdHIEF*	N19: TGCACTGCAGGAGCTCTGCCCTTCCGTGAGTCATTG & N20: GCTCTAGACTGGACACAGTCAGGTTTGC (upstream region)<br>N21: TCCCCCGGGGTCGCCAAACGCAGATGAAA & N22: CGGAATTCGAGCTCCGACACGTTCGTTATACGTC (downstream region)<br>N:23 GCTCTAGATACGCGCAGACCAAAACG & N24: TCCCCCGGGTCGCCCTTCCCAACAGTTGC (kanamycin resistance cassette)	TTTCGCTAAGCCCCCAATGG<br>GTAATTAGGCCGCGAGTCGG	195/P
		YPO2705	*yfiD*	N25: TAGCGAGCTCTCTTGCCCCAGAATGACGAC & N26: TCCCCCGGGACGGCTATAAATTCCAAGGG (upstream region)<br>N27: CCCCAAGCTTTCTAGACGTTGAGATTGCCAGAGGTG & N28: TGCACTGCAGAGCAACGCGATGTGATTACC (downstream region)<br>N29: TCCCCCGGGTACGCGCAGACCAAAACG & N30: TGCACTGCAGTCGCCCTTCCCAACAGTTGC (kanamycin resistance cassette)	TAATCCATGAGCCTGGTTGG<br>GCCTCTCGTCACTTAAACGG	195/P
		YPO2982	*mntH*	N31: CCGGAATTCGAGCTCTACCACAACGAGGGCTAACG & N32: GCTCTAGACGACGAGATGTATCGACAGC (upstream region)<br>N32: TGCACTGCAGGGTGGTTCTGATCGTGGTTG & N33: CCCCAAGCTTGAGCTCTTAACCCCGATGGCGAATGC (downstream region)<br>N34: GCTCTAGATACGCGCAGACCAAAACG & N35: TGCACTGCAGTCGCCCTTCCCAACAGTTGC (kanamycin resistance cassette)	CTTTACAGATTGCCAAAGCC<br>TATTGGTCACGATACTCGCC	195/P
		YPO0003	*asnA*	N36: TCCGAGCTCCTCAACCACTTCTTCTAAGC & N37: TCCCCCGGGGCTCTAATTGGCGGGAAAAG (upstream region)<br>N38: TGCACTGCAGCCCTGAAATCAGCGAGAAAG & N39: CCCCAAGCTTTCTAGATTAGCCGATAATCGCCGCTG (downstream region)	ATAAGTACCTGCTGCAATGC<br>AGCATTTTGTCTGGCCTTAC	195/P

Table S2. Primer sets used in the study

Group	ORF(s)	GENE(s)†	PRIMER SETS (5' -> 3') USED TO: GENERATE THE MUTATION[a]	PRIMER SETS (5' -> 3') USED TO: VERIFY THE MUTATION	MUTANT GENERATED IN[b]
			N40: TCCCCCGGGTACGCGCAGACCAAAACG & N41: TGCACTGCAGTCGCCCTTCCCAACAGTTGC (kanamycin resistance cassette)		
Genes not tested using pools of mutants (due to technical issues)[b]	YPO0078	*pfkA*	ATGGTTAAGAAAATTGGTGTCCTAACAAGTGGTGGTGATGCACCCGGTATGTGTAGGCTGGAGCTGCTTC<br>TTAGAACAGTTTCTTCGCTGTATCGTACCAATCTTCTTTAAATTTACTTTCATATGAATATCCTCCTTAG	CGTCATGCGCACTGGGAAT<br>CCCCATTTACGCATTGACCC	CO92
	YPO0396-YPO397	(-)	ATGTCCGACGCTAAAAAAGAGGATACTGGCGCGCTTTCGGAAGTGGGGCGGTGTAGGCTGGAGCTGCTTC<br>TTAAAGAGAAATGTGACCGACCCATTCAGTAAGCGCCATTTCGACCACGTCATATGAATATCCTCCTTAG	CCCCGAACAACTGGACGACA<br>TGGCAACCACCACCGGTATC	CO92
	YPO1091-YPO1089	(-)	ATGATTTTTTATTTTTTTCTGGCGGGCGCTTTGCAGTATAATCCTTTCGCGTGTAGGCTGGAGCTGCTTC<br>TTAGTTTCTTGCTGTATTGCTTAATGAATTAGGGCAAATATTCTCCCCATCATATGAATATCCTCCTTAG	TCAATCAATTCCCTCGCCCA<br>TCATGACGCGGGTTTGCTC	CO92
	YPO1671	(-)	TTGGGTAACATCATGAGAGATATATTAAATAAAGCATTTACTGATGATGAGTGTAGGCTGGAGCTGCTTC<br>TCACTCACTGACTTTTTGTGCATTTAGCGTTCTTAAATGTTTATAAATGGCATATGAATATCCTCCTTAG	CTCAGTCTCTAAACCAAT<br>ACTATCATAGCCAAAGTC	CO92
	YPO1790-1809	*flhBAE inv flgNMABCDEFGHIJKL*	GTGGCTGAAGATAGCGATCAGGAAAAAAGTGAAGAACCCACAGCCAGTAATGTGTAGGCTGGAGCTGCTTC<br>TTACGTATTCAACTGGAACAGAGACAAACCCTGCATATTGGTGAAGGTGGCATATGAATATCCTCCTTAG	TCCTGGGGACTCGCTTCGTT<br>TGCCACTGGCGCACCTATTT	CO92
	YPO1837	(-)	ATGAAACATAAAATCAGTTTCTGTCCCGGTGTGGGTAGCGCAGCGCATATGTGTAGGCTGGAGCTGCTTC<br>TCAGGAATATGAGGGCTTAAGGTAACGATTAACTGAGTGTATCGGTGATTCATATGAATATCCTCCTTAG	TCCGTTCCGCTGAATGTTGC<br>GGGATTGAACGGCGGAATAA	CO92
	YPO1898-YPO1897	(-)	ATGCAGAGCGTGGAGCTCAAACCAGATTTATGCAACCCGCAACTATTGGTGTGTAGGCTGGAGCTGCTTC<br>TCATTTTTTATCTTTACAATCTGCTAGCCTCAACTGCACGCTGTTACGTCCATATGAATATCCTCCTTAG	CGTGATGGGCTGGTCAATGA<br>AAGCCGTTTTCAACCGCG	CO92
	YPO2084-YPO2140	(-)	TCACTGTGATATGGCGCGGGGCATATACGGGGCATCACCCGTAAATCTTGGTGTAGGCTGGAGCTGCTTC<br>CTAGGCCTGGGTGTGCGTACAGGTATACTGGCTAGCCTGCACCTCATCAACATATGAATATCCTCCTTAG	TTGCATCAAGTTAACGCGGG<br>TGCCCTGAGTGTGCCTTGGT	CO92
	YPO2574	(-)	TTGGCTATGTCTGAAAAAAGAGTGAATCTACAAGTCATTGCATGGGCCACGTGTAGGCTGGAGCTGCTTC<br>TTAATTTGCCCATATCCCTTTAATGGGTTTTGAGAAAAAGGCAAGGACAGCATATGAATATCCTCCTTAG	TTCCCCTGATGCGAATAGGC<br>CATGCCCTGAGAGCGTTTCA	CO92
	YPO3631	(-)	ATGAAATCGTTAATACTCCTCTTAGCATTACTGATACCCGCCAGTGTAACGTGTAGGCTGGAGCTGCTTC<br>TTAGATACAAACCTGACCGTCATCATAAATAACACTGAGTGCTACCATTTCATATGAATATCCTCCTTAG	AGAAAGAGATCATGCGCGCG<br>CGTAAGCGTGCCTGTTTGGG	CO92
	YPO3645	(-)	ATGAAAAAACCATTCATGTTATCTATGTTAGTCAAACATGGGGTTGCCACGTGTAGGCTGGAGCTGCTTC<br>TCACGGAGAAGGGATATCTATGATAACGCTTGAACTGAGGACTGCTTTACCATATGAATATCCTCCTTAG	CCCATGCCTACGGACTGGTT<br>GGCCGGAGTGATGAAACCAA	CO92
	YPO3718	*pgi*	ATGAAAAATATCAATCCTAGTCAAACCGCTGCCTGGAAAGCGTTACAGCAGTGTAGGCTGGAGCTGCTTC<br>TTAACGCCAGTTCTTGAAACGGTTAATCAACGCATTGGTTGAACTGTCATCATATGAATATCCTCCTTAG	TGAACCGACGTATGGCGAGA<br>CGATCCACTGAGAACGCGAA	CO92
**Mutants tested per pool**	YPO2586-2587	(-)	GTGCGAAAAACAGAAGCTGATCGGCATACCACCGCGGTGCAGTTAGGCATGTGTAGGCTGGAGCTGCTTC<br>TTATGTAATAGGATAATTATGATTTATCCAAACATGATCGTTTTCCCAGCCATATGAATATCCTCCTTAG	CGTGAGCGCGGGCAGAAATA<br>GCAGCGACAACAGGCCAATA	CO92
	YPO2303-YPO2302	*pntAB*	ATGCGTATTGGTGTACCAAGAGAGCGGTTGGCCAATGAAGCCAGGGTTGCGTGTAGGCTGGAGCTGCTTC<br>TTAATTATTCAGTGCACGTAAAATAGCTTCTACGCTGTCTTTGGCATCACCATATGAATATCCTCCTTAG	ACATTTTTGCAACGTCGCGC<br>AACTCCCTTCCAAAGGCCGC	CO92
	YPO2288	*amn*	TTGAATAAATTACAAGATACAGGCTCTCTCGCAGTTGTTGAGGCCATTGAGTGTAGGCTGGAGCTGCTTC<br>TCAAGAATATGCGCTGTTTCCATGGATGAATGTGGAGAAAAATATAGCATCATATGAATATCCTCCTTAG	AAATCGGTTGCCCGTTCTCA<br>GGCCATCAGGATGCGGTAAA	CO92
	YPO1133	*gpmA*	ATGGCAGTAACTAAGCTAGTTTTGGTACGACACGGCGAAAGTCAATGGAAGTGTAGGCTGGAGCTGCTTC<br>TTACTTCGCTTTACCCTGGTTAGCAACCGCTGCAGCTTTAGCGGCGATTTCATATGAATATCCTCCTTAG	TGATTCGGCTGATTGAGCGC<br>TGAGTCTGGGCAGGTGCGTT	CO92
	YPO0618-YPO0617	(-)	ATGATATACGGCATCCCTGAATCTGCGATTAGTTGCTATCTCATTATTTTGTGTAGGCTGGAGCTGCTTC<br>CTAATGAAGCAGTTCCAAAGGTGGTGAGGTGTTGGTCATTTTACCCATGACATATGAATATCCTCCTTAG	GAAAAACCGCCGGAGGACCT<br>GCGTATTTGTCGGTGGCGAC	CO92
	YPO0698-YPO0700	*papCD gafB*	ATGAGAATAATTAAAAAAATTCCTATAGCAATGACTACATCATTGATCATGTGTAGGCTGGAGCTGCTTC<br>TTAAGGGTAGGCGACGGTAAAGATTGCATCCGCTTTCACATTACCGCTCTCATATGAATATCCTCCTTAG	CATCTAAAATCACAGTAACG<br>TTCAGCACGATAACGTTT	CO92
	YPO2714	*rseC*	ATGATGAAGGAATGGGCGACGGTGATATCATGGCAAAATGGTATTGCCCTGTGTAGGCTGGAGCTGCTTC<br>TTACTGAATACGCATAGCCGTTGGTGGCAAACCGATTTGCAAAATAACAGCATATGAATATCCTCCTTAG	CCGTACACTGCCGAATAT<br>GATCACAATTAACGGCA	CO92
	YPMT1,66c	(-)	ATGGACATGGCTATTGCAGGTTGCGATTTACCAACTTTTGCCACCCGGCTGTGTAGGCTGGAGCTGCTTC<br>TTATTCGTTTTCTTCAGCTTCCTTTTCATCATCACGTCTGAGGCGAGCTACATATGAATATCCTCCTTAG	TTGGCAGTTCAGACCAGCGA<br>GCCCCACAGCCTGAAGAAGA	CO92
	YPO3798-YPO3801	(-)	ATGGCCTATTATTCAAAAATTAATCATAGCTTACTTTTTACTTTAGCCTTGTGTAGGCTGGAGCTGCTTC<br>TTAACTATTAACTTCGAATACCAATGTCAGTTGCCCGTGATATTTCCCCGCATATGAATATCCTCCTTAG	ATTCAATTGCGCCTGGAGCC<br>GGCAGTTGTGGGGTGTAGGAAT	CO92
	YPO4085	*ibpA*	ATGCGTAATTCCGATCTTGCTCCATTGTATCGTTCCGCTATCGGTTTCGAGTGTAGGCTGGAGCTGCTTC<br>TTACTTAATTTCAATACGGCGTGGTTTCAAACTTTCGGGTACCAACCGCTCATATGAATATCCTCCTTAG	ATTCAGACCACGGGCCTGTC<br>ACCGCCCTGCATTGTACTGG	CO92
	YPO3826-YPO3824	*glpABC*	ATGACGAACAGTTCTCCTTATCTAGAAACGGATGTCATCATTATCGGTGGGTGTAGGCTGGAGCTGCTTC<br>TCAAGCCAATGCCCGCGCCAGTAAAGTAATCGGGTGCTCACATTTCTTACCATATGAATATCCTCCTTAG	AATCGGCGGCAATTTTGGG<br>CGGTTCACTCGCGCTCTGTT	CO92
	YPO2561-2560	(-)	TTGAACAGCGAACTACTGTGGGTGTTGACGCTACTGCTGATAGCTATTGTGTGTAGGCTGGAGCTGCTTC<br>TTAGCATTTATTTCTGGTAATATTTTCTCTCAGCACGTTTATAGGATCCACATATGAATATCCTCCTTAG	GCCACTTCTCATGGTGCGGT<br>CGCCAAAGCCCAGAGATGGA	CO92
	YPO0337	(-)	ATGCGTTATTTATTATCTCTGTCTGTTTTTCTTATCGTCTCTCTTAATCCGTGTAGGCTGGAGCTGCTTC<br>TCAATGCACGGTAAATTTATGTTTTTTTCTATCCGGGCCAAAGCACGAACCATATGAATATCCTCCTTAG	TACCTTTTGACATCCCGGCC<br>CGCGCGCAAAACTATCTGCT	CO92
	YPO2269	*bioD2*	ATGTTAACGCGTTTATTTGTGACAGGTACCGACACTGCTGTTGGTAAAACGTGTAGGCTGGAGCTGCTTC<br>TTATCCTGAAATAGCTGTAAGATCTAAATATTTTGCTAATGGTTTTTCTTCATATGAATATCCTCCTTAG	GCGCTAACGCAAGCTGTCTG<br>GGTTTGTGAAATGACCGGCG	CO92
	YPO1136-1139	*galETKM*	ATGTACGTTCTGGTAACAGGTGGTAGCGGTTACATTGGTAGCCATACCTGGTGTAGGCTGGAGCTGCTTC<br>TTATAAAGGCAACGCAATAAAGTGATAAACCGTCGTTGAGCGATAGGTCTCATATGAATATCCTCCTTAG	CTTGCAACAACGCCTCGGTC<br>GCGTAGTCGGCAGCAGTGAA	CO92
	YPO1512-YPO1514	*cdd yohJK*	ATGCGTAATATGATGTCACTTTGCTGGCAGTACCTGCGAGCTTTCACCATGTGTAGGCTGGAGCTGCTTC<br>TTAAAACGTCACGCGTTTTACCGCTGAACAGCCCAAGGCGTTCAATGTCGCATATGAATATCCTCCTTAG	TTGTATGCGCGCTCACCCA<br>CGGCTAACCCAAATCGCTGT	CO92
	YPO2905	*ail*	ATGGTTTTTATGAATAAGACATTACTGGTCTCTTCTTTAATTGCATGTTTGTGTAGGCTGGAGCTGCTTC<br>TTAGAACCGGTAACCCGCGCCAAGCATCCAAGTACCCACTTTCACATCATCATATGAATATCCTCCTTAG	TTTGTCACGGCAATGGTCGC<br>CGCGGTGAAGGACGGATTCT	CO92
	YPO1992	(-)	ATGAACAAATCAACCAAAATAGGTTCCCAAATATATACTCCATTTACCCTGTGTAGGCTGGAGCTGCTTC<br>TTACTCGCGGCGGCCTGTTGCGGTAAAAAGCACGACCATGCCTACACGGGCATATGAATATCCTCCTTAG	CTCCAGTTTGCCTGAAGGCG<br>GGTGCACGTTTCCAGCAGTG	CO92
	YPO0656	*yqiC*	ATGATTGACCCGAAAAAAATTGAGCAAATCGCCCGCCAGGTGCATGAATCGTGTAGGCTGGAGCTGCTTC<br>TTATTCCCCGGTCTTTTCGCCGTGATCCGCCGGGGCGCTGTTAAATTTTGCATATGAATATCCTCCTTAG	GCTTTTGCACCACCGACGTG<br>TACTGGCACGCCGCTGATAC	CO92
	YPO3369	(-)	ATGATCTACCCTATTTTCATCTTCAAAACCGTTGAAGGATTCGACGGTTAGTGTAGGCTGGAGCTGCTTC<br>TTAGCTGATGATTTTCTCACGGGCTAGCTCGGCCAGAAACTGGCTACGATCATATGAATATCCTCCTTAG	TTCGAGTTGCAGGAAAGCGG<br>GCGCTTCAGCGTGGCATATT	CO92
	YPO3953-YPO3955	*gntKT y3874 gntR*	CTAAGCCAACGCCCCCACGCATCGCCGGACAACCTCATCAATATCAGTATGTGTAGGCTGGAGCTGCTTC<br>ATGAAGAAAAAAAGGCCAGTGCTACAAGATGTCGCCGATATGGTGGGAGTCATATGAATATCCTCCTTAG	CGTTCATACTCTTCGGGGCG<br>AAGGGTATGCACATCGCGCT	CO92
	YPO2734-YPO2743	*ccmABCDEFGH1H2 vacJ*	ATGCTGGAAGCAAAAAATCTGACCTGTATCCGCGATGATCGCTGTTTATTGTGTAGGCTGGAGCTGCTTC<br>TCAATCGGCGGCATCTATGCTATCCAGATCGCCCTGAATAGCCTGAGCATCATATGAATATCCTCCTTAG	TCGGTTTTCCTGAACTGGCC<br>CGGCCCCATCTAACACCACA	CO92
	YPO1401-y2771	(-)	TTACTGATTCTCTTCCTCCTTATTGCCTCCTTTCTTGGTATCTTGTGGATGTGTAGGCTGGAGCTGCTTC<br>CTAATCCAACCAAGGGAGAAGCTGCCGAGCTGCATTATCAAATAAAGGGCCATATGAATATCCTCCTTAG	TGGGCACCCAGTCAGCTTGA<br>ACAGCCAACGGCAGGACAA	CO92
	YPO0841-YPO0843	*ydeMN-*	ATGGCACAGCCACATACTCAACAAGCCTATTTTCACATGATGGCCAAGCCGTGTAGGCTGGAGCTGCTTC<br>TTAAAAGAGTGACGTCATTTTTGAGCTGTAGCTGGTTACGCCTTTGCGGACATATGAATATCCTCCTTAG	TGTTCTGTTTGCCCGTTCGC<br>AGCCTCCGATCTGTGCCTGA	CO92
	YPO3008-YPO3009	(-)	ATGAAGCCGTCAAAAAGCCTGTTTCTTTTTACCTCTATCACGTTCTTAATGTGTAGGCTGGAGCTGCTTC<br>CTATTTTTCCAGGCTCTGCGCCGTGCCTTGATAACGGTAACCCGCCCCGTCATATGAATATCCTCCTTAG	AGCATGTCGTTCTCCAGCCC<br>CCGAAGTTCAGTTGCAGGCA	CO92

Table S2. Primer sets used in the study

	ORF(s)	GENE(s)[†]	PRIMER SETS (5' -> 3') USED TO GENERATE THE MUTATION[a]	PRIMER SETS (5' -> 3') USED TO VERIFY THE MUTATION	MUTANT GENERATED IN[b]
Mutants tested per pool	YPO0579-YPO0581	*uxaC uxuBA*	ATGTCGCAGTTTTTGACCGAAGACTTTTTGCTGGACACCGAGTTCGCCCGGTGTAGGCTGGAGCTGCTTC<br>TTTACAAGGTTACACCGCTCTTAAAAATGGCTAATTCACGGAAGTCATTCCATATGAATATCCTCCTTAG	ACCCGTCAAACCTAGTGCCG<br>CAACGGCTTGAGCAAACCAA	CO92
	YPO3991	*yhjJ*	ATGCAGGGCACCAAAATTCGTCTTATGGTTGGTGGGTTATTATTGGCGGCGTGTAGGCTGGAGCTGCTTC<br>TTATTGCGCGGTTGTCGGCATAACAGGTGCAGTTTCTACCGCTTCAGCAGCATATGAATATCCTCCTTAG	GTGACATCGGCAAAAGCACG<br>CCCAACAACGCTCCAGGTTC	CO92
	YPO3808-YPO3804	*livKHMGF*	ATGAAATTAACAAAAGGTAAAGTGTTGCTGGCTGGGTGTATGGCAATGGCGTGTAGGCTGGAGCTGCTTC<br>TTAGCCACCAAGATAAGCTGACCGCACCGCTTCATTCGCAAGCAAAGCTGCATATGAATATCCTCCTTAG	ATGACAGATGGGGAATTCGCG<br>AGCGAAGGAACGAACTGCAGA	CO92
	YPO1307	(-)	ATGGCGACTTCTCAGACAAAAGAACTCCCCCGCCCTCAGCTGTTTACTGTGTGTAGGCTGGAGCTGCTTC<br>TTACACAAGTGCAATATGTTGCACAAATAAGTTGATACCGGTGCCACCGACATATGAATATCCTCCTTAG	ATGCTCCCTCGCCTATCGCT<br>CGGATACGGCCCTTAAACGG	CO92
	YPO1380	*ycaD*	ATGTCCGCATATTCTCGCCCCGTGCTACTCCTGCTTTGTGGGCTTTTGCTGTGTAGGCTGGAGCTGCTTC<br>TTATACCGCAGCATAAGGCGTTTGTTGGTGATCTGGCTTTCTCAACAGCACATATGAATATCCTCCTTAG	TTGCCGCGTCTGTTATCTGC<br>TGCTGGATGAATCCGCTGGC	CO92
	YPO3342-YPO3343	*yhjA* (-)	GTGATAAGAAGAGGATTAATTGGTGGCGGTATATTGGTAATTGCAGGTTAGTGTAGGCTGGAGCTGCTTC<br>CTACTCAGAAATATTCCACTTTTGTGCTAATAAGGTTTTGGTCTTCCCGGCATATGAATATCCTCCTTAG	CCCGCGCCTTCAGTACGATT<br>ACGGCCATCGCCTCTTACTG	CO92
	YPO3719	*lysC*	ATGATTCAAGTCGCCCCCCAACAGACCCGCGATGCACCTTCTGCGACTGTGTGTAGGCTGGAGCTGCTTC<br>TTATTCAAATAAATTGTAATGTAGCGTTTGTACGACTTTGTCAGCGTCATCATATGAATATCCTCCTTAG	TCTCGCCATACGTCGGTTCA<br>CGGCCAGCAGGGAAAGTACA	CO92
	YpcD1,23	(-)	ATGAGCATTAAGGCTATATTATTATATTCATATCAGGAATATGGTCAGCAGTGTAGGCTGGAGCTGCTTC<br>TTAACGTTCACAGAAGATATCATTCTGCATAGCTCCGCCAGCATCCAGGCCATATGAATATCCTCCTTAG	AGTTAATAACCCCACCAT<br>GTCGTATCTTGTTTAATGGT	CO92
	YPO1359	*hcr*	ATGACCGATTTTATTCCAACCGATTCCCCTACCCCCTTCTGCCCTAACCGGTGTAGGCTGGAGCTGCTTC<br>TCACGCCAATTGGACATCCCCCTGAAGTTGGCAACTGCATGCCAGAACATCATATGAATATCCTCCTTAG	CCAACGGCACCAGGTTTCCT<br>GCCCCATTGATGATGCGGAT	CO92
	YPO1287-YPO1289	(-)	ATGCTGCCCACTTCACTCACCAAGGCCAAACTTGTTGGCCGAGTCTGGCGGTGTAGGCTGGAGCTGCTTC<br>TCAGATGCTGGTATATCCCGCATCGGCAGTAATGATTGAACCTGTCAGCACATATGAATATCCTCCTTAG	ACCCCTGCTGGTTCGAACGA<br>CCGTGAAGAGGCGTTGGTGT	CO92
	YPO2337	(-)	ATGTATAAAATAAAAAAATTCTCTCAAATAACGGGGATCTCTGAATTTAAGTGTAGGCTGGAGCTGCTTC<br>TTAATTTGTTGATGAAGGAGTCAATACTGTTGGTGTCTCGTGGGACCCGTCATATGAATATCCTCCTTAG	ACTACTGGCGCGGACGCTTT<br>CCGATCTGCCCGACCTGTAT	CO92
	YPO3025	(-)	ATGACTCAAGTTCAGAGTGGCATTCTGTTGGAGCACTGTCGTTTTGCCATGTGTAGGCTGGAGCTGCTTC<br>TTACAGAGATAATAAGGCCGTCAGTGAGGGTGCAAAATAATAGCTGCCGGCATATGAATATCCTCCTTAG	TGGCGTGCAAAAAGCGAA<br>TTCATCAATCCGGCCAACG	CO92
	YPO2745	(-)	ATGTCAGATGCAATTAATCGTTGTAGTGCGGAAGAAACCGCGGCTTGCTGGTGTAGGCTGGAGCTGCTTC<br>TTAACGCAGACCGAGTTGGAAAATCATTGTTTCTGCCTGGCAGGCAAAGGCATATGAATATCCTCCTTAG	TCCGACCTGTGCGAGTGTTG<br>ACAATGCGATCGCCCTGACG	CO92
	YPO0585	(-)	ATGATTCGCTTCGCTGTTATTGGCACCAATTGGATCACCGCACGCTTTGTGTGTAGGCTGGAGCTGCTTC<br>TTAACTCGCGGCGGGGTATTGAGTATCTGCCGGGAATATAACACCCGTCTCATATGAATATCCTCCTTAG	CTGCTGCGCCATCAAATACG<br>GCTATGGTAGCCCCGATCGA	CO92
	YPO2325	*dalD*	ATGAATACTGACCTGAAATCACCCCCTGCTGTGTGGCTGCATATTGGTGCGTGTAGGCTGGAGCTGCTTC<br>TTAACGGTTAGTGCTAACCCAAGCAGAGACACGATTTATGGACTGGCGTACATATGAATATCCTCCTTAG	ATGCATGGGTTATCTGCGCA<br>CACGTTGAACAATGGGCGG	CO92
	YPO0852-YPO0856	(-)	ATGTTATACCTTCACCCGAGGCTAATAATGAAAATCAATAAAATCACTGCGTGTAGGCTGGAGCTGCTTC<br>TTATTCGAGATAGCGAGTTAGCACTCGGCTTCCCCATGGGGCAAGCGTCACATATGAATATCCTCCTTAG	AATGGGCGTCAAAGCGTAGC<br>CGTCACCCGTAAACGCGTGA	CO92
	YPO2282	(-)	ATGGAGGGGCCCAGAACCTTGGGCTCCGATATTGATCCATTCCTAACGTTGTGTAGGCTGGAGCTGCTTC<br>CTATGTGAAATCGCTAGGGGTATCAATCGCCATTTCCATGCCATTAGAACCATATGAATATCCTCCTTAG	TACCCGATTGAGCGAATGGC<br>CAACGATTCCCGCCAGATTC	CO92
	YPO1862	(-)	ATGGTATTCGTCGTTCTTTGGTTCGCTATTGCAGGATTAAGTGAGTATCTGTGTAGGCTGGAGCTGCTTC<br>TCAACGTAATAGTAACAATATCAGCGCATGAACAGCCAACATCGCGACAGCATATGAATATCCTCCTTAG	AAATTGAGTCCCTGACGCGG<br>GCTTGTGGGCCATCATCCCT	CO92
	y4094	(-)	ATGAATATTGATCATGGGGCCCGCCAGCCACAATCAGTCGGTGAACGGCTGTGTAGGCTGGAGCTGCTTC<br>ATGAAAAAACGTATTCTGGCTCCTGTGGCCATCGTCTCCGTCGCACTGATCATATGAATATCCTCCTTAG	ATGGCTTCGCAGTTTACGGG<br>CGTTGTCATCTCTTCGCCGA	CO92
	YPO2958-YPO2960	*yfuABC*	ATGAAAATACCTATACCCTCTATTGGCCATGTTGCTTTACTGGCGTCTGCGTGTAGGCTGGAGCTGCTTC<br>CTACTCGGCAAAAATATGTGCCTGGCCCATGATATCCAGGCCAACAGTTACATATGAATATCCTCCTTAG	GGAAAATGCCGTTAAAGCCG<br>TCCAAGAACTGAGGCTCCGC	CO92
	YPO0049	*radC*	ATGGATGAGTGGTATGGGCAGGTGGCCCCAAGAGAGAAATTACTGAAGTAGTGTAGGCTGGAGCTGCTTC<br>CTAAAGCCATCCTCGTTCAGCAAATGAGACACATTCACCCCGGCCAACTACATATGAATATCCTCCTTAG	TCAAGCGTGAGGTCGTGGTG<br>TCGTATAGGGTATGGCCGCC	CO92
	YPO2965-YPO2969	*dmsABC* (-)	ATGAATAACAATAAAACACCGCTCACCGGCTCACTGAGTCGTCGTGAATTGTGTAGGCTGGAGCTGCTTC<br>CTATATCTGCATCTCTAATATCCTTGCTGGGCACCTGAAAACGCATTCACCATATGAATATCCTCCTTAG	AAACCTCGCCACTTTCGCAT<br>TCGCGGCGTAAGCTAACTGA	CO92
	YPO2006-YPO2008	(-)	GTGGCCATTGAGCATGATTGCTCGGTACACGGTAAAAGCGTGGCGCACCAGTGTAGGCTGGAGCTGCTTC<br>TCAGCAACCGCAGGAAATCGGTGTACCTTCTTTCCCCTCGATACCCTCAGCATATGAATATCCTCCTTAG	TGATAGGGCGGACTGACGGT<br>CGGCGTCCTCATCTGTCGTT	CO92
	YPO0345-YPO0347	*dcuA cutA-dsbD*	ATGATAGCCGTAGAATTAGTCATCGTTCTGCTGGCCATTTTTCTTGGGGCGTGTAGGCTGGAGCTGCTTC<br>TTATTTTGTAACCGCGGGGGTGTTTTGTAAATGCTGTAAGAATTGCGCAGCATATGAATATCCTCCTTAG	AAAATCTGTGCGGAAACCGG<br>GATCAAACTGCGGCAGAGCA	CO92
	YPO0319	*qor*	ATGGCAAAGCATATTCAATTTACCACCACGGGTGGACCAGATGTATTGCAGTGTAGGCTGGAGCTGCTTC<br>TTACGGGATCAGCAAGCTGGAGCCTGTCGTCTGGCGGCCCTCGAGTGTTTCATATGAATATCCTCCTTAG	TTGGCACCGAGTCACGAACC<br>AGAGGGCTCCTTAATCGGGG	CO92
	YPO1239-1250	(-)	ATGTATGAGCAAAGCCAATTTCCGTATTGGTGGGCAGGTTCTCTGGCTTTGTGTAGGCTGGAGCTGCTTC<br>TTATGCCGCTCGCACAATATAGTTAAAAGCAATATTCCGTGGTCTATTTTCATATGAATATCCTCCTTAG	AAAAAACGTTCACGCGGTCC<br>CCCGTTAGCGACCTGCCTTT	CO92
	YPO1926-YPO1928	(-)	ATGTCTAATCTTGATACTTACCAAACCCGCAGTTGGCTCTTTACGCCAGCGTGTAGGCTGGAGCTGCTTC<br>TCAGATTAGATGCATTTTTTTGGCAGCCTGCGTTAGCTCATCCCGGAAATCATATGAATATCCTCCTTAG	TCTGCGCCATAAGAAACCCG<br>CCATGCCGGAGAGCGAAAA	CO92
	YPO4093	(-)	ATGGCTATTGAACTGATCGCTATTGATATGGATGGCACGTTGCTGAATCCGTGTAGGCTGGAGCTGCTTC<br>TCAGCCAATATACTTCTCAATCGCAGTCGCCACACCATCCTCGCAGTTGGCATATGAATATCCTCCTTAG	CACTACTCTGATGGGTGAT<br>ACTTCTCAATCGCAGTCG	CO92
	YPO1150-YPO1154	*bioA bioFBCD*	TTAGCTTCCGCGATCTACCACGGCCGCAGTGAGCCGGGTTAAGGCGTGTTGTGTAGGCTGGAGCTGCTTC<br>TCACATGTTGACAGCCTCCCGTTCTGCTGCACGTAATACCGTCAAATCCACATATGAATATCCTCCTTAG	TAGGTAAGGGGCCGATGTGG<br>GCGGCGCATTCAGGTAGATC	CO92
	YPO1735	(-)	ATGCTCCGCCGTTTTTTCTCTTATTACGCCCCGTATAAAGGGCTTTTCTTGTGTAGGCTGGAGCTGCTTC<br>TCAGGCCGCACTAAACTGAGCATGGTGTAATATTGCGTAGGCACCTTGACCATATGAATATCCTCCTTAG	TTTTTTTGGGCAGACAGCGC<br>ACCACCCGGAGTCGTTAACG	CO92
	YPO3791-YPO3796	*ugpBAECQ* (-)	ATGTTTAATAATTCCATTCATAAAGTGTCGATCTGTATCGCGCTAACACTGTGTAGGCTGGAGCTGCTTC<br>TCAGTGGCGTGGTGACGGCGGCCAGTACCGTTGAATCACCAGGCTGGCGGCATATGAATATCCTCCTTAG	AGTATTAATTGTTAGCGGTC<br>GAGTGCGAATTATTCAG	CO92
	YPO0767	*gntP*	ATGGAATGGATTAATATCCTGTGGGTCATGTTGGGTATTGGCTTAATGCTGTGTAGGCTGGAGCTGCTTC<br>CTAGACAAACATGCTGATCAGCAGTACCACCGCCAAACCAACCACCGAGTCATATGAATATCCTCCTTAG	TTGGTCCGACCAATATCGCG<br>CGGAGGAAAAGGAGAGCGGA	CO92
	YPO0869-YPO0870	(-)	ATGAAAATAAACAGTAAGTTATGGATGGGCCTCCTAATGGGCATGCTAGGGTGTAGGCTGGAGCTGCTTC<br>TTATTTACCGTTAGCAGGTTCAGAGCGAAGATGATTAAGCAAAAAGACGACATATGAATATCCTCCTTAG	AAAGGCGCTTTTCGATGCTC<br>CCACATTGGTGACGACGGAA	CO92
	YPO1631	(-)	ATGGACAAATTACTCGACCGATTTTTCAACTATGTTTCTTTTGATACACAGTGTAGGCTGGAGCTGCTTC<br>TTATGATTCTTTAGCCCGCTTGGCGGTCAGCTCAGAAATTCGCATAATGACATATGAATATCCTCCTTAG	GTCAATCAACACGTCCCGGC<br>CTCAAACGCCCCTTTTCGG	CO92
	YPO2265	*tus*	ATGAACAAGTATGATTTGATAGAACGAATGAATACTCGCTTTGCTGAGTTGTGTAGGCTGGAGCTGCTTC<br>TTACGGTTCCAGTTCGACATAAAGATGCAAGCGCGGTACCAGCAAACGTACATATGAATATCCTCCTTAG	CCGGTACGTTAGGTGGCGTT<br>GCAGCAGCTATGGCATTGGG	CO92
	YPO4113-YPO4117	*pstSCAB phoU*	ATGAAACTGATGCGTACCACCGTAGCCAGCATTGTGGCAGCGACTTTATCGTGTAGGCTGGAGCTGCTTC<br>TTATTTTTCAGGCTTACCAGAGAGTAACTTCTCTAAGTCATCACCACCAACATATGAATATCCTCCTTAG	TGTTCCCCCCGAACCACTTA<br>AACCCACAGGCCCCACAAGA	CO92
	YPO0989-YPO0994	*iucABCD iutA*	ATGACTATCCCAGTAGAAACCTTAGCAACCGATGTGGCTGCTCAATGTTTGTGTAGGCTGGAGCTGCTTC<br>TCAGAACAACACTGAGTAGTTCACACCAAAAGTCCGGCCACGGCCCTTATCATATGAATATCCTCCTTAG	TAAACCGTTTTGCCCGATGC<br>GCCGCCAGAATAAACCGCTA	CO92

Table S2. Primer sets used in the study

	ORF(s)	GENE(s)[†]	PRIMER SETS (5' -> 3') USED TO: GENERATE THE MUTATION[a]	PRIMER SETS (5' -> 3') USED TO: VERIFY THE MUTATION	MUTANT GENERATED IN[b]
Mutants tested per pool	YPO1323	(-)	ATGACTACCAATTACGCTCATTATATTGATCACACTCTACTGGCGATGGAGTGTAGGCTGGAGCTGCTTC<br>CTAGTAACCTGATGCGGCGGCTTGTTGACCTGAAACAATGGCAACTCCAGCATATGAATATCCTCCTTAG	GCCCCAAAACAAGAGACTGA<br>CCCAAGGTGCGGTCAATCTT	CO92
	YPO2811-YPO2817	(-)	GTGACATTGTATGACATTAAACCTCAGTTTCAGAATCTGCTGCGACCGCTGTGTAGGCTGGAGCTGCTTC<br>TTATAGCTCCAGGATCTCTTGAATCCAATCATCCCAGCACTCGCTGCCAACATATGAATATCCTCCTTAG	GACACCGACCTGTTGACGCA<br>TGGCGCCAATCCGTATTGAT	CO92
	YPO0091	*glpF*	ATGAGCCAAACCGCTAGTTCTACCTTAAAAGGCCAATGTATTGCGGAGTTGTGTAGGCTGGAGCTGCTTC<br>TTATGCTTTACGTTCTGTGGTTATCGTTGTGGATTCTTCATCCTCAAGGGCATATGAATATCCTCCTTAG	ATGTAGCCAATCCCGCCCTG<br>GCTGCCTACATGCGACTCCA	CO92
	YPO3147	(-)	ATGACCAATACATGGGTGAAAAATGCTATCAATGAAATAGAAGCTGACTTGTGTAGGCTGGAGCTGCTTC<br>CTACAGATTATTCAATTCATTGGTAAATGGTGTTAAATCCCCAATGTTATCATATGAATATCCTCCTTAG	ATTGACCGCATCGGCCAA<br>TGCCACTGCAGCGTTATTCG	CO92
	YPO1756-1758	*manXYZ*	GTGAGTATAGCTATTATCATCGGCACACATGGGGCTGCGGCAGAACAACTGTGTAGGCTGGAGCTGCTTC<br>TTATGGTGCCAAGAAGCCAATCCAGTAGCCAAAGATACCGATAGCGAAGACATATGAATATCCTCCTTAG	CAAATCGTCAGCGACAAGCG<br>CCCCCGGTTTTGGATGAAGA	CO92
	YPO2227-YPO2228	*pyrF* (-)	ATGACGTCCGCAACTAAAACTAATAACAGTGGCTCAATATCCTCCCCAATGTGTAGGCTGGAGCTGCTTC<br>TCACCCCCCTGCCAATTTAACTTTCATCCCTTTAGCGTCTAACAGTTGTTCATATGAATATCCTCCTTAG	GGGGGACGATAAGGTGGGAT<br>CGCCCCCCCCTATTTGTACT	CO92
	YPO2539-2541	*idnOK* (-)	ATGAAGAATCTATTTTCGTTGGAAAATCGCAAAGTATTAATTACTGGCTCGTGTAGGCTGGAGCTGCTTC<br>TCATAAAAGATGATTAGCCACCTTCACCACTAGCTTACGGATGGGGGCCGCATATGAATATCCTCCTTAG	CAAGAAGCGGAGCAACTCCC<br>CGTCTGAAGGGAGCATGGCT	CO92
	YPO1551	*yeeZ*	ATGAAAAAAGTAGCCATTATTGGGCTGGGTTGGTTGGGTATGCCGTTGGCGTGTAGGCTGGAGCTGCTTC<br>TTAATTCAACGGCATCCGCGAAGGATCCGGATACAAATACTCAAACCCCACATATGAATATCCTCCTTAG	TTTGATGTGCCATTGTGCCG<br>CCGCCTTCCTGAAGCTCGAA	CO92
	YPO1753	*fcuA*	ATGAATCAGTCTCTCTCCATAAGTACGGAGCCGAAACGTTCGGCTCCTCGGTGTAGGCTGGAGCTGCTTC<br>TCAGAAGTCCATGGAGACGGATAATTTCAGCGTACGTGGATCGCCCTGATCATATGAATATCCTCCTTAG	TGTCCTCCCTGCTTATCGCC<br>CGCCGGGGGATTTCAGTAAA	CO92
	YPO3454-YPO3455	*nrdDG*	GTGAAAACAGTAGTGATTAAACGGGACGGCTGCCAGGTACCTTTTGATGAGTGTAGGCTGGAGCTGCTTC<br>TTAACGTAAACAGTGAATAACCTGATTGCTGCTGCCGCGCCAGATAAGTGCATATGAATATCCTCCTTAG	TGATGTGCTGCGGGTTGCCT<br>GGGTTTGGGCTAACGTCGTG	CO92
	YPO2434	(-)	TTGCAGCGACAACCTGTTTCATCCTCCAGAATTCTGTCGATTGGTTATGAGTGTAGGCTGGAGCTGCTTC<br>CTACCCAATTTTGCGGCACAAAAACTTGCCTTTTATCACACCATCAAAAACATATGAATATCCTCCTTAG	AACACCGTACAACCGAGGGG<br>GCTGCATTTATCGCGGCTCT	CO92
	YPO4080	*malS*	ATGAAACGTCTCACACTCCCCTTGCTACTGGTTCTTTCACCAGCAGCGATGTGTAGGCTGGAGCTGCTTC<br>TCAGTTCTTATCACCGACCCAAACGACCAGAACCTTATCGCCCTGATGTTCATATGAATATCCTCCTTAG	CCGAATCGTGATAATGGCCC<br>TTATGCTAACCGCACGCGCC	CO92
	YPO4064-YPO4065	(-)	ATGTTAGTGACACATCTTGCCGTATTCGGTGCGGGAACAGGATTGGGGATGTGTAGGCTGGAGCTGCTTC<br>ATGAAAGAACATGCAGAAATCAAACGTTTAAGCGATATGCTGGATGCGCTCATATGAATATCCTCCTTAG	CGATGCCACGGCCACACTTT<br>CGGCAAGAGCCAGTGGCTTA	CO92
	YPO4022-YPO4025	*fitABCD*	ATGCGATTACGTTTAGCTTTATTTTCGTCATTACTGGCGGCAACCTTTGCGTGTAGGCTGGAGCTGCTTC<br>TTAGCGATAATAAACCGCGATATGCTGGCCTTGTACTTTTTCTATGCTTACATATGAATATCCTCCTTAG	TAATCCTGCTCTGGCGCTGC<br>CCGAATATGGGGCTGACCAG	CO92
	YPO2803	*bglB*	ATGAATCTGAATAAAACAGGTTTTTTCACAGCGTTTACTTTCCTTTCAGCGTGTAGGCTGGAGCTGCTTC<br>TTACAATGAATTGATTTCTTGTAAGATTTCCTGCTTCAGTTTCTGCCTTTCATATGAATATCCTCCTTAG	CGTCAATGCACGCTACGGTG<br>TGTCACAGGCGTTGGCGGAT	CO92
	YPO1343-YPO1348	(-)	ATGTTATTACGTATTTTTTGGAGTTTATGCCTCTGGTGGGGGATCAGCAGGTGTAGGCTGGAGCTGCTTC<br>TCAGGCCGCCTTCTTTTTACTGCGTAACCCCAACTGAATGCACGATACCACATATGAATATCCTCCTTAG	CAAATGACGGGGAAACGTGC<br>TGCCTTCCTGCCACTCGAAT	CO92
	YPO0681	*metC*	ATGGCAGTGAATAAAGCAATCCCAGAAAAACTGGAAACAGCCTTAGTCAGGTGTAGGCTGGAGCTGCTTC<br>CTATTTTACGGCTTGAATTCGCTCAAATCCTGCGGCCAGATCGTCTTGCACATATGAATATCCTCCTTAG	ACGCTTAAGTCAGTGGCCGC<br>GCATTAACGGAGTGGTGGCC	CO92
	YPO4086	*yidQ*	ATGAGAAATATCGTTATCCCTTTTGCTACTGGCTGTTCGCTGCTGCTGACGTGTAGGCTGGAGCTGCTTC<br>CTATTGTTTTGGCGTATCACCTGTCTGATTAGTTTGCTGGTTTTGCATCTCATATGAATATCCTCCTTAG	TTCGATGAAATGGCCGCTATC<br>CGATGATAACGGTGTGGTGCA	CO92
	YPO3613-YPO3616	(-)	ATGTTTGCTCATGATAAAGCTAATAATAATAAAACCGCGGACAAGACCGGGTGTAGGCTGGAGCTGCTTC<br>TTATGGCAGTTTTCTAGGTGCTTTTTTAATTGTTGGCGGTTTCCAGGATACATATGAATATCCTCCTTAG	AAAAGGAACGTTCTCGGGGC<br>GAGATCGCGACCCAACAGGT	CO92
	YPO1254	*bglA*	ATGAGCTATCAACAGTTACCGAAAGATTTTTTATGGGGTGGCGCGGTTGCGTGTAGGCTGGAGCTGCTTC<br>TTAAAGAACTTCACCATTGCTGGCAATAACTTTTTTATACCAATCAAAACCATATGAATATCCTCCTTAG	ACCCCGCGACAGGAGATGAA<br>GGGTGAACGGCCTTCTTGAA	CO92
	YPO3986	*cdh*	GTGTCACGCTCAATAAAATGGCGTAGATACTTACTGACATTGCTGATCTTGTGTAGGCTGGAGCTGCTTC<br>TTATTGCACCGGGAGGTGAGGTAGCACCTGACAGGTGTGATCTTGCAGCTCATATGAATATCCTCCTTAG	GCAGCCTTTGGCTCGGTGAT<br>ATCAGTGCGCCCCGTTACTG	CO92
	YPO3479-YPO3480	*yhbUV*	ATGGAGCTGCTGTGTCCTGCGGGTAACTTACCTGCATTAAAGGCCGCAATGTGTAGGCTGGAGCTGCTTC<br>TTACGACACCAGTTCTAACCCGGCAACACGATGCCAGTAGCCGTTACAATCATATGAATATCCTCCTTAG	AGTTTTCCCAACACAGGCGG<br>GGGGTATCTGACGTCGCCAA	CO92
	YPMT1,03-YPMT1,42	(-)	TTATTTAATCAAATTTGCCGAATCTTCTTCCTCGTTTCCCAATATCGGCCGTGTAGGCTGGAGCTGCTTC<br>CTATTTGCCTGTTTCTTGCTTGTTTAAGCCCACTGCTTTCTGATAATAGCCATATGAATATCCTCCTTAG	ACATCCAATCTGCCGTTCGG<br>AACGAGCAGGGCCGGTTTT	CO92
	y1377	(-)	ATGACTTATTGCCAACAATGCCCCGAATCGCCAATGGCGGAGCAAGACAAGTGTAGGCTGGAGCTGCTTC<br>TCAGTGCCATCTGGAGGACGGCGGGCGACGAGTGTAGAACACGATAAATGCATATGAATATCCTCCTTAG	GTAAAGGGGCGGGAACGTCA<br>TGCAGTGCTGAGCACCCTGT	CO92
	YPO0649	*bacA*	ATGACGGATATGTATTCGCTGTTTGTGGCTTTTATTCTGGGTGTGGTCGAGTGTAGGCTGGAGCTGCTTC<br>TTACATAAAGACCCAATACACCACCGCCGCAACAATAAAGCGGTAAATGGCATATGAATATCCTCCTTAG	CTTGAAGTCGCCGCTGTGTG<br>GCTCTGGCCGAGTGGAAACA	CO92
	YPO3450-YPO3453	(-)	ATGTCTGTAGCATTAAATACCACGCCGGGAACCCCCTCCCGGCGTCATCTGTGTAGGCTGGAGCTGCTTC<br>TTACCCCATCGAATGCTCCGCCGACGCTAAATCCTCCCGTGAGTACTCCCCATATGAATATCCTCCTTAG	AAGCAAACACTGGCTGCCG<br>GGCCGCGTCATGTCACCT	CO92
	YPO2264	*fumC*	ATGGCAACTACCCGCAGTGAAAAGGATTCTATGGGGTCTATCGATGTACCGTGTAGGCTGGAGCTGCTTC<br>CTATTTCTTCATACTGCCGACCATATCTTCCGGTCGAACCCATTCATCAACATATGAATATCCTCCTTAG	TGATCTGCCGTTTGCAAAGG<br>GCGAAACCGCTACGTTTGCT	CO92
	YPO2156	(-)	ATGAACGAGAAAGTATTTACTCTCCCTGTTGTTGAACAAATTTCTCCTTAGTGTAGGCTGGAGCTGCTTC<br>TTAGGTGTTTTTACGACTACGGATGGTCATCGCCAAACGTGCTGGTGCATCATATGAATATCCTCCTTAG	GCGCCGAATAAGATTTGCCG<br>GCGCAGTGGTTTGGCTGAAT	CO92
	YPO2458	(-)	ATGAGAAAACTCCCATCACTCCGTATGTTGCGTGTGTTTGAAGAGGCCTGGTGTAGGCTGGAGCTGCTTC<br>CTAAAGCACAGCCGGTAGAGGTTTGGCTTCCCCTTTTGAGTAGCGCTTGGCATATGAATATCCTCCTTAG	ATAAAGCCGCCAGTGCGTTT<br>AACCGCAGGAGCCACATTCC	CO92
	YPO0800-YPO0801	(-)	ATGTTAAATAAAATGCTATTACAAATCACCTTCGCAAGCCTCGTGACTGCGTGTAGGCTGGAGCTGCTTC<br>CCTGTTGATCCAACCCGGTTGATTACCGAAATAATCGCCTGCAGGTACAACATATGAATATCCTCCTTAG	CGCCCATTGCTCCATCTGA<br>TTGAGCAATCTGCACACGCA	CO92
	YPO1383-YPO1384	*focA pflB*	GTGAAAGCTGACAACCCCTTCGATGCATTATTACCTTCGGCAATGGCTAAGTGTAGGCTGGAGCTGCTTC<br>TTAGATTGACTGTGTAAAGGTACGGGTAATCACATCCTGCTGCTGTTCTTCATATGAATATCCTCCTTAG	TTTCCCGACAACGTTCAGGC<br>GCACACACCAGTTCACCCGA	CO92
	YPO1331-YPO1334	*potF potGHI*	ATGTTCACCCAACGTAAAAAGTGGTTATCGGGTGTTGCTGCCGGTCTGTTGTGTAGGCTGGAGCTGCTTC<br>TCAGCTACGAGCAGCTTTACGTAATTCGCGCAATCTCTGCTTTTCCGCACCATATGAATATCCTCCTTAG	TAGCCATGCATCGGTGTTGG<br>GCGTTGCTGCTGAGGATGAA	CO92
	YPO0704-YPO0747	(-)	CTACTCCAGCACCCCAATCACATTCAAATTCATATCATCTGGGATTTCGTGTGTAGGCTGGAGCTGCTTC<br>CTATTTTTCAGCTTTCTCAGCCGCAGGTTTCACGACATTCTCGCCGTGATCATATGAATATCCTCCTTAG	AAAGGGGAGGTTTGGCAAGC<br>GGCAGCCAGCGAAAGAGAAA	CO92
	YPO0076	(-)	GTGATTGCTCAGGAGGATGCTCATGATTATCATCGCCCGATGAAAACAACGTGTAGGCTGGAGCTGCTTC<br>TCAATGGTGAATCTGAGTCAATTCTTCTTCGCTCAGACCGGTCATTTTCACATATGAATATCCTCCTTAG	GTTTCCCACTGGCATTTGCC<br>GGAACGGGAGCGGTATCAGA	CO92
	y1707	(-)	ATGACTGTATTCCCTAATCCAGTTGATGATCCCACCGGGGGTAAGCTGATGTGTAGGCTGGAGCTGCTTC<br>TTACTGGCACCATTTCTCGCCACTTTTATCTGGTGTCACCAACGCTGTTCCATATGAATATCCTCCTTAG	TACCAATGAGCGAGGGACGG<br>GCACGGCACGCTACAAATGA	CO92
	YPO3506-YPO3508	*dacB pmrAB*	ATGCATTTTTCACGAATTGTCAGTGGATTGGCCTGTTCTATCATACTCAAGTGTAGGCTGGAGCTGCTTC<br>CTATTTCTGGCTGTAAGTGGTCGCGGGTAGCCAGCATTGTGCCTTCAGACCATATGAATATCCTCCTTAG	CTTTCTCCCTCGTTGCACCG<br>GCGGAAGTTGAAGCGGAAGC	CO92
	YPO0333	*rhaR*	ATGCGGGCACCACTGCTGTTAGAAAGCCGGGATTATTTACTCTCGGAACAGTGTAGGCTGGAGCTGCTTC<br>TCACGGTTCATTTTTAGCGGGTAATACCGGGGAGCGGATAAAGCGCTGGCCATATGAATATCCTCCTTAG	TGCAAGCCCGTCATCAACTG<br>GCGGTATTGTCGGCCTGCTA	CO92

Table S2. Primer sets used in the study

	ORF(s)	GENE(s)[†]	PRIMER SETS (5' -> 3') USED TO		MUTANT GENERATED IN[b]
			GENERATE THE MUTATION[a]	VERIFY THE MUTATION	
Mutants tested per pool	YPO1858	(-)	ATGACCCAGTTATACCCACAATATAAGAAACAATTGACGACAAAAATAGTGTGTAGGCTGGAGCTGCTTC<br>TTACTCAATATGCAGTTTTCCTCCCTGCCAAGATAGCTGTGTGACCGCTGCATATGAATATCCTCCTTAG	TCGTCGCGGCTAATGACACC<br>GCCGACAACCCGCTACTGAA	CO92
	YPO0465	(-)	ATGTCTCAAGATCACAATTCATCTCAAGTCGGCTCATCCCAAATCGACTCGTGTAGGCTGGAGCTGCTTC<br>TTAATTAACTCCGTTCATCACCCTTTTAGGCATCTGAACTTTCACTTCACCATATGAATATCCTCCTTAG	TGCCATTATGTGCGTGTGGG<br>CCATCAGCACGCGTCAAACA	CO92
	YPO2847-YPO2853	*mdtABCD baeSR*	TGAAATCTCAATCCAAACGCACTTCCCGATTATTCGTATTCGTGGGGGTAGTGTAGGCTGGAGCTGCTTC<br>TTACAACAAACGGCACATCTCTGCTTCCCAGCGATACCCCATGCCATATACATATGAATATCCTCCTTAG	CGTTTGTAAAGCGGGCCAGA<br>TTCCGCTGAGGCCTTACTGG	CO92
	YPO0914	*serA*	ATGGCAAAAGTATCACTGGAGAAAGACAGAATTAAGTTTCTGTTAGTGGAGTGTAGGCTGGAGCTGCTTC<br>TTAGTACAATAAGCGAGCGCGGATCGTCCCTGGAATGGCCTTCATCGCCTCATATGAATATCCTCCTTAG	TATCACATTCCCTCATTTC<br>CCAGGCGTTTAATCATTT	CO92
	YPO3336-YPO3337	(-) *map*	ATGGGTATCGTTAAAATATCTGATTTGATGCATGAGAATCTGCGTATCGGGTGTAGGCTGGAGCTGCTTC<br>TCAGATGGCTTCGTATTCGCCAGTGCCTTCAGGCCAGGGCGTCAGCAGGTCATATGAATATCCTCCTTAG	CTTCCAGCAGATCCAAGCGG<br>CGGCTTGAATGGCATTCAGG	CO92
	YPMT1,53	(-)	ATGGTAAAATTGATATATAGAAATAAGCTGGAACCTATCAAAATGAGTGCGTGTAGGCTGGAGCTGCTTC<br>TTAACGGGGCGAGTGGAAACGTTCGGCGCCCAAGCGTTCAATCTCCACGACATATGAATATCCTCCTTAG	ACCTGCTGGAACCCAAACCC<br>ATTGTGGCCATTGGCGCT	CO92
	YPO3213	*yaiE*	ATGCTGAAATTTAATGAGTATTTTACCGGGAAAGTGAAATCTATTGGTTTGTGTAGGCTGGAGCTGCTTC<br>TTAGCTCAAATATTTGCACAAATATGAAGATGCTTCAGCCACTTGTAGGTCATATGAATATCCTCCTTAG	ATCGTTAATCAGCCGCCGTT<br>TCGGTGACTCGGGTCAGTGA	CO92
	YPO2163	(-)	ATGGATGCGTTGGAACTCTTACTTAACCGCCGCTCGGCGTCCCGTTTAACGTGTAGGCTGGAGCTGCTTC<br>TTAGAAATAGCGGACAAAAGCGCTGTGGTCTGGAGGCATGATTTTAGTTGCATATGAATATCCTCCTTAG	TCAACGCTGGCAACAAAACG<br>CGTTTCATTGCCCACCAGCA	CO92
	YPO0080-YPO0083	(-)	ATGCAAAATGCTCTTATTTGCATAATTTTTATCGAGTTACGATCATTCTTGTGTAGGCTGGAGCTGCTTC<br>TCAGATCGGCTCCATGCCATTCTTGATTTTAGCCTCTGTAGCCGCAGGCGCATATGAATATCCTCCTTAG	TTAACAGCATGCCCTTGGCG<br>CAAAGGGTTGCCTGAGGACG	CO92
	YPO0274-YPO0275	*ydeEF*	ATGAGCTGGCCCGAGTTTAAATCTCAATATCTGGTGCGCTTTTGGGCACCGTGTAGGCTGGAGCTGCTTC<br>TCAGCGCTGAATCAAATAGCGAATAGTTGGCCCATCCTGCTGAATATCCACATATGAATATCCTCCTTAG	GATGATCCACCGCGCTAGCT<br>TTTCCGCGCCAGACAAAGC	CO92
	YPO1934	(-)	ATGACACTGAAACAGCTTGAGGCCTTTTATTGGGCGGCCAGATGTTCTAAGTGTAGGCTGGAGCTGCTTC<br>TTAATCACGTTGTGGTAGTGAATGATGTAGTGGTAGTGAATGATGTAGCGCATATGAATATCCTCCTTAG	GTGCTGGTGGCGGGGTATAA<br>GCGGACGTGGATGACATTCA	CO92
	YPO1528-YPO1538	*ysuFJIHGDCBARED*	GTGTCAAATACAGCACAGTTAATTACACCTTCCCCTATAGGGCTAATTGCGTGTAGGCTGGAGCTGCTTC<br>ATGGTTTCACAACGTCAATTCAATGAAAATAATATCCAACATCTTAATGGCATATGAATATCCTCCTTAG	GCACAACAAGTCATTAGC<br>GGTAATGGCCTTTACTAT	CO92
	YPO3322-YPO3325	*dmsABCD*	ATGAAAGAGACCAACGAATCCCGGCTGTTAACGACAGGTGTCACCCGCCGGTGTAGGCTGGAGCTGCTTC<br>TTAGCGATATAACTCAACCCCAGCCGGTGTCACTTGCAATTCATGTTGCCCATATGAATATCCTCCTTAG	ACTCCCCCCTCCTCGAAAGA<br>CGCCTTGGCGTTTCTGAAGA	CO92
	YPO2492	(-)	ATGGCGTCTCATCAGATGTTTGACGTTGTGGTCAGCAAGATTGAAGTCATGTGTAGGCTGGAGCTGCTTC<br>TTATAAATCTAGCACTAACCGCGCCCCTTTTGCCCTTGAGCAACAAATCACATATGAATATCCTCCTTAG	CGAAGAACTGACGGAAGAC<br>GAAAGCCGACTCAATGAT	CO92
	YPO3327	(-)	ATGTTACAAGCTGAACGCCATAAAATTATTTGCACTCATGTACAACAACAGTGTAGGCTGGAGCTGCTTC<br>CTATTTATCGGATTCAATAATATTTAATTTAGGATTATCGCTGCGATAGTCATATGAATATCCTCCTTAG	GCGGGTAAAAAGGGGCTTAAA<br>GCAATAGGTAAGGCCGGCTTT	CO92
	y0669	(-)	ATGATTTTAAGGAAATCTGATTGGCACCCAGCCGACATCATTGCTGCACTGTGTAGGCTGGAGCTGCTTC<br>TTATGGCTGCGTTGTGGTTGGTCGTATCCTTACTTTGCGATCAAGGAGATCATATGAATATCCTCCTTAG	GACGTTGCTCCCATCCCTCA<br>TCCCGGCGCTAAGTGTCAAT	CO92
	YPO1298-YPO1300	*fruBKA*	ATGTTTCAGCTATCTACGCAAGACATTCATCTGGCTGCGCAAGCAGACAAGTGTAGGCTGGAGCTGCTTC<br>TTACGCGACCTTCGCCACCATCGCGTCAGGGCGTTTCAGCATGGCATACACATATGAATATCCTCCTTAG	CAACCGTCAGCGGGTGTGAT<br>TTCGATCTGAAACGCGCCGG	CO92
	YPO0448-YPO0450	*yfiRNB*	ATGACTACCGCTACTTGCGCAACCAACCGCGTCAGTGATGAACATCATGAGTGTAGGCTGGAGCTGCTTC<br>TTAGGACGCGGTGATAATAATGGCGACGCGGCGGTTTTCGGCTCGGCCTTCATATGAATATCCTCCTTAG	TAACGCCGATTCAACCACCA<br>TTGCCAATGGCTCTGGGTCT	CO92
	YPO2590	(-)	ATGTCAACATTAGAGAAGCTGGTTTCTGCATATTGCCATACCAGTTTGGAGTGTAGGCTGGAGCTGCTTC<br>CTACAACGGCCTGTAAATATCCCTGTAGTACGTTAATCTTTTACGAAAAACATATGAATATCCTCCTTAG	TCATACTCGCCGCTGTGGTG<br>AGTACCGTTCGTGAAGCCCG	CO92
	y2031	(-)	ATGAAAAAATTAATTTTACTTTCATCATTGTTTTTACTTTCATCTTCTGCGTGTAGGCTGGAGCTGCTTC<br>TTAGTTTCCGCAACCATCTTTCAACTCACCGTGCTCGATTAAGTCTTTAACATATGAATATCCTCCTTAG	ACGCTTGTCACCCCAGTCA<br>CCCCGGGGCAATGTATCTTA	CO92
	YPO0642-y3538	(-)	ATGTGCGGATGGGAAGCACCGAAGAAAGCCGAATTGACCGGCGCGAACGGGTGTAGGCTGGAGCTGCTTC<br>TTACGTTAATAGCGTAACTCCTTTGTTATTTCTGGGATATGCCGGGGATACATATGAATATCCTCCTTAG	TCACATTGATTGATGGGGCG<br>GCCGTTCTGAAGTGCTGCGT	CO92
	YPO1951-YPO1954	*hmsHFRS*	ATGTATAACGCATTTACAACACTACTGCGCCCGTTGCACTGGCACAGAATGTGTAGGCTGGAGCTGCTTC<br>TCATCCCTGGCGTAAATGGATCACTGGAGCAACTTCTGGCAGTTGGAGCCCATATGAATATCCTCCTTAG	ATGTCCCTTGAAATCCGT<br>GATTGTAATTGAAGGTGGTT	CO92
	YPO0955-YPO0956	(-)	ATGCTATATACCCTCGCGTTGCTGGAACCGGGCTACCCCGCCGCTCGCGTGTGTAGGCTGGAGCTGCTTC<br>TTAGAAATCGATACTGCCCTGCAACACAACCTGACGTGGTTCGCCGATAGCATATGAATATCCTCCTTAG	TTCCTGCTCTGCACTTGGGC<br>CCAAAGCCTAAAATCGCGGA	CO92
	YPO3531	*ytfE*	ATGGATTACCGCAATCAGTCTCTGGGCGCACTGGCTATCGCTATTCCTCGGTGTAGGCTGGAGCTGCTTC<br>TTATTCGCCCCTTAGCGCACGGGGAAACAGTAAATTATTTTCCAGATGAACATATGAATATCCTCCTTAG	GTAATCTCTGGCGGCAACGC<br>ATGCCAAAGGGCGACAGGGT	CO92
	YPO3070	*ygfD*	ATGAAAGATGTCACTATTTATCACAATCCACGCTGCTCCAAGAGCCGGGAGTGTAGGCTGGAGCTGCTTC<br>TTATTTCAATATCTCAAGCACTTGCTCTGGTGGGCGGCCAATACGGGCTTCATATGAATATCCTCCTTAG	TGCCCGAATTGACCAGTTGC<br>GCCCATCCATGGACTTGCCT	CO92

[†], (-) indicates that the ORFs were not named

[a], black and colored bases are those used for allelic exchange and amplification of the tag, respectively

[b], mutants were generated in the CO92 and 195/P strains using lambda Red- and pCVD442-based technologies, respectively

restriction sites are shown in red
