# Supplementary material for: New Insights into How Yersinia pestis Adapts to Its Mammalian Host during Bubonic Plague
Source: PLoS Pathog. 2014 Mar 27;10(3):e1004029. doi: 10.1371/journal.ppat.1004029 (PMC3968184; doi:10.1371/journal.ppat.1004029)
Supplement: Table S3 — Virulence of Y. pestis mutants tested individually (due to prioritization or technical issues). (PDF) [file ppat.1004029.s008.pdf]

Table S3. Virulence of *Y. pestis* mutants tested individually (due to prioritization or technical issues).

	MUTANT LACKING		FONCTION OF THE GENE PRODUCT	CATEGORY OF THE GENE	SURVIVAL (mutant vs WT)[†]		ROLE IN VIRULENCE*
	ORF(s)	GENE(s)			PERCENT	MEDIAN (in days)	
Genes upregulated in the rat bubo and mouse lungs[a]	YPO0116	*metL*	Bifunctional aspartate kinase II/homoserine dehydrogenase II	Amino acid metabolism	25 vs 0	4 vs 4	No
	YPO0117	*metF*	5,10-methylenetetrahydrofolate reductase	Energy metabolism	12.5 vs 12.5	5 vs 4	No
	YPO0158-YPO0161	*nirBDC cysG*	Nitrite reductase large subunit<br>Nitrite reductase small subunit<br>Nitrite transporter<br>Siroheme synthase<br>Nitrite reductase large subunit<br>Nitrite reductase small subunit<br>Nitrite transporter<br>Siroheme synthase	Nitrogen metabolism<br><br><br>Metabolism of cofactors and vitamins	25 vs 0	5.5 vs 5.5	No
	YPO0205- YPO0206	*bfd bfr*	Bacterioferritin-associated ferredoxin<br>Bacterioferritin	-	0 vs 12.5	4 vs 5	No
	YPO0286-YPO0279	*-hmuRSTUV*	Coproporphyrinogen III oxidase<br>Hypothetical protein<br>Hypothetical protein<br>Hemin receptor precursor<br>Hemin transport protein<br>Hemin-binding periplasmic protein<br>Hemin transport system permease<br>Hemin importer ATP-binding subunit	Metabolism of cofactors and vitamins<br>-<br>-<br><br>Transport	0 vs 0	4.5 vs 5.5	No
	YPO0342-YPO344	*-hydN fdhD*	Oxidoreductase Fe-S binding subunit<br>Electron transport protein<br>Formate dehydrogenase H	-	12.5 vs 0	5 vs 5.5	No
	YPO0426	-	Hypothetical protein	-	12.5 vs 0	5.5 vs 4	Yes
	YPO0988	-	Hypothetical protein	-	100 vs 0	Undefined vs 4	Yes
	YPO1008-YPO1012	*-ynp1 ynp2*	Integral membrane efflux protein<br>Peptidase<br>-<br>TonB-dependent outer membrane receptor pseudogene	-	12.5 vs 12.5	5 vs 4	No
	YPO1310-YPO1313	*yiuABCR*	Periplasmic substrate-binding transport protein<br>FecCD family membrane transport protein<br>Siderophore ABC transporter ATP-binding subunit	Transport	0 vs 12.5	4 vs 4	No
	YPO1386	*ansB*	L-asparaginase II	Nitrogen/Amino acid metabolism	12.5 vs 12.5	5.5 vs 4	No
	YPO1516-YPO1517	-	Hypothetical protein	-	12.5 vs 12.5	6 vs 4	No
	YPO1608	*ptsG*	Sugar ABC transporter	Transport	75 vs 12.5	Undefined vs 4	Yes
	YPO1851	*putA*	Trifunctional transcriptional regulator/proline Dehydrogenase/pyrroline-5-carboxylate dehydrogenase	Amino acid metabolism	0 vs 12.5	4 vs 4	No
	YPO1941-YPO1948	-	Hypothetical protein<br>Hypothetical protein<br>Hypothetical protein<br>Hypothetical protein<br>Hypothetical protein<br>Hypothetical protein<br>ABC transporter ATP-binding protein<br>Thioredoxin<br>Cytochrome	-	0 vs 0	4 vs 4	No
	YPO2059-YPO2062[c]	*znuBC znuAyebA*	High-affinity zinc transporter membrane component<br>High-affinity zinc transporter ATPase<br>High-affinity zinc transporter periplasmic component<br>Hypothetical murein DD-endopeptidase	Transport<br><br><br>Peptidoglycan	62.5 vs 0	Undefined vs 4	Yes
	YPO2201	*ompW*	Outer membrane protein	-	12.5 vs 0	4 vs 4	No
	YPO2976	*ybiU*	Hypothetical protein	-	12.5 vs 0	4 .5 vs 4	No
	YPO3134-YPO3135	*rpmE2*	50S ribosomal protein L31<br>50S ribosomal protein L36	Translation	12.5 vs 0	4 vs 4	No

	MUTANT LACKING		FONCTION OF THE GENE PRODUCT	CATEGORY OF THE GENE	SURVIVAL (mutant vs WT)[†]		ROLE IN VIRULENCE*
	ORF(s)	GENE(s)			PERCENT	MEDIAN (in days)	
	YPO3340	-	Siderophore receptor	-	0 vs 0	4 vs 4	No
	YPO3588	*pyrBI*	Aspartate carbamoyltransferase catalytic subunit<br>Aspartate carbamoyltransferase regulatory subunit	Nucleotide/Amino acid metabolism	0 vs 12.5	4 vs 4	No
	YPO3710-YPO3716	*malM lamB malK-malEFG*	Maltose regulon periplasmic protein<br>Maltoporin<br>Maltose ABC transporter ATP-binding protein<br>Hypothetical protein<br>Maltose ABC transporter substrate-binding protein<br>Maltose transporter membrane protein<br>Maltose ABC transporter permease	Membrane transport	0 vs 12.5	4 vs 4	No
	YPO3727	*metA*	Homoserine O-succinyltransferase	Amino acid metabolism	12.5 vs 0	7 vs 4	Yes
	YPO3788	*metE*	5-methyltetrahydropteroyltriglutamate--homocysteine S-methyltransferase	Amino acid metabolism	0 vs 12.5	5 vs 5	No
	YPO3789-YPO3790	*metR-*	LysR family transcriptional regulator<br>Hypothetical protein	-	12,5 vs 0	4 vs 4	No
Genes upregulated in the rat bubo and involved in the bacterial response to nitric oxide, oxidative and iron stresses[b]	YPO1528	*fhuF*	Ferric iron reductase involved in ferric hydroxamate transport	-	0 vs 0	4 vs 4	No
	YPO1854-YPO1856	*efeUOB*	Ferrous iron transport	-	12.5 vs 12.5	4 vs 4.5	No
	YPO1949	*tehB*	Tellurite resistance protein	-	0 vs 12.5	3 vs 4	No
	YPO2652-YPO2648	*nrdHIEF*	Ribonucleotide-diphosphate reductase subunit beta<br>Ribonucleotide-diphosphate reductase subunit alpha<br>Ribonucleotide reductase stimulatory protein<br>Glutaredoxin	Nucleotide metabolism	12.5 vs 0	5 vs 4	Yes
	YPO2705	*yfiD*	Autonomous glycyl radical cofactor	-	0 vs 0	4 vs 4	No
	YPO2982	*mntH*	Manganese transport protein	-	0 vs 0	4 vs 4	No
	YPO3418-YPO3419	*aceEF*	Pyruvate dehydrogenase subunit E1<br>Dihydrolipoamide acetyltransferase	Carbohydrate metabolism	100 vs 12.5	Undefined vs 4	Yes
	YPO3531	*ytfE*	Iron-sulfur cluster repair di-iron protein	-	0 vs 0	4 vs 4	No
Genes that were not tested using pools of mutants, due to technical issues[b]	YPO0003	*asnA*	Asparagine synthetase	Amino acid metabolism	0 vs 0	4 vs 4	No
	YPO0078	*pfkA*	6-phosphofructokinase	Glycolyse	0 vs 0	4.5 vs 4	No
	YPO0396-YPO0397	-	Hypothetical proteins	-	0 vs 0	3.5 vs 4	No
	YPO1091-YPO1089	-	Prophage protein<br>Prophage DNA primase<br>Regulatory prophage protein	-	12.5 vs 0	5.5 vs 4	Yes
	YPO1671	-	DNA-binding protein	-	0 vs 0	4 vs 4	No
	YPO1790-1809	*flhBAE inv flgNMABCDEFGHIJKL*	Flagellar biosynthesis protein	-	0 vs 0	4 vs 4	No
	YPO1837	-	AraC-family transcriptional regulator	-	0 vs 0	3 vs 4	No
	YPO1898-YPO1897	-	Hypothetical proteins	-	0 vs 0	4 vs 4	No
	YPO2084-YPO2140	-	Phage proteins	-	0 vs 0	5 vs 4	No
	YPO2574	-	Hypothetical protein	-	0 vs 25	4 vs 4.5	No
	YPO3631	-	Hypothetical protein	-	0 vs 0	4 vs 4	No
	YPO3645	-	Hypothetical protein	-	0 vs 0	3 vs 4	No
	YPO3718	*pgi*	Glucose-6-phosphate isomerase	Carbohydrate metabolism	25 vs 0	5 vs 4	No

[†], virulence was determined after intradermal inoculation of 10 CFU. Groups of 8 animals per group were used; *, a gene was considered to be necessary ("Yes") or not necessary ("No") for virulence if the survival curve for animals infected with the mutant and the wild-type strain were significantly different ($p<0.05$) or not significantly different ($p>0.05$) in a Gehan-Breslow-Wilcoxon test;[a], virulence was evaluated in mice; [b], virulence was evaluated in rats;[c] *ypo2062* is the gene required for virulence (see Figure 1)

Deletion of the genes highlighted in grey significantly decreased the virulence ($p<0.05$ in a Gehan-Breslow-Wilcoxon test)
