# Supplementary material for: New Insights into How Yersinia pestis Adapts to Its Mammalian Host during Bubonic Plague
Source: PLoS Pathog. 2014 Mar 27;10(3):e1004029. doi: 10.1371/journal.ppat.1004029 (PMC3968184; doi:10.1371/journal.ppat.1004029)
Supplement: Table S4 — The role of Y. pestis genes in plague, on the basis of per-pool screening. (PDF) [file ppat.1004029.s009.pdf]

Table S4. The role of *Y. pestis* genes in plague, on the basis of per-pool screening.

MUTANT LACKING		FUNCTION OF THE GENE PRODUCT	CATEGORY OF THE GENE	VIRULENCE DATA OBTAINED USING				ROLE IN VIRULENCE*
				PER POOL MUTANTS[†]		INDIVIDUAL MUTANT[‡]		
				ARCI ± SE IN		SURVIVAL (mutant vs WT)		
ORF(s)	GENE(s)			LYMPH NODE	SPLEEN	PERCENT	MEDIAN (in days)	
YPO2586-YPO2587	-	Hypothetical protein	Inositol phosphate metabolism	0.0 ± 0	0.0 ± 0			
YPO2303-YPO2302	*pntAB*	NAD(P) transhydrogenase	Nicotinate and nicotinamide metabolism	0.0 ± 0	0.0 ± 0			
YPO2288	*amn*	AMP nucleosidase	Purine metabolism	0.0 ± 0	0.0 ± 0			
YPO1133	*gpmA*	Phosphoglyceromutase	Glycolysis / Gluconeogenesis	0.0 ± 0	0.0 ± 0			
YPO0618-YPO0617	-	Hypothetical protein	-	0.1 ± 0	0.1 ± 0			
**YPO0698-YPO0700**	***papCD gafB***	**Fimbriae**	**Attachment/colonization**	**0.3 ± 0**	**0.0 ± 0**	100 vs 25%	undefined vs 4.5	YES
YPO2714	*rseC*	SoxR reducing system protein	Regulation	0.5 ± 0	0.9 ± 1			
**YPMT1.66c**	**-**	**Putative DNA-binding protein**	**-**	**0.5 ± 0**	**1.2 ± 1**	50 vs 0%	11 vs 4	YES
**YPO3798-YPO3801**	**-**	**Putative frimbria**	**Attachment/colonization**	1.1 ± 1	1.1 ± 1	0 vs 0%	4 vs 4	NO
YPO4085	*ibpA*	Heat shock protein	Molecular chaperone	0.9 ± 0	1.6 ± 1			
YPO3826-YPO3824	*glpABC*	Glycerol-3-phosphate dehydrogenase	Glycerophospholipid metabolism	5.4 ± 5	0.8 ± 0			
**YPO2561-YPO2560**	**-**	**Putative ion transport protein**	**-**	**4.6 ± 5**	**1.4 ± 1**	100 vs 25%	undefined vs 4.5	YES
**YPO0337**	**-**	**Hypothetical protein**	**-**	**9.5 ± 9**	**5.4 ± 5**	12.5 vs 25%	6.5 vs 4.5	YES
**YPO2269**	***bioD2***	**Putative dithiobiotin synthetase**	**Biotin metabolism**	**2.6 ± 2**	**12.6 ± 10**	0 vs 0%	4 vs 4	NO
YPO1139-YPO1136	*galETKM*	UDP-galactose-4-epimerase<br>Galactose-1-phosphate<br>Uridylyltransferase<br>Galactokinase<br>Pseudo-protein	Galactose metabolism	3.8 ± 2	10.2 ± 10			
YPO1514-YPO1512	*yohJK cdd*	Hypothetical proteins<br>Cytidine deaminase	Pyrimidine metabolism	7.1 ± 5	31.9 ± 14			
**YPO2905**	***ail***	**Attachment invasion locus protein**	**Attachment/colonization**	**8.3 ± 5**	**14.2 ± 8**	100 vs 0%	undefined vs 4.5	YES
**YPO1992**	**-**	**Hypothetical protein**	**-**	**8.6 ± 6**	**15.0 ± 10**	0 vs 0%	3.5 vs 4	NO
**YPO0656**	***yqiC***	**Hypothetical protein**	**-**	**12.5 ± 13**	**0.0 ± 0**	50 vs 25%	11 vs 4.5	YES
**YPO3369**	**-**	**Hypothetical protein**	**-**	**22.0 ± 13**	**4.4 ± 2**	50 vs 0%	11 vs 4	YES
YPO3953-YPO3955	*gntVM y3874 gntR*	Gluconokinase<br>Gluconate permease<br>Hypothetical protein<br>Gnt-I transcriptional repressor	Pentose phosphate pathway<br>Transport<br>-<br>Regulation	11.7 ± 9	7.9 ± 5			
YPO2734-YPO2743	*ccmABCDEFGH1H2 vacJ*	Heme exporter proteins<br>Cytochrome c biogenesis proteins<br>Thiol:disulfide interchange protein<br>Hypothetical protein	Transport<br>Cytochrome c-type biogenesis<br>-<br>-	12.0 ± 10	7.7 ± 3			
**YPO0989-YPO0994**	***iucABCD iutA***	**Putative ferric siderophore synthetase**<br>**Putative siderophore transporter**	**Iron transport**	**20.2 ± 13**	**10.1 ± 10**	0 vs 0%	4 vs 4	NO
YPO0841-YPO0843	*ydeMN-*	Putative regulatory protein<br>Putative sulfatase<br>Hypothetical protein	-	11.5 ± 9	12.5 ± 10			
**YPO3008-YPO3009**	**-**	**Two compoent system**	**Regulation**	**13.6 ± 10**	**12.6 ± 10**	0 vs 25%	3 vs 4.5	NO
YPO0579-YPO0581	*uxaC uxuBA*	Glucuronate isomerase<br>Altronate oxidoreductase<br>Altronate hydrolase	Pentose and glucuronate interconversions	14.4 ± 10	17.4 ± 10			
**YPO3991**	***yhjJ***	**Putative insulinase family protease**	**-**	**14.5 ± 10**	**18.8 ± 11**	62.5 vs 0%	undefined vs 4	YES
YPO3808-YPO3804	*livKHMGF*	Leucine/isoleucine/valine transporter	Transport	14.7 ± 10	16.2 ± 10			
YPO1307	-	Hypothetical protein	-	15.2 ± 9	14.0 ± 7			
YPO1380	*ycaD*	Putative MFS transporter	Transport	15.4 ± 12	16.0 ± 12			
YPO3342-YPO3343	*yhjA -*	Putative cytochrome c peroxidase<br>Probable extracellular solute-binding protein	-	15.5 ± 10	15.9 ± 10			
YPO3719	*lysC*	Aspartate kinase	Amino-acids metabolism	18.2 ± 11	15.1 ± 10			
YPO2325	*dalD*	Putative mannitol dehydrogenase	-	19.1 ± 11	13.1 ± 10			
**YPO1359**	***hcr***	**NADH oxidoreductase**	**-**	**14.9 ± 11**	**21.2 ± 11**	12.5 vs 0%	4 vs 4	NO

MUTANT LACKING		FUNCTION OF THE GENE PRODUCT	CATEGORY OF THE GENE	VIRULENCE DATA OBTAINED USING				ROLE IN VIRULENCE*
				PER POOL MUTANTS[†]		INDIVIDUAL MUTANT[‡]		
				ARCI ± SE IN		SURVIVAL (mutant vs WT)		
ORF(s)	GENE(s)			LYMPH NODE	SPLEEN	PERCENT	MEDIAN (in days)	
YPO1289-YPO1287	-	Hypothetical protein<br>D-3-phosphoglycerate dehydrogenase<br>Putative short chain oxidoreductase	Amino-acids metabolism	15.6 ± 9	32.7 ± 15			
**YPO2337**	**-**	**Putative regulatory protein**	**Regulation**	**16.7 ± 10**	**25.4 ± 11**	12.5 vs 0%	4 vs 4	NO
YPO3025	-	Hypothetical protein	-	17.8 ± 10	39.1 ± 14			
YPO2745	-	Hypothetical protein	-	18.9 ± 11	37.1 ± 15			
YPO0585	-	Putative MocA-family oxidoreductase	-	19.3 ± 11	27.6 ± 14			
YpcD1.23	-	Hypothetical protein	-	26.2 ± 12	10.9 ± 5			
YPO0856-YPO0852	*malE2FG-bgaB*	Putative maltose/maltodextrin transport<br>Putative galactosidase	Transport<br>Galactose metabolism	32.1 ± 15	14.3 ± 10			
YPO2282	-	Hypothetical protein	-	31.4 ± 13	15.4 ± 11			
YPO1862	-	Hypothetical protein	-	25.4 ± 13	16.7 ± 12			
y4094	-	Hypothetical protein	-	20.5 ± 11	17.3 ± 10			
YPO2958-YPO2960	*yfuABC*	Iron transporter	Stress response	23.0 ± 13	17.9 ± 10			
YPO0049	*radC*	DNA repair protein	Stress response	29.2 ± 14	18.1 ± 11			
YPO2965-YPO2969	*dmsABC*	Anaerobic dimethyl sulfoxide reductase	Respiration	30.5 ± 14	18.3 ± 11			
**YPO2006-YPO2008**	**-**	**Putative esterase**<br>**Putative rhodanese-like protein**	-	**20.7 ± 13**	**18.5 ± 12**	0 vs 0%	3.5 vs 4	NO
YPO0347-YPO0345	*dcuA cutA-dsbD*	Anaerobic C4-dicarboxylate transporter<br>Divalent-cation tolerance protein<br>Thiol:disulfide interchange protein	Transport	20.1 ± 13	20.1 ± 13			
YPO0319	*qor*	NADPH2:quinone reductase	-	20.8 ± 11	19.6 ± 11			
**YPO1239-YPO1250**	**-**	**Putative bacteriophage proteins**	**Phage**	**23.5 ± 13**	**20.0 ± 13**	0 vs 0%	4 vs 4	NO
YPO1928-YPO1926	-	Putative citrate lyase beta chain<br>Putative transcription regulatory protein<br>Putative 4-hydroxybutyrate co-A transferase	Citrate cycle (TCA cycle); regulation	31.1 ± 15	20.2 ± 13			
YPO4093	-	Hypothetical protein	-	20.8 ± 11	32.7 ± 14			
**YPO1150-YPO1154**	***bioA bioFBCD***	**Biotin synthase enzymes**	**Biotin metabolism**	**21.1 ± 12**	**22.3 ± 12**	0 vs 0%	4.5 vs 4	NO
YPO1735	-	Putative ATP-binding protein	Transport	21.6 ± 12	34.8 ± 15			
YPO3796-YPO3791	*ugpBAECQ*	Glycerol-3-phosphate transporter<br>Glycerophosphodiester phosphodiesterase<br>Hypothetical protein	Transport<br>Glycerophospholipid metabolism<br>-	21.8 ± 11	21.4 ± 12			
YPO0767	*gntP*	Fructuronate transporter	Transport	22.6 ± 11	33.6 ± 13			
YPO0870-YPO0869	-	Hypothetical proteins	-	22.9 ± 13	26.7 ± 14			
YPO1631	-	Peptidase T	-	24.7 ± 13	28.8 ± 13			
YPO2265	*tus*	DNA replication terminus site-binding protein	DNA replication	24.7 ± 12	29.6 ± 14			
YPO4117-YPO4113	*pstSCAB phoU*	Phosphate ABC transporter<br>Transcriptional regulator	Transport<br>Regulation	26.3 ± 13	35.0 ± 13			
YPO1401-y2771	-	Hypothetical proteins	-	26.7 ± 14	26.5 ± 14			
YPO1323	*deoC*	deoxyribose-phosphate aldolase	Pentose phosphate pathway	26.7 ± 11	21.6 ± 11			
YPO2817-YPO2811	-	CDP-alcohol phosphatidyltransferase<br>Phosphatidate cytidylyltransferase<br>Putative acyltransferase<br>Hypothetical protein<br>Putative dual specificity phosphatase<br>Hypothetical proteins	Glycerophospholipid metabolism<br>-<br>-	26.7 ± 15	22.0 ± 11			
YPO0091	*glpF*	Glycerol uptake facilitator protein	Transport	28.1 ± 12	33.2 ± 14			
YPO3147	-	Putative cysteine synthase	Amino-acids metabolism	28.7 ± 13	34.7 ± 13			
YPO1758-YPO1756	*manXYZ*	Mannose PTS system	Fructose and mannose metabolism	28.7 ± 12	36.1 ± 12			
YPO2227-YPO2228	*pyrF* -	Orotidine-5'-phosphate decarboxylase<br>Translation initiation factor SUI1	Pyrimidine metabolism<br>Translation	30.0 ± 15	28.2 ± 14			
YPO2539-2541	*idnOK* -	Gluconate 5-dehydrogenase<br>Putative thermosensitive gluconokinase	Pentose phosphate pathway	30.0 ± 13	22.6 ± 12			

MUTANT LACKING		FUNCTION OF THE GENE PRODUCT	CATEGORY OF THE GENE	VIRULENCE DATA OBTAINED USING				ROLE IN VIRULENCE*
				PER POOL MUTANTS†		INDIVIDUAL MUTANT‡		
				ARCI ± SE IN		SURVIVAL (mutant vs WT)		
ORF(s)	GENE(s)			LYMPH NODE	SPLEEN	PERCENT	MEDIAN (in days)	
YPO1551	*yeeZ*	Hypothetical protein	-	30.2 ± 12	34.0 ± 14			
YPO1753	*fcuA*	Ferrichrome receptor protein	Stress response	31.2 ± 13	26.9 ± 13			
YPO3454-YPO3455	*nrdDG*	Ribonucleoside triphosphate reductase	NTP synthesis	31.6 ± 15	24.9 ± 12			
YPO2434	-	Hypothetical protein	-	31.8 ± 14	40.0 ± 15			
YPO4080	*malS*	periplasmic alpha-amylase precursor	Starch and sucrose metabolism	32.3 ± 14	26.7 ± 13			
YPO4064-YPO4065	-	Hypothetical proteins	-	33.3 ± 13	31.8 ± 12			
YPO4022-YPO4025	*fitABCD*	Iron transporter	Stress response	34.4 ± 14	39.2 ± 14			
YPO2803	*bglB*	Putative beta-glucosidase	Starch and sucrose metabolism	35.1 ± 15	31.5 ± 15			
YPO1343-YPO1348	*fiuABC*	Fiu iron transporter<br>Hypothetical proteins	Stress response	35.6 ± 15	27.3 ± 14			
YPO0681	*metC*	Cystathionine beta-lyase	Amino-acids metabolism	36.5 ± 15	43.7 ± 14			
YPO4086	*yidQ*	Hypothetical protein	-	36.8 ± 14	29.7 ± 13			
YPO3613-YPO3616	-	Hypothetical proteins	-	37.0 ± 11	34.1 ± 12			
YPO1254	*bglA*	6-phospho-beta-glucosidase	Glycolysis / Gluconeogenesis	38.0 ± 15	37.8 ± 15			
YPO3986	*cdh*	CDP-diacylglycerol pyrophosphatase	Glycerophospholipid metabolism	38.4 ± 13	36.3 ± 15			
YPO3479-YPO3480	*yhbUV*	Putative protease<br>Hypothetical protein	-	38.7 ± 13	42.4 ± 15			
YPMT1.03-YPMT1.42	-	Putative phage proteins<br>Hypothetical proteins<br>Putative regulator<br>Putative exonuclease<br>Putative periplasmic protein	Phage<br>-<br>Regulation<br>-<br>-	39.4 ± 15	34.1 ± 13			
y1377	-	Hypothetical protein	-	39.4 ± 12	44.6 ± 14			
YPO0649	*bacA*	Undecaprenyl pyrophosphate phosphatase	Peptidoglycan biosynthesis	39.6 ± 15	38.7 ± 14			
YPO3450-YPO3453	-	Putative transporter	Transport	39.6 ± 14	38.6 ± 15			
YPO2264	*fumC*	Fumarate hydratase	Citrate cycle (TCA cycle)	39.9 ± 16	30.8 ± 15			
YPO2156	-	Glucose-6-phosphate 1-epimerase	Glycolysis / Gluconeogenesis	40.1 ± 16	51.9 ± 17			
**YPO2458**	**-**	**Putative transcriptional regulator**	**Regulation**	**40.6 ± 15**	**38.0 ± 15**	0 vs 0%	3.5 vs 4	NO
YPO0800-YPO0801	-	Hypothetical proteins	-	41.1 ± 15	28.1 ± 12			
YPO1384-YPO1383	*focA pflB*	Formate transporter<br>Formate acetyltransferase	Pyruvate metabolism	41.1 ± 15	40.1 ± 16			
YPO1331-YPO1334	*potF potGHI*	Putrescine transporter	Transport	41.3 ± 16	34.6 ± 15			
YPO0704-YPO0747	-	Flagella synthetis and assembling system	-	42.9 ± 14	39.5 ± 14			
YPO0076	-	Hypothetical protein	-	43.1 ± 15	46.6 ± 15			
y1707	-	Hypothetical protein	-	43.3 ± 16	43.4 ± 14			
YPO3506-YPO3508	*dacB pmrAB*	D-alanyl-D-alanine<br>Carboxypeptidase/endopeptidase<br>Two-component system	Peptidoglycan<br>Regulation	43.5 ± 14	38.1 ± 14			
YPO0333	*rhaR*	Transcriptional activator	Regulation	43.5 ± 15	41.1 ± 16			
YPO1858	-	N-acetylneuraminic acid mutarotase	-	45.5 ± 16	51.0 ± 16			
YPO0465	-	Putative transport protein	Transport	45.8 ± 14	32.4 ± 14			
YPO2847-YPO2853	*mdtABCD baeSR*	Putative multidrug efflux transporter<br>Two-component system	Transport<br>Regulation	46.1 ± 14	58.9 ± 15			
YPO0914	*serA*	D-3-phosphoglycerate dehydrogenase	Amino-acids metabolism	47.0 ± 13	49.2 ± 15			
YPO3336-YPO3337	*- map*	Hypothetical protein<br>Methionine aminopeptidase	-<br>-	47.1 ± 16	43.7 ± 15			
YPMT1,53	-	Hypothetical protein	-	47.8 ± 13	46.1 ± 13			
YPO3213	*yaiE*	Hypothetical protein	-	48.2 ± 14	34.3 ± 13			
YPO2163	-	Hypothetical protein	-	48.5 ± 16	41.3 ± 15			
YPO0083-YPO0080	-	Hypothetical proteins<br>Putative Transporter<br>Putative Transporter	-<br>Transport<br>Transport	49.7 ± 15	45.6 ± 15			

MUTANT LACKING		FUNCTION OF THE GENE PRODUCT	CATEGORY OF THE GENE	VIRULENCE DATA OBTAINED USING				ROLE IN VIRULENCE*
				PER POOL MUTANTS[†]		INDIVIDUAL MUTANT[‡]		
				ARCI ± SE IN		SURVIVAL (mutant vs WT)		
ORF(s)	GENE(s)			LYMPH NODE	SPLEEN	PERCENT	MEDIAN (in days)	
YPO0274-YPO0275	*yedEF*	Putative inner membrane protein<br>Hypothetical protein	-	50.0 ± 17	40.0 ± 16			
YPO1934	-	Putative transcriptional regulator	Regulation	50.2 ± 17	40.6 ± 16			
YPO1528-YPO1538	*ysuFJIHGDCBARED*	Iron transporter	Stress response	50.4 ± 15	47.4 ± 14			
YPO3325-YPO3322	*dmsABCD*	Anaerobic dimethyl sulfoxide reductase	Respiration	51.4 ± 12	42.0 ± 10			
YPO2492	-	Putative dioxygenase beta subunit	-	51.5 ± 16	61.2 ± 16			
YPO3327	-	Putative regulator	Regulation	54.5 ± 17	61.4 ± 15			
y0669	-	Putative regulator for maltose metabolism	Regulation	54.5 ± 15	60.8 ± 16			
YPO1298-YPO1300	*fruBKA*	PTS system fructose-specific transporter	Transport	54.9 ± 14	53.7 ± 16			
YPO0450-YPO0448	*yfiRNB*	Hypothetical proteins	-	55.8 ± 17	48.7 ± 15			
YPO2590	-	Hypothetical protein	-	56.3 ± 14	52.3 ± 14			
y2031	-	Hypothetical protein	-	57.1 ± 15	63.4 ± 14			
YPO0642-y3538	-	Hypothetical proteins	-	57.2 ± 15	65.2 ± 14			
YPO1951-YPO1954	*hmsHFRS*	Hemin storage system	Biofilm	57.4 ± 15	37.8 ± 14			
YPO0955-YPO0956	-	Putative iron transporter	Stress response	63.2 ± 14	53.7 ± 16			
**YPO3531**	***ytfE***	**Iron-sulfur cluster repair di-iron protein**	**Regulator of cell morphogenesis and NO signaling**	**70.3 ± 14**	**71.3 ± 14**	0 vs 0%	4 vs 4	NO
YPO3070	*ygfD*	Putative arsenate reductase	-	76.1 ± 12	80.9 ± 11			

[†], data were obtained from groups of 10 rats inoculated intradermally with a pool of 5 mutants (with 20 CFU of each mutant)

[‡], data were obtained from groups of 8 rats inoculated intradermally with 10 CFU

*, a gene was considered to be necessary ("Yes") or not necessary ("No") for virulence if the survival curve for animals infected with the mutant and the wild-type strain were significantly different ($p<0.05$) or not significantly different ($p>0.05$) in a Gehan-Breslow-Wilcoxon test

Mutants highlighted in light grey (ARCI≤10) has a high to moderate likelihood of being virulence-attenuated

Mutants highlighted in dark grey (10<ARCI<20) has a moderate to low likelihood of being virulence-attenuated

Mutants which are not highlighted (ARCI≥20) has no likelihood of being virulence-attenuated
