# Supplementary material for: New Insights into How Yersinia pestis Adapts to Its Mammalian Host during Bubonic Plague
Source: PLoS Pathog. 2014 Mar 27;10(3):e1004029. doi: 10.1371/journal.ppat.1004029 (PMC3968184; doi:10.1371/journal.ppat.1004029)
Supplement: Table S5 — Role of metal acquisition systems in plague. (PDF) [file ppat.1004029.s010.pdf]

Table S5. Role of metal acquisition systems in plague

METAL ACQUISITION SYSTEMS		MUTANT LACKING: ORF(s)	MUTANT LACKING: GENE(s)	DIFFERENTIAL EXPRESSION IN VIVO [ə]	VIRULENCE DATA OBTAINED USING: PER POOL MUTANTS[†] ARCI ± SE IN LYMPH NODE	PER POOL MUTANTS[†] ARCI ± SE IN SPLEEN	INDIVIDUAL MUTANT[‡] SURVIVAL (mutant vs WT) PERCENT	INDIVIDUAL MUTANT[‡] SURVIVAL (mutant vs WT) MEDIAN (in days)	ROLE IN VIRULENCE*
Iron	Siderophores	YPO0989-YPO0994[ϕ]	*iucABCD iutA*	+	20.2 ± 13	10.1 ± 10	0 vs 0	4 vs 4	No
		YPO1753	*fcuA*	+	31.2 ± 13	26.9 ± 13	Not determined		No
		YPO1528-YPO1538	*ysuFJIHGDCBARED*	+	50.4 ± 15	47.4 ± 14	Not determined		No
		YPO0955-YPO0956	-	+	63.2 ± 14	53.7 ± 16	Not determined		No
		YPO1008-YPO1012[a]	*-ynp1 ynp2*	+	Not determined		12.5 vs 12.5	5 vs 4	No
		YPO3340[a]	-	+	Not determined		0 vs 0	4 vs 4	No
		YPO1906-YPO1916	*fyuA ybtETU irp1 irp2 ybtAPQXS*	+	Not determined		Previously studied [10]		Yes
		YPO0770-YPO0777	*ynp3 to ynp11*	↔	Not determined		Not determined		To be tested
	ABC transporters	YPO2958-YPO2960	*yfuABC*	+	23.0 ± 13	17.9 ± 10	Previously studied [11]		No
		YPO4022-YPO4025	*fitABCD*	+	34.4 ± 14	39.2 ± 14	Not determined		No
		YPO1343-YPO1345	*fiuABC*	+	35.6 ± 15	27.3 ± 14	Not determined		No
		YPO1310-YPO1313	*yiuABCR*	+	Not determined		0 vs 12.5	4 vs 4	No
		YPO2439-YPO2442	*yfeABCD*	+	Not determined		Previously studied [12]		Yes
		YPO3390-YPO3392	*fhuCDB*	↔	Not determined		Not determined		To be tested
	Permeases	YPO1854-YPO1856	*efeUOB*	+	Not determined		12.5 vs 12.5	4 vs 4.5	No
		YPO1941-YPO1948	-	+	Not determined		0 vs 0	4 vs 4	No
	Heme uptake	YPO0286-YPO0279	*-hmuRSTUV*	+	Not determined		0 vs 0	4.5 vs 5.5	No
		YPO1951-YPO1954	*hmsHFRS*	+	57.4 ± 15	37.8 ± 14	Previously studied [13]		No
		YPO3918-YPO3923	*hasRADEB*	–	Not determined		Previously studied [14]		No
	Ferrous	YPO0131-YPO133	*feoABC*	↔	Not determined		Previously studied [15]		No
Manganese	ABC transporter	YPO2439-YPO2442	*yfeABCD*	+	Not determined		Previously studied [12]		Yes
	Permease	YPO2982	*mntH*	+	Not determined		0 vs 0	4 vs 4	No[ɣ]
Zinc	ABC transporter	YPO2439-YPO2442	*yfeABCD*	+	Not determined		Previously studied [12]		Yes
		YPO2059-YPO2061	*znuBC znuA*	+	Not determined		25 vs 0	6.5 vs 6.5	No[ɣ]

[ə], according to our previous data [16]

[†], ARCIs were determined in groups of 10 rats inoculated with a pool of 5 mutants (with 20 CFU of each mutant)

[‡], virulence was determined after intradermal inoculation of ~10 CFU. Groups of 8 animals were used

[ɣ], confirms recently published work

[ϕ], pseudogene

*, a gene was considered to be necessary ("Yes") or not necessary ("No") for virulence if the survival curve for animals infected with the mutant and the wild-type strain were significantly different ($p<0.05$) or not significantly different ($p>0.05$) in a Gehan-Breslow-Wilcoxon test, or because the mutant was outcompeted (ARCI<20) or not outcompeted (ARCI≥20) in the per-pool screening, or because it has been previously studied elsewhere. In the latter case, a reference (Text S1) is provided.
