# Supplementary material for: New Insights into How Yersinia pestis Adapts to Its Mammalian Host during Bubonic Plague
Source: PLoS Pathog. 2014 Mar 27;10(3):e1004029. doi: 10.1371/journal.ppat.1004029 (PMC3968184; doi:10.1371/journal.ppat.1004029)
Supplement: Table S6 — The role of some Y. pestis genes predicted to protect against RNS and ROS in vivo. (PDF) [file ppat.1004029.s011.pdf]

Table S6. The role of some *Y. pestis* genes predicted to protect against RNS and ROS *in vivo*

	**MUTANT LACKING**		**VIRULENCE DATA OBTAINED USING**				**ROLE IN VIRULENCE***
			**PER POOL MUTANTS**[†]		**INDIVIDUAL MUTANT**[‡]		
			**ARCI ± SE IN**		**SURVIVAL**		
	**ORF(s)**	**GENE(s)**	**LYMPH NODE**	**SPLEEN**	**PERCENT**	**MEDIAN (in days)**	
RNS genes upregulated *in vivo*	YPO2908	*hmp*	Not determined		Previously studied [16]		Yes
	YPO0116	*metL*	Not determined		25 vs 0	4 vs 4	No
	YPO3531	*ytfE*	Not determined		0 vs 0	4 vs 4	No
	YPO1949	*tehB*	Not determined		0 vs 12.5	3 vs 4	No
	YPO0117	*metF*	Not determined		12.5 vs 12.5	5 vs 4	No
	YPO3789-YPO3790	*metR-*	Not determined		12,5 vs 0	4 vs 4	No
	YPO1359	*hcr*	14.9 ± 11	21.2 ± 11	12.5 vs 0	4 vs 4	No
	YPO2652-YPO2648	*nrdHIEF*	Not determined		12.5 vs 0	5 vs 4	Yes
	YPO1528	*fhuF*	Not determined		0 vs 0	4 vs 4	No
	YPO3418-YPO3419	*aceEF*	Not determined		100 vs 12.5	Undefined vs 4	Yes
ROS genes upregulated *in vivo*	YPO2705	*yfiD*	Not determined		0 vs 0	4 vs 4	No
	YPO2982	*mntH*	Not determined		0 vs 0	4 vs 4	No
	YPO2264	*fumC*	39.9 ± 16	30.8 ± 15	Not determined		No
	YPO4085	*ibpA*	0.9 ± 0	1.6 ± 1	Not determined		Yes

†, ARCIs were determined using groups of 10 rats inoculated with a pool of 5 mutants (with 20 CFU of each mutant)

‡, virulence was determined after intradermal inoculation of ~10 CFU. Groups of 8 animals per mutant were used

*, a gene was considered to be necessary ("Yes") or not necessary ("No") for virulence if the survival curve for animals infected with the mutant and the wild-type strain were significantly different ($p<0.05$) or not significantly different ($p>0.05$) in a Gehan-Breslow-Wilcoxon test, or because the mutant was outcompeted (ARCI<20) or not outcompeted (ARCI≥20) in the per-pool screening, or because it has been previously studied elsewhere. In the latter case, a reference (Text S1) is provided.
