# Supplementary material for: New Insights into How Yersinia pestis Adapts to Its Mammalian Host during Bubonic Plague
Source: PLoS Pathog. 2014 Mar 27;10(3):e1004029. doi: 10.1371/journal.ppat.1004029 (PMC3968184; doi:10.1371/journal.ppat.1004029)
Supplement: Text S1 — References for supplementary tables. (DOCX) [file ppat.1004029.s012.docx]

**REFERENCES FOR SUPPLEMENTARY DATA**

1. Welkos SL, Friedlander AM, Davis KJ (1997) Studies on the role of plasminogen activator in systemic infection by virulent *Yersinia pestis* strain C092. Microb Pathog 23: 211-223.

2. Chen TH, Foster LE, Meyer KF (1961) Experimental comparison of the immunogenicity of antigens in the residue of ultrasonated avirulent *Pasteurella pestis* with the vaccine prepared with the killed virulent whole organisms. J Immunol 87: 64-71.

3. Simon R, Priefer U, Puhler A (1983) A broad host range mobilization system for *in vivo* genetic engineering in Gram negative bacteria. Bio/Technology 1: 784-791.

4. Vieira J, Messing J (1982) The pUC plasmids, an M13mp7-derived system for insertion mutagenesis and sequencing with synthetic universal primers. Gene 19: 259-268.

5. Taylor LA, Rose RE (1988) A correction in the nucleotide sequence of the *Tn*903 kanamycin resistance determinant in pUC4K. Nucleic Acids Res 16: 358.

6. Donnenberg MS, Kaper JB (1991) Construction of an *eae* deletion mutant of enteropathogenic *Escherichia coli* by using a positive-selection suicide vector. Infect Immun 59: 4310-4317.

7. Datsenko KA, Wanner BL (2000) One-step inactivation of chromosomal genes in *Escherichia coli* K-12 using PCR products. Proc Natl Acad Sci U S A 97: 6640-6645.

8. Choi KH, Gaynor JB, White KG, Lopez C, Bosio CM, et al. (2005) A *Tn7*-based broad-range bacterial cloning and expression system. Nat Methods 2: 443-448.

9. Achtman M (2012) Insights from genomic comparisons of genetically monomorphic bacterial pathogens. Philos Trans R Soc Lond B Biol Sci 367: 860-867.

10. Iteman I, Guiyoule A, de Almeida AM, Guilvout I, Baranton G, et al. (1993) Relationship between loss of pigmentation and deletion of the chromosomal iron-regulated irp2 gene in *Yersinia pestis*: evidence for separate but related events. Infect Immun 61: 2717-2722.

11. Kirillina O, Bobrov AG, Fetherston JD, Perry RD (2006) Hierarchy of iron uptake systems: Yfu and Yiu are functional in *Yersinia pestis*. Infect Immun 74: 6171-6178.

12. Bearden SW, Perry RD (1999) The Yfe system of *Yersinia pestis* transports iron and manganese and is required for full virulence of plague. Mol Microbiol 32: 403-414.

13. Lillard JW, Jr., Bearden SW, Fetherston JD, Perry RD (1999) The haemin storage (Hms+) phenotype of *Yersinia pestis* is not essential for the pathogenesis of bubonic plague in mammals. Microbiology 145 ( Pt 1): 197-209.

14. Rossi MS, Fetherston JD, Letoffe S, Carniel E, Perry RD, et al. (2001) Identification and characterization of the hemophore-dependent heme acquisition system of *Yersinia pestis*. Infect Immun 69: 6707-6717.

15. Fetherston JD, Mier I, Jr., Truszczynska H, Perry RD (2012) The Yfe and Feo transporters are involved in microaerobic growth and virulence of *Yersinia pestis* in bubonic plague. Infect Immun 80: 3880-3891.

16. Sebbane F, Lemaitre N, Sturdevant DE, Rebeil R, Virtaneva K, et al. (2006) Adaptive response of *Yersinia pestis* to extracellular effectors of innate immunity during bubonic plague. Proc Natl Acad Sci U S A 103: 11766-11771.
